# Design, Synthesis, and Pharmacokinetic Profiling of Fluorinated Reversible *N*‑Alkyl Carbamate Derivatives of Psilocin for Sub-Hallucinogenic Brain Exposure

**DOI:** 10.1021/acs.jmedchem.5c01797

**Published:** 2026-01-26

**Authors:** Marco Banzato, Martina Colognesi, Lorena Lucatello, Stefano Comai, Gianfranco Pasut, Francesca Capolongo, Laura Orian, Lucia Biasutto, Anna Signor, Daniela Gabbia, Paolo L. Manfredi, Sara De Martin, Andrea Mattarei

**Affiliations:** † Department of Pharmaceutical and Pharmacological Sciences, 9308 University of Padova, Via Francesco Marzolo 5, Padua 35131, Italy; ‡ Department of Comparative Biomedicine and Food Science, 9308 University of Padova, Viale dell’Università 16, Legnaro, Padua 35020, Italy; § Department of Biomedical Sciences, 9308 University of Padova, Via Ugo Bassi 58/B, Padua 35131, Italy; ∥ Department of Psychiatry, McGill University, 1033 Pine Avenue West, Montreal, Quebec H3A 1A1, Canada; ⊥ Department of Chemical Sciences, 9308 University of Padova, Via Francesco Marzolo 1, Padua 35131, Italy; # Italian National Research Council (CNR) Neuroscience Institute, Viale Giuseppe Colombo 3, Padua 35131, Italy; ∇ MGGM Therapeutics, 85 Baker Road, Kerhonkson, New York 12446, United States

## Abstract

Psilocybin, the phosphorylated prodrug of psilocin, holds therapeutic promise across a range of neuropsychiatric conditions, yet its clinical utility is constrained by acute psychoactive effects. Here, we report the rational design, synthesis, and evaluation of a focused library of fluorinated reversible *N*-alkyl carbamate derivatives of psilocin aimed at reducing acute psilocin exposure and thereby limiting hallucinogenic-like effects. Carbamate bond stability was systematically modulated by varying the number and positioning of fluorine atoms on the alkyl promoiety. The resulting compounds exhibited finely tuned hydrolysis under physiological conditions. A selected lead compound (4e) showed favorable oral bioavailability and efficient brain penetration while undergoing partial bioconversion to psilocin. Notably, 4e displayed intrinsic serotonergic activity at 5-HT_2A_ and 5-HT_2C_ receptors but induced attenuated psychotropic effects relative to psilocybin. Overall, these findings highlight fluorinated carbamate chemistry as a versatile platform to control psilocin exposure and serotonergic signaling, rather than the development of a classical pharmacologically inert prodrug.

## Graphical abstract

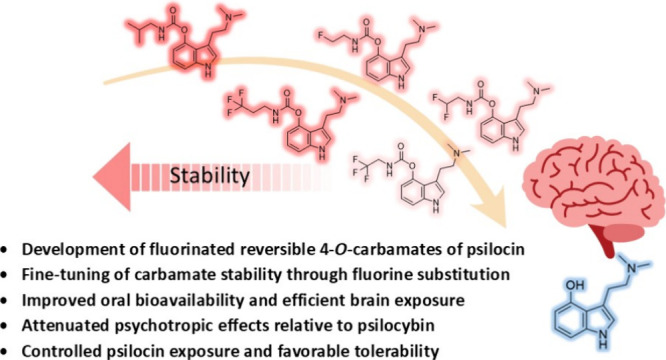

## Introduction

Psychedelic compounds are experiencing renewed interest in biomedical research and drug development.^1^ Accumulating evidence highlights a central role of serotonin (5-HT) signaling pathways in modulating neuroplasticity,^2^ a process with therapeutic implications for a range of neuropsychiatric conditions, including depression,^3,4^ substance use disorder,^5,6^ and neurodegenerative diseases.^7^ Serotonergic psychedelics such as lysergic acid diethylamide (**LSD**), *N*,*N*-dimethyltryptamine (**DMT**) and psilocin (**PSI**), have been shown to increase dendritic complexity, promote spine formation, and stimulate synaptogenesis following a single hallucinogenic dose in rodent models.^8–11^ The molecular mechanisms underlying these effects remain a thriving and constantly evolving area of investigation.^12–15^

Psilocybin (**PSY**), a naturally occurring tryptamine and the 4-*O*-phosphate ester prodrug of **PSI**, has a long but discontinuous history in neuropsychiatric research. **PSY** and related serotonergic psychedelics were extensively investigated in the 1950s and 1960s; however, by the early 1970s, political and cultural pressures led to the discontinuation of nearly all clinical and preclinical research in this field.^16^ Decades later, renewed interest emerged as modern, academically driven research and clinical trials demonstrated the safety and therapeutic potential of **PSY** in mood and anxiety disorders.^17,18^ In parallel with this academic renaissance, **PSY** has become the focus of active clinical development, with multiple ongoing trials and increasing pharmaceutical and biotechnology interest in serotonergic psychedelic therapies.^19^ Notably, placebo-controlled phase II trials conducted at New York and Johns Hopkins Universities reported sustained improvements in anxiety and depression up to six months after a single oral hallucinogenic dose of **PSY**.^20,21^ Similarly, an open-label study in patients with treatment-resistant depression found that two hallucinogenic doses of **PSY** administered 1 week apart resulted in rapid and durable symptom relief, lasting at least three months.^22,23^ However, a subsequent double-blind, randomized, controlled trial comparing **PSY** with the SSRI escitalopram found no significant difference in efficacy, raising questions about its advantage over existing antidepressants.^24^ Indeed, the acute psychotropic effects of **PSY** remain a major concern and have historically limited its clinical potential.^25,26^ Therapeutic discrepancies emerged in the clinical use of **PSY** can be ascribed to the individual variability in its pharmacokinetic profile after oral administration.^27^ **PSY** is rapidly dephosphorylated in the intestinal lumen and during first-pass metabolism to yield **PSI**, with no detectable levels of **PSY** in plasma. **PSI** can undergo Phase I hepatic metabolism through oxidative pathways involving monoamine oxidase A (MAO-A)-mediated deamination followed by aldehyde dehydrogenase (ALDH)-dependent oxidation to the inactive metabolite 4-hydroxyindole-3-acetic acid (**4-HIAA**),^28,29^ as well as through minor cytochrome P450-mediated routes, including CYP2D6.^30,31^ However, the predominant metabolic clearance pathway of **PSI** is Phase II conjugation, namely efficient glucuronidation by the UDP-glucuronosyltransferase (UGT) family, yielding psilocin-*O*-glucuronide as the major urinary metabolite. This extensive conjugative metabolism results in a short plasma half-life of approximately 3 h.^28,32,33^ The phosphate group in **PSY** protects the 4-hydroxy (4-OH) moiety of **PSI** from glucuronidation during absorption, enabling systemic delivery of unconjugated **PSI** and reducing the dose needed to elicit a therapeutic response.

Although previous reports have described serotonergic indole derivatives,^34–36^ few studies have systematically evaluated the impact of protecting groups at 4-OH position of **PSI** on oral pharmacokinetics. A notable early example is a Sandoz patent by Albert Hofmann, claiming *O*-acetylpsilocin (psilacetin) and other 4-*O*-alkyl esters.^37^ However, these derivatives were not clinically developed, as they do not appear to offer substantial pharmacokinetic or pharmacological advantages over psilocybin, as recently reported for the psilacetin derivative.^38^ More recently, a compelling manuscript by Raithatha et al. reported benzyl ester and benzyl thiocarbonate prodrugs of **PSI** that achieved therapeutically relevant plasma levels, neuroactive responses, and behavioral efficacy in mouse models.^39^ Eklund et al. further investigated ester-based **PSI** prodrugs and **PSI** salts as scalable and cost-effective alternatives to **PSY**, with comparable efficacy.^40^ Of particular relevance to this study, they were the first to disclose a 4-*O*-carbamate **PSI** prodrug; however, the resulting compound exhibited excessive chemical and enzymatic stability, limiting its suitability as a pharmacologically viable prodrug.

Importantly, over the last 20 years, the practice of “microdosing”—chronic administration of subhallucinogenic psychedelic doses (typically ≈1/10th of a standard psychoactive dose)—has gained widespread attention.^41,42^ Based on anecdotal reports and uncontrolled observational studies,^43–47^ microdosing has been associated with perceived improvements in energy, mood, cognition, anxiety, and creativity. More recent controlled studies have begun to validate these claims for **LSD** ^48–51^ and **PSY** ^52–54^ in both preclinical and clinical contexts, underscoring a growing interest for the potential clinical applications of serotonergic modulators at nonpsychedelic doses or formulations. However, the current paucity of controlled trials has determined a lack of scientific consensus regarding the efficacy of microdosing strategies.^55–57^

This emerging paradigm led us to design a new class of **PSI** derivatives capable of (1) protecting the 4-OH group from Phase II glucuronidation during absorption, (2) facilitating blood-brain barrier (BBB) penetration, and (3) enabling sustained, subhallucinogenic release of **PSI**. We hypothesized that low-level, continuous delivery of **PSI** to the central nervous system (CNS) could enhance its neuroplastic effects while minimizing undesirable psychotropic activity.

Herein, we report the rational design, synthesis, and characterization of a focused library of five 4-*O*-(*N*-alkyl carbamate) **PSI** derivatives. Carbamate esters of phenolic drugs have previously been shown to improve oral absorption, reduce Phase II metabolism, and enable sustained CNS release.^58,59^ To enhance BBB permeability, we employed small, lipophilic alkyl promoieties and modulated their stability by strategic replacement of hydrogen atoms with fluorine atoms at different positions in the aliphatic promoiety. We assessed the chemical stability of the synthesized compounds in physiologically relevant pH environments, their metabolic stability in human liver microsomes (HLMs) and S9 fractions and their degradation kinetics in human plasma. Based on these findings, a lead compound was selected for further in vivo characterization of its ADME, toxicity, and pharmacodynamic properties.

## Results and Discussion

### Rational Design and In Silico DFT Studies of Psilocin Reversible Carbamate Derivatives

Electronic and physicochemical parameters were considered in the rational design of reversible 4-*O*-carbamate **PSI** derivatives. Lipophilicity and low molecular weight—critical for blood–brain barrier (BBB) permeability—were prioritized, in line with extensive literature supporting their importance for CNS-targeting agents.^60^ In fact, the BBB is characterized by tight junctions between endothelial cells of brain capillaries, resulting in a semipermeable barrier that prevent paracellular transport but permits passive diffusion of suitably lipophilic, low-molecular-weight compounds, as well as active transport of specific substrates via dedicated transporters.^61^

An optimal reversible **PSI** derivative must strike a balance between chemical stability and lability: it should resist premature hydrolysis and Phase II conjugation (notably glucuronidation) during absorption and first-pass metabolism, yet it should release **PSI** at a rate sufficient to produce sustained pharmacological activity before undergoing further systemic and central metabolism. To this end, we designed a series of 4-*O*-(*N*-alkyl carbamates) of **PSI**, varying the degree of electron-withdrawing substitution on the aliphatic chain of the promoiety to fine-tune carbamate hydrolysis kinetics. In fact, base-induced hydrolysis of *N*-monosubstituted carbamates proceeds via deprotonation of the nitrogen atom, followed by elimination to form an isocyanate intermediate which rapidly adds water and decomposes releasing carbon dioxide and the free amine (Scheme 1).^62^

**Scheme 1:**
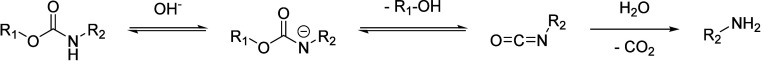
Base-Induced Hydrolysis Mechanism of *N*-Monosubstituted Carbamate Esters

Increasing the electron-withdrawing character around the carbamate nitrogen accelerates this rate-determining deprotonation step, resulting in faster hydrolysis rate. Based on these preliminary considerations, we chose to strategically take advantage of the small and highly electronegative fluorine atom to rationally impart an increasingly strong electron withdrawing effect on the carbamate *N*-atom of the resulting **PSI** derivatives (Figure 1A), while maintaining a 350 Da MW cutoff and suitable lipophilicity for BBB penetration. To confirm the specific destabilizing effect of fluorine atoms on the urethane bond, we included a nonfluorinated isobutyl carbamate as a control.

**Figure 1:**
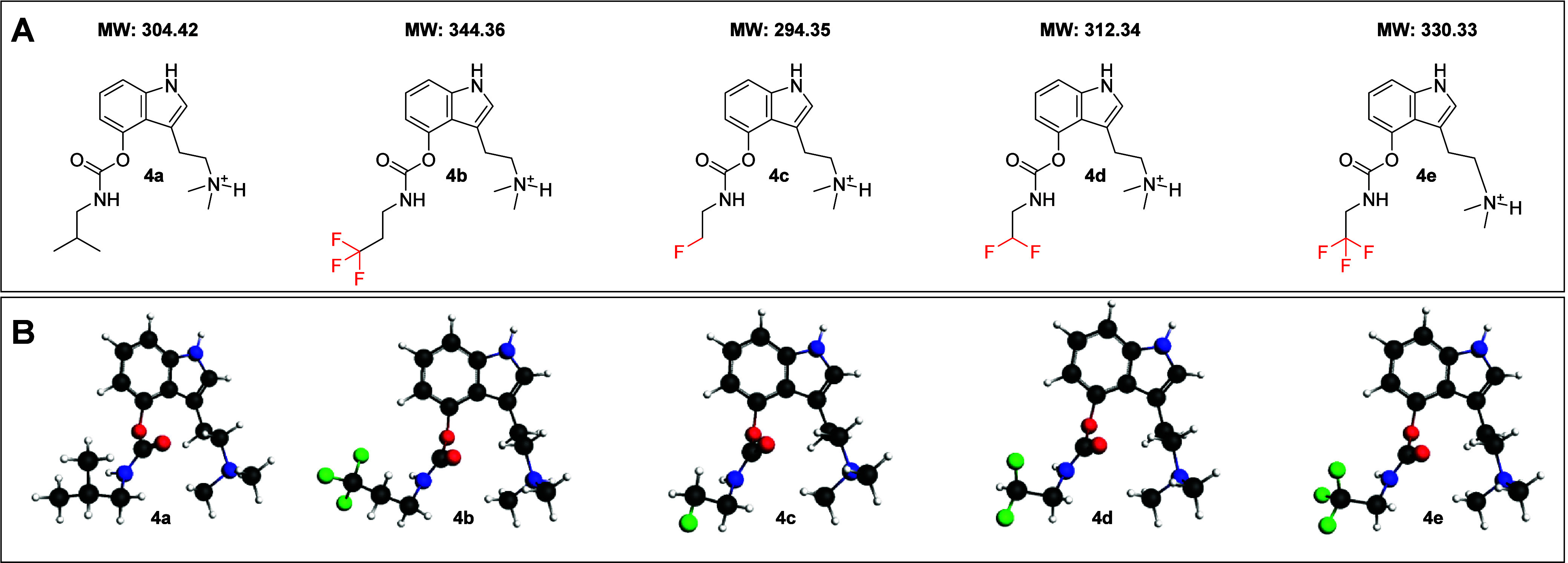
(A) Chemical structures of the designed **PSI** carbamate derivatives **4a–e**. (B) Fully optimized molecular structures of compounds **4a–e**; level of theory: BLYP-D3­(BJ)/TZ2P.

To predict stability trends, we performed a DFT-based in silico analysis. Structures of compounds **4a–e** were fully optimized at BLYP-D3­(BJ)/TZ2P level of theory (see computational details in Supporting Information S44). Consistent with the reported p*K*_a_ of **PSI**,^63^ the dimethylamino group of the tryptamine scaffold was modeled in its protonated form. For carbamates **4a** and **4b**, which bear relatively long and flexible alkyl substituents (trifluoropropyl and isobutyl groups, respectively), greater conformational freedom was expected. However, to enable a meaningful and direct comparison among the synthesized compounds, we selected conformers structurally related to those of compounds **4c–e**—specifically, those in which the alkyl chain extends in the opposite direction relative to the dimethylamino group (Figure 1B).

The corresponding carbamate *N*-deprotonated forms were also fully optimized using the same computational protocol. The energy differences (Δ*E*) between the neutral and anionic forms (Table 1) were used as proxies for the thermodynamic tendency toward deprotonation.^64^ In fact, while absolute p*K*_a_ values determination in solution require complex modeling, these Δ*E* values provided a meaningful comparative measure of carbamate acidity across the series.

**Table 1:** Energy Difference between the Carbamate *N*-Atom Deprotonated Species and the Corresponding Parent Compound^a^

compound	Δ*E* (kcal mol^–1^)
**4a**	–3.40
**4b**	–7.50
**4c**	–9.29
**4d**	–11.89
**4e**	–18.33

a Level of theory: BLYP-D3­(BJ)/TZ2P.

A progressive and significant increase in the acidity of the carbamate NH proton (i.e., more negative Δ*E*) was observed from compound **4a** to **4e**, consistent with the increasing electron-withdrawing character of the substituents on the carbamate chain. These findings supported a systematic trend in chemical lability across the series and provided a strong rationale for the experimental synthesis of the designed **PSI** carbamate derivatives.

### Chemical Synthesis

**PSI** was synthesized from commercially available 4-(benzyloxy)-1*H*-indole (**BOI**) following the procedure reported by Raithatha et al.^39^ with slight modifications. Our optimized route (Scheme 2) significantly improved overall yield (68 vs 38%). In particular, formation of an undesired indoline byproduct in the final Pd-catalyzed hydrogenation step was minimized by replacing gaseous H_2_ with ammonium formate under reflux, enabling more precise reaction control.

**Scheme 2:**
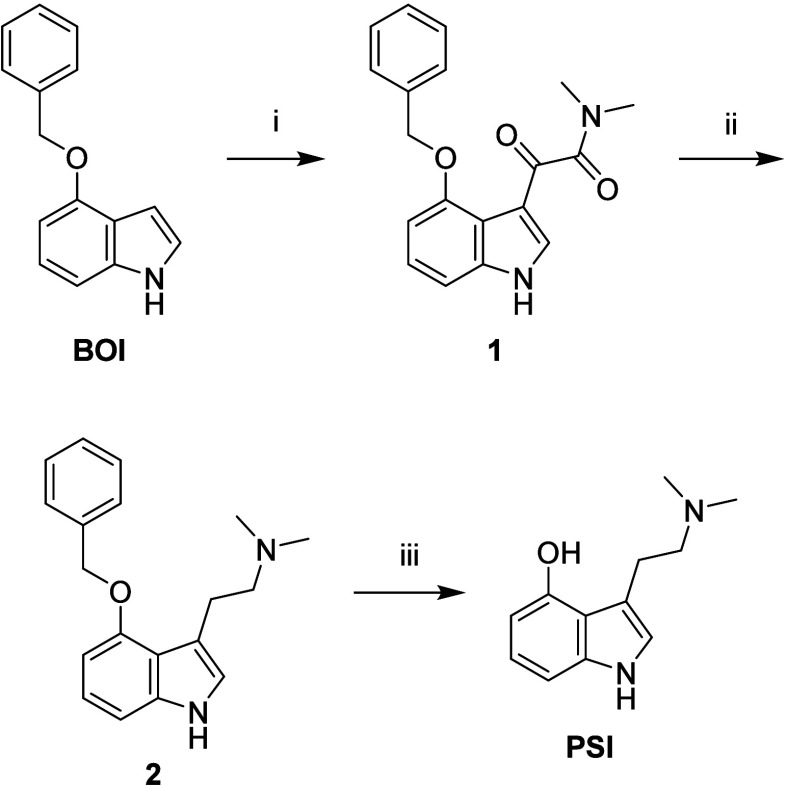
Reagents and Conditions: (i) (COCl)_2_, Et_2_O, 0 °C, 1 h, then Me_2_NH, 83%; (ii) LiAlH_4_, THF, 0 °C to rt, 1 h, Then Reflux, 16 h, 88%; (iii) Pd/C, NH_4_HCO_2_, MeOH, 20 min, 93%

We then developed a two-step, one-pot method for the synthesis of **PSY** from **PSI** (Scheme 3) modifying established procedures.^65–67^

**Scheme 3:**
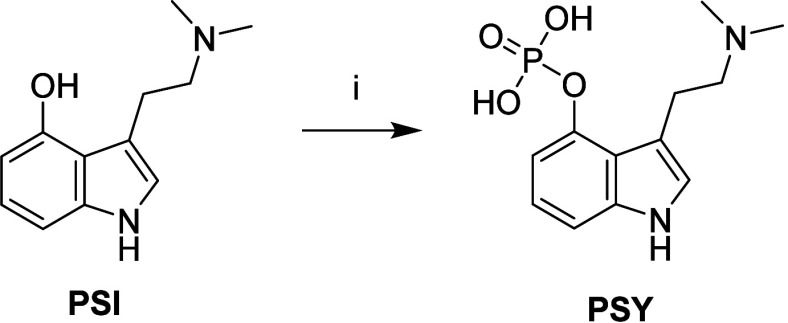
Reagents and Conditions: (i) LDA, TBPP, −78 to −10 °C, 2 h; Then Pd/C, H_2_, MeOH, rt, 2 h, 85%

In detail, the phosphorylation of **PSI** using tetrabenzylpyrophosphate (**TBPP**) and lithium diisopropylamide (LDA) typically yields complex mixtures of benzylated intermediates due to intramolecular *O*-to-*N* benzyl group migration.^66,67^ Previous strategies aimed at isolating specific zwitterionic phosphate intermediates were labor-intensive and poorly reproducible. In our streamlined approach, quenching the phosphorylation reaction with a saturated solution of ammonium chloride followed by extraction into ethyl acetate (EtOAc) yielded a crude mixture that, upon Pd-catalyzed hydrogenolysis, cleanly furnished **PSY**. This one-pot procedure afforded the highest reported yield for **PSY** synthesis (85%) while significantly simplifying the overall workflow. Importantly, the method proved to be highly reproducible, scalable, and compatible with standard laboratory conditions, thus offering a practical and efficient route to **PSY** for both research and development purposes.

**PSI** 4-*O*-carbamate derivatives were synthesized via a well-established^58,68^ two-step sequence (Scheme 4).

**Scheme 4:**
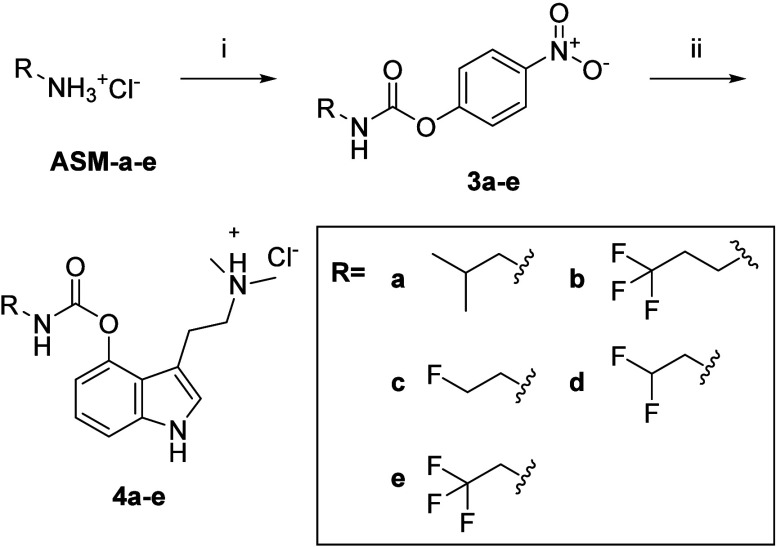
Reagents and Conditions: (i) BNPC, DMAP, DCM, 0 °C, 1 h, 70–93%; (ii) **PSI**, DMAP, THF, rt, 16 h, 78–88%

First, the primary amine starting material (**ASM**) of each alkyl promoiety (**a–e**) was reacted with bis­(4-nitrophenyl) carbonate (BNPC) to generate the corresponding activated urethanes (**3a–e**). These intermediates were then coupled to **PSI** under mild transesterification conditions to afford the final **PSI** 4-*O*-carbamate derivatives (**4a–e**) in good overall yield.

### In Vitro Evaluation of Psilocin Carbamate Derivatives: Chemical, Plasma, and Metabolic Stability

The stability of **PSI** 4-*O*-carbamate derivatives was evaluated in aqueous buffers reproducing the pH conditions of the gastrointestinal tract and compared to that of **PSY**. All tested compounds remained stable for at least 24 h (see Supporting Information S33) in acidic media (0.1 M HCl, 37 °C), used here as a chemical proxy of the gastric environment. In contrast, hydrolysis to **PSI** was observed under near-neutral intestinal conditions (pH 6.8 and 7.4, see Supporting Information S35 and S37), with faster hydrolysis rates at pH 7.4 (Table 2). Consistent with previous reports, **PSY** remained stable under all in vitro conditions tested.^39^ As anticipated, electron-withdrawing substituents on the carbamate promoiety accelerated hydrolysis, with degradation rates following the order: **4a** < **4b** < **4c** < **4d** < **4e**. These findings are in strong agreement with the DFT calculations, which predicted increased carbamate acidity and corresponding bond destabilization across the series, resulting in a reduction of the half-life by approximately 1 order of magnitude from the least (**4a**) to the most (**4e**) labile compound. Plasma stability was similarly influenced by electron-withdrawing substituents, with degradation rates increasing from compound **4a** to **4e**, consistent with trends observed in PBS at pH 7.4. Notably, hydrolysis proceeded 5–10 times faster in plasma than in buffer (see Supporting Information S39), suggesting a significant contribution from enzymatic processes. However, since the magnitude of this enzymatic effect was comparable across all the synthesized compounds—as reflected by the consistent ratio between hydrolysis rate constants in PBS (pH 7.4) and in plasma—chemical hydrolysis remains the main determinant of relative lability within the series, justifying the comparative use of DFT-derived acidity parameters.

**Table 2:** Observed Pseudo-First-Order Hydrolysis Rate Constants of Compounds **4a–e** Determined in Aqueous PBS at pH 6.8, pH 7.4, and in Human Plasma^a^

	PBS 0.1 M, pH 6.8, 37 °C		PBS 0.1 M, pH 7.4, 37 °C		plasma, 37 °C	
compound	***k* (h** ^**–1**^ **) × 10** ^**3**^	***t***_**1/2**_ (h)	***k* (h** ^**–1**^ **) × 10** ^**3**^	***t***_**1/2**_ (h)	***k* (h** ^**–1**^ **) × 10** ^**3**^	***t***_**1/2**_ (h)
**PSY**	0	∞	0	∞	0	∞
**4a**	1.1 ± 0.2	648	5.7 ± 0.2	122	66 ± 2	11
**4b**	3.0 ± 0.2	232	12.5 ± 0.4	56	98 ± 4	7
**4c**	4.0 ± 0.2	172	16.6 ± 0.7	42	87 ± 2	8
**4d**	7.9 ± 0.3	87	33.0 ± 1.0	21	200 ± 7	3
**4e**	15.1 ± 0.4	46	62.0 ± 2.0	11	297 ± 4	2

a Rate constants were obtained by best fitting the experimental data to a first-order exponential equation, [*C*] = [*C*]_0_ × e^–*kt*^. Data are reported as mean ± standard error (see Experimental Data in Supporting Information S33–S41).

Similar conclusions were drawn regarding metabolic stability. As shown in Table 3, **PSY** and most of the synthesized compounds remained essentially stable, with concentrations decreasing by less than 30% following 360 min of incubation with human liver microsomes (HLMs) and cytosolic (S9) fractions. A partial exception was compound **4e**, which showed a 40 and 24% reduction in concentration upon incubation with HLMs and S9, respectively, in line with its previously determined hydrolysis rate at physiological pH. It should be noted that the chemical, plasma, and metabolic stability evaluations described above were intended as preliminary screening assays to assess the relative stability of the synthesized compounds. We acknowledge that these experiments were conducted with limited replication (*n* = 3 per condition), which may constrain statistical robustness. Nevertheless, the consistency of the results across conditions provides a degree of confidence that supported downstream lead selection.

**Table 3:** Metabolic Stability of **PSY** and Compounds **4a–e** Following Incubation with Human Liver Microsomes (HLMs) or Cytosolic (S9) Fractions^a^

	HLMs							
compound	**0 min**	**10 min**	**20 min**	**30 min**	**60 min**	**120 min**	**240 min**	**360 min**
**PSY**	100 ± 41	92 ± 31	98 ± 26	118 ± 20	123 ± 4	116 ± 8	124 ± 21	119 ± 7
**4a**	100 ± 6	105 ± 4	102 ± 3	98 ± 9	96 ± 4	87 ± 7	81 ± 5	73 ± 3
**4b**	100 ± 3	92 ± 2	95 ± 2	92 ± 6	90 ± 8	75 ± 3	78 ± 2	64 ± 4
**4c**	100 ± 3	95 ± 6	96 ± 3	94 ± 4	99 ± 5	94 ± 6	92 ± 3	80 ± 5
**4d**	100 ± 13	95 ± 5	94 ± 1	85 ± 5	82 ± 5	75 ± 10	83 ± 18	70 ± 8
**4e**	100 ± 10	95 ± 6	93 ± 5	93 ± 8	80 ± 5	82 ± 2	66 ± 2	60 ± 7

	S9 fraction							
compound	**0 min**	**10 min**	**20 min**	**30 min**	**60 min**	**120 min**	**240 min**	**360 min**
**PSY**	100 ± 17	102 ± 8	99 ± 17	100 ± 8	110 ± 19	97 ± 11	95 ± 7	103 ± 13
**4a**	100 ± 3	99 ± 4	98 ± 1	96 ± 4	93 ± 10	94 ± 3	85 ± 13	86 ± 2
**4b**	100 ± 3	101 ± 6	98 ± 5	102 ± 4	100 ± 4	89 ± 4	88 ± 1	88 ± 1
**4c**	100 ± 9	103 ± 8	106 ± 5	106 ± 4	105 ± 8	101 ± 1	95 ± 2	96 ± 1
**4d**	100 ± 2	96 ± 0	89 ± 4	94 ± 4	88 ± 4	83 ± 2	79 ± 3	76 ± 1
**4e**	100 ± 4	102 ± 7	98 ± 4	98 ± 7	95 ± 4	87 ± 4	91 ± 7	76 ± 17

a Data are reported as mean percentage of residual compound ± SD (see Experimental Data in Supporting Information S32).

Based on the in vitro findings, compound **4e** was selected as the lead candidate, as it offered the best compromise between chemical stability, metabolic protection, and controlled release. In particular, **4e** demonstrated sufficient stability under conditions mimicking gastrointestinal absorption and hepatic first-pass metabolism to protect the phenolic hydroxyl group of psilocin from extensive Phase II glucuronidation — one of its main metabolic pathways. At the same time, its moderate plasma lability is expected to favor **PSI** release while competing with oxidative metabolic pathways. Such a controlled release profile is expected to mitigate peak plasma concentrations and reduce both the onset and intensity of acute psychedelic effects.

### In Vitro Pharmacodynamic Evaluation of Compound **4e**

The functional activity of compound **4e** was evaluated at the three human 5-HT_2_ receptor subtypes using in vitro fluorometric imaging plate reader (FLIPR) Ca^2+^-mobilization assays. Notably, **4e** produced a clear, concentration-dependent increase in intracellular calcium levels in Chinese hamster ovary (CHO-K1) cells overexpressing 5-HT_2A_ and 5-HT_2C_ receptors, consistent with agonist behavior (Figure S11). The compound exhibited mean pEC_50_ values of 7.79 ± 0.13 at 5-HT_2A_ and 7.10 ± 0.02 at 5-HT_2C_, with corresponding mean maximal responses (*E*_max_) of 33.2 ± 0.9% and 40.9 ± 1.6% relative to the reference agonist serotonin (Table 4). In contrast, no measurable agonist response was observed at the 5-HT_2B_ receptor. These results indicate that **4e** acts as a selective partial agonist at 5-HT_2A_ and 5-HT_2C_ receptors, with no intrinsic efficacy at 5-HT_2B_. Its serotonergic profile closely mirrors that of psilocin (used as a control compound),^69^ albeit with slightly lower potency and efficacy at the 5-HT_2A_ and 5-HT_2C_ subtypes.

**Table 4:** Summary of Intracellular Calcium Mobilization Measured at Human 5-HT_2A_, 5-HT_2B_, and 5-HT_2C_ Receptors Measured Using the FLIPR Assay under Agonist Conditions^a^

	5-HT_2A_		5-HT_2B_		5-HT_2C_	
compound	**pEC**_**50**_	***E*_max_ %**	**pEC**_**50**_	***E*_max_ %**	**pEC**_**50**_	***E*_max_ %**
**5-HT**	8.61 ± 0.14	98.8 ± 6.0	9.09 ± 0.09	95.3 ± 0.4	9.12 ± 0.01	113.2 ± 3.6
**4e**	7.79 ± 0.13	33.2 ± 0.9	<4.30		7.10 ± 0.02	40.9 ± 1.6
**PSI**	7.95 ± 0.07	52.4 ± 4.0	<4.30		7.38 ± 0.35	58.6 ± 38.4

a Data for **5-HT** (positive control), **PSI**, and compound **4e** are reported as pEC_50_ and *E*_max_ (%) values (mean ± SD).

### In Vivo Pharmacokinetic Evaluation of Compound **4e**

The pharmacokinetic profile of compound **4e** and its active metabolite **PSI** was evaluated in mice following oral administration, by measuring their levels in both plasma and brain over a 48 h period (see Analytical Method Validation in Supporting Information S22). Plasma concentration-vs-time curves are presented in Figure 2A. Compound **4e** exhibited rapid systemic absorption, with peak concentrations observed in plasma and brain between 1 and 2 h postadministration. Interestingly, brain levels of **4e** exceeded plasma levels across nearly all time points, suggesting efficient central nervous system (CNS) penetration, potentially via passive diffusion across the blood–brain barrier (BBB), as already known for **PSI**.^70^ This observation suggests that the brain retention of compound **4e** may, at least in part, result from its engagement with 5-HT_2A_ and 5-HT_2C_ receptors, indicating that receptor binding could contribute to its prolonged presence within the CNS, possibly influencing its distribution and duration of action.

**Figure 2:**
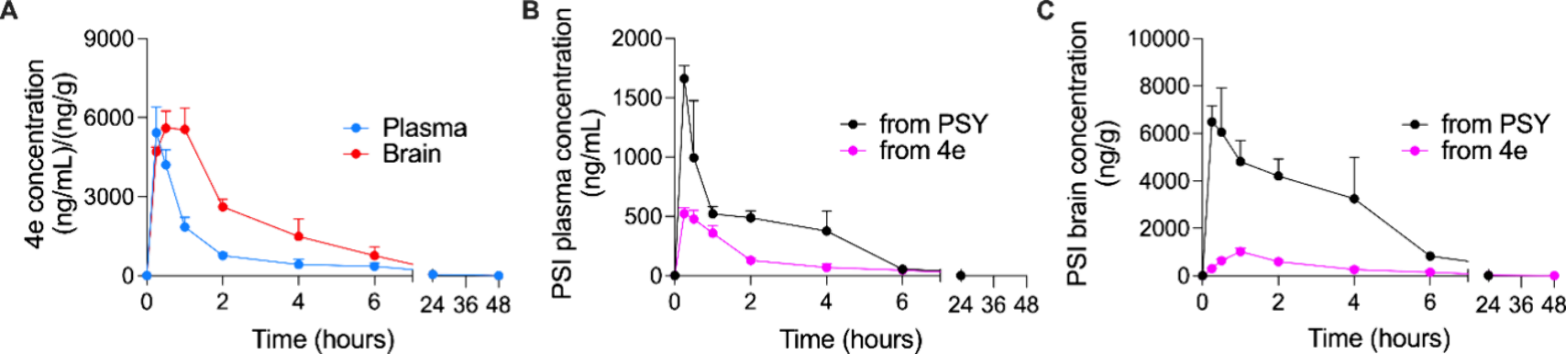
(A) Concentration–time profile of compound **4e** in plasma (blue line) and brain (red line) after oral administration. (B) Comparison of **PSI** plasma concentration after the administration of **PSY** (black line) and the equivalent dose of **4e** (pink line). (C) Comparison of **PSI** concentration in brain tissue after the administration of **PSY** (black line) and the equivalent dose of **4e** (pink line).

Figure 2B depicts plasma concentrations of the active compound **PSI** following administration of either **PSY** or **4e**. **PSY** resulted in significantly higher systemic **PSI** exposure, characterized by both an elevated *C*_max_ and prolonged plasma concentrations of **PSI** compared to **4e**. These data suggest that **4e** may offer a reduced risk of eliciting acute hallucinogenic effects due to lower disposition of **PSI** in the brain. This interpretation is further supported by the brain concentration profiles depicted in Figure 2C, where **PSI** levels were consistently higher following **PSY** administration. Notably, **PSY** produced a broader and more sustained brain exposure of **PSI**, as reflected by a larger area under the curve (AUC), which is consistent with rapid CNS delivery thereby contributing to more pronounced pharmacodynamic effects. In contrast, **4e** exhibited efficient brain penetration as the intact derivative and was associated with a delayed and attenuated **PSI** exposure, as reflected by the pharmacokinetic parameters of **4e**-derived **PSI** (Table 5).

**Table 5:** Pharmacokinetic Parameters of Compound **4e**, **PSI** Derived from **4e**, and **PSI** Derived from **PSY** in Plasma and Brain^a^

	4e		PSI (from 4e)		PSI (from PSY)	
PK parameter	**plasma**	**brain**	**plasma**	**brain**	**plasma**	**brain**
AUC (ng/mL·h)	8903 ± 799	17816 ± 2103	1019 ± 117	3455 ± 340	3866 ± 625	37049 ± 6270
*C*_max_ (ng/mL)/(ng/g)	5426 ± 983	5595 ± 653	521 ± 87	1018 ± 272	1775 ± 240	7769 ± 2017
*T*_max_ (h)	0.25 (0.25)	0.50 (0.25)	0.50 (0.75)	1.00 (0)	0.25 (0.25)	0.25 (0.25)

a Data are reported as mean ± SD for AUC and *C*_max_, and as median (range) for *T*_max_.

To gain insight into the mechanisms contributing to the rapid clearance of this **PSI** derivative, we conducted a preliminary investigation of the possible alternative metabolic pathways of compound **4e**. Since glucuronidation was likely prevented by protection of the phenolic hydroxyl group, we hypothesized that its elimination might instead result from oxidative metabolism. To verify this hypothesis, plasma samples from mice treated with **4e** were analyzed for the presence of the putative metabolite 4-trifluoroethyl carbamate indoleacetic acid (**4-TFEC-IAA**), which was qualitatively identified and remained detectable throughout the pharmacokinetic study period (see Figures S12 and S13). This observation supports the hypothesis that oxidative deamination represents a major metabolic pathway for **4e** in vivo, contributing to its overall clearance alongside enzymatic and chemical hydrolysis processes.

### In Vivo Pharmacodynamic Evaluation of Compound **4e**

Figure 3 presents a comparative analysis of the head-twitch response (HTR), a well-established behavioral correlate of psychedelic activity in rodents,^71–73^ following administration of **PSY** or compound **4e**. As shown in Figure 3A, **PSY** elicited a significantly higher number of head-twitches relative to the vehicle-treated control group. As expected, this effect was dose-dependent, with mice treated with the higher dose (3 mg/kg) exhibiting significantly more HTRs (*p* < 0.05) than those receiving the lower dose (1 mg/kg). Treatment with compound **4e** (3 mg/kg) resulted in a significantly lower number of HTRs compared to both **PSY** doses (*p* < 0.0001).

**Figure 3:**
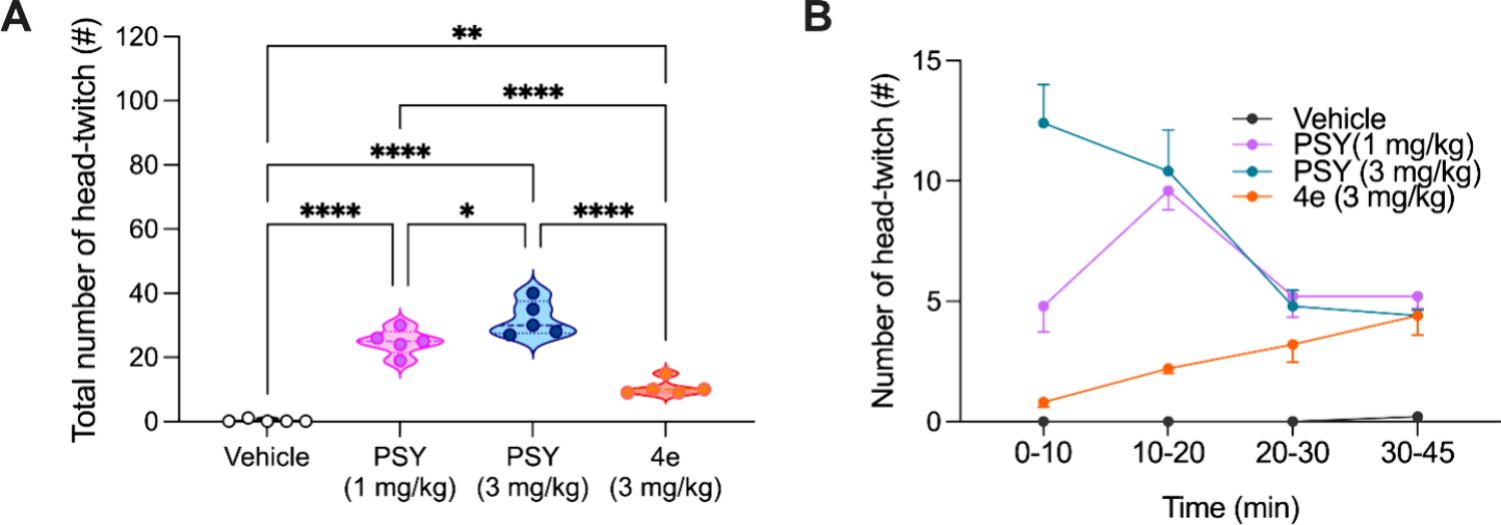
(A) Violin plots reporting the total number of HTRs (#) observed over the entire 45 min period following treatment with vehicle (control), **PSY** at two doses (1 or 3 mg/kg), or the compound **4e** (3 mg/kg). Data are represented as mean and quartiles. *p < 0.05; **p < 0.01; ****p < 0.0001, One-way Anova analysis followed by the post hoc Newman–Keuls test. (B) Time-course analysis of the number of HTRs divided into four intervals: 0–10, 10–20, 20–30, and 30–45 min postadministration for each treatment group. Data are shown for vehicle (black dots), **PSY** (violet dots when used at 1 mg/kg, blue dots when used at 3 mg/kg), and compound **4e** (orange dots).

Figure 3B illustrates the temporal distribution of HTRs, segmented into four-time intervals (0–10, 10–20, 20–30, and 30–45 min). **PSY** consistently induced the highest HTR counts, particularly during the early time windows (0–10 min at 3 mg/kg and 0–20 min at 1 mg/kg). In contrast, compound **4e** did not elicit an early peak response and displayed a delayed and attenuated HTR profile, with a modest increase observed primarily in the intermediate and later time intervals. Vehicle-treated animals showed virtually no response throughout the observation period.

Within the 45 min observation window, these pharmacodynamic findings are consistent with the pharmacokinetic profile of the **PSI** released from **4e**, characterized by lower peak brain concentrations and a delayed *T*_max_ relative to **PSI** released from **PSY**. Together, these data indicate that **4e** produces a delayed and attenuated behavioral response compared to **PSY** under the tested conditions. Although an upward trend in HTRs was observed at later time points within this window, HTR events beyond 45 min were sporadic and infrequent in both **PSY**- and **4e**-treated animals, precluding a reliable quantitative analysis at later intervals. For this reason, the observation period was limited to 45 min. This limitation has been taken into account in the interpretation of the data.

In addition, functional assays demonstrated that **4e** acts as a partial agonist at 5-HT_2A_ and 5-HT_2C_ receptors. The combination of intrinsic serotonergic activity, altered pharmacokinetics, and reduced acute behavioral output suggests that the attenuated HTR observed for **4e** may arise from a complex interplay between delayed and limited **PSI** exposure and the pharmacological properties of the parent compound. While speculative at this stage, these observations are consistent with a context-dependent serotonergic signaling profile, reflecting differences in exposure kinetics and receptor engagement relative to **PSY**. Further studies will be required to clarify the mechanistic basis of these effects.

### In Vivo Toxicological Evaluation of Compound **4e**

A preliminary toxicological assessment of compound **4e** was conducted following a single oral administration at a high dose (100 mg/kg) in rats. Rats were selected due to their established translational relevance for general toxicological studies.^74^ The evaluation focused on hematological parameters and standard biochemical markers of liver and kidney function. In addition, histopathological analyses were performed on the liver and kidneys, given their known susceptibility to drug-induced toxicity, choroid plexus, being the most likely target organ of **4e**, as well as on the heart and lungs.

No significant alterations were observed in blood cell counts (Table 6), or in the histological appearance of liver (Figure 4), kidney (Figure 5), brain (Figure 6), heart and lungs (Figures S14 and S15) tissues. Similarly, liver and renal function markers remained within normal ranges. These findings, although preliminary, suggest that compound **4e** is well tolerated at high doses and support its continued development as a CNS-targeted **PSI** derivative.

**Table 6:** Hematological Parameters in Rats Treated with Vehicle or Compound **4e** ^a^

	vehicle	4e
erythrocytes (×10^12^/L)	5.98 ± 0.09	5.83 ± 0.35
hemoglobin (g/L)	119 ± 1	119 ± 6
hematocrit (L/L)	0.39 ± 0.05	0.39 ± 0.02
MCV (Fl)	65.4 ± 0.8	67.3 ± 1.6
MCH (pg)	19.9 ± 0.1	20.5 ± 0.4
MCHC (g/L)	305 ± 2	305 ± 4
RDW %	11.2 ± 0.2	11.1 ± 0.1
platelets (×10^9^/L)	861 ± 65	857 ± 70
leukocytes (×10^9^/L)	5.76 ± 2.24	5.92 ± 0.47
neutrophils (×10^9^/L)	0.49 ± 0.12	0.59 ± 0.18
lymphocytes (×10^9^ L)	5.19 ± 2.15	5.22 ± 0.39
monocytes (×10^9^/L)	0.03 ± 0.01	0.04 ± 0.01
eosinophils (×10^9^/L)	0.05 ± 0.01	0.06 ± 0.01

a Data are presented as mean ± SD (*n* = 3 per group).

**Figure 4:**
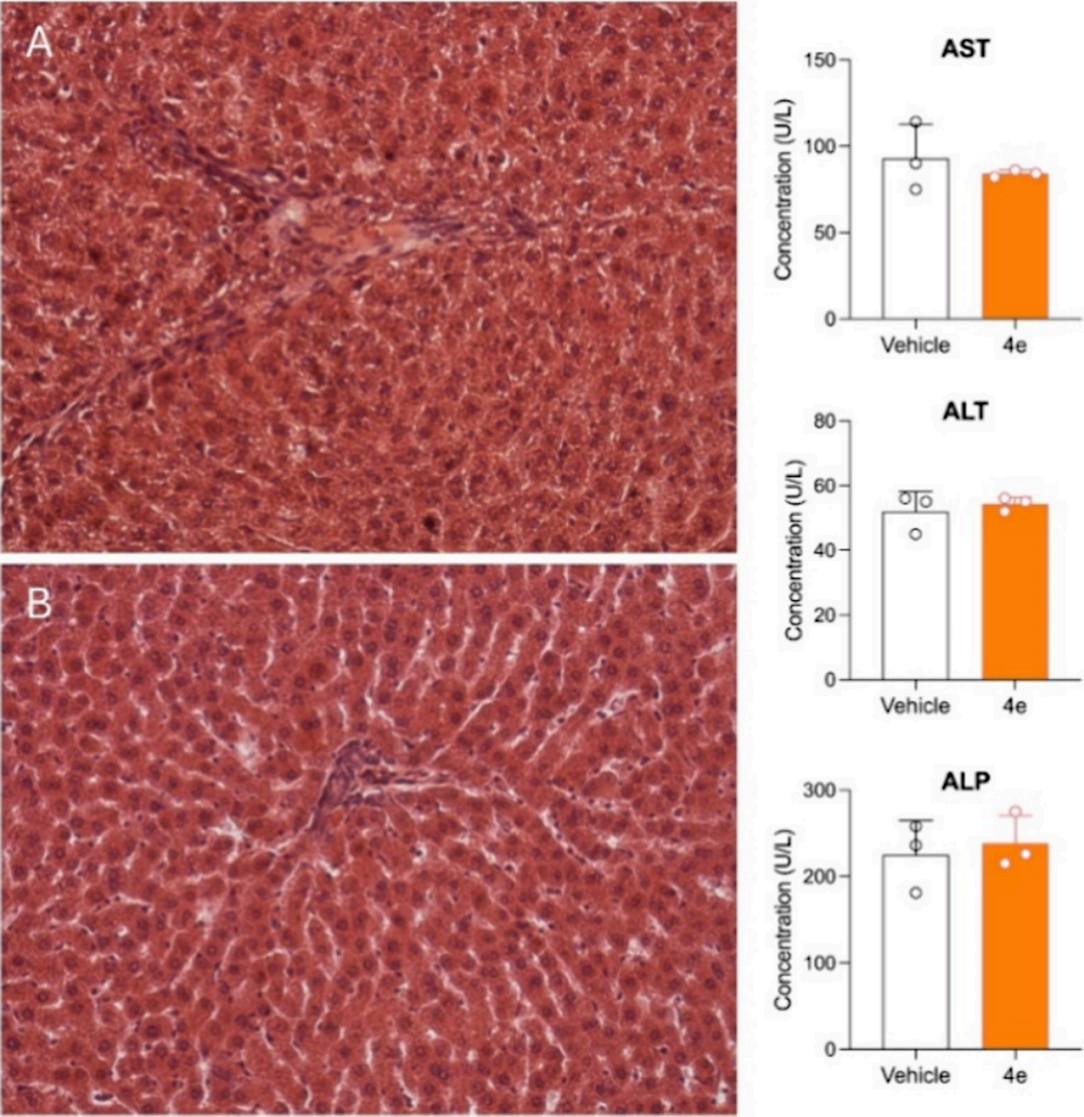
In the left panel, representative liver histology (hematoxylin and eosin staining, 20× magnification) of a rat treated with vehicle (A) or with compound **4e** (B). In the right panel, plasma concentration of three markers of liver function (ALT, AST, and ALP).

**Figure 5:**
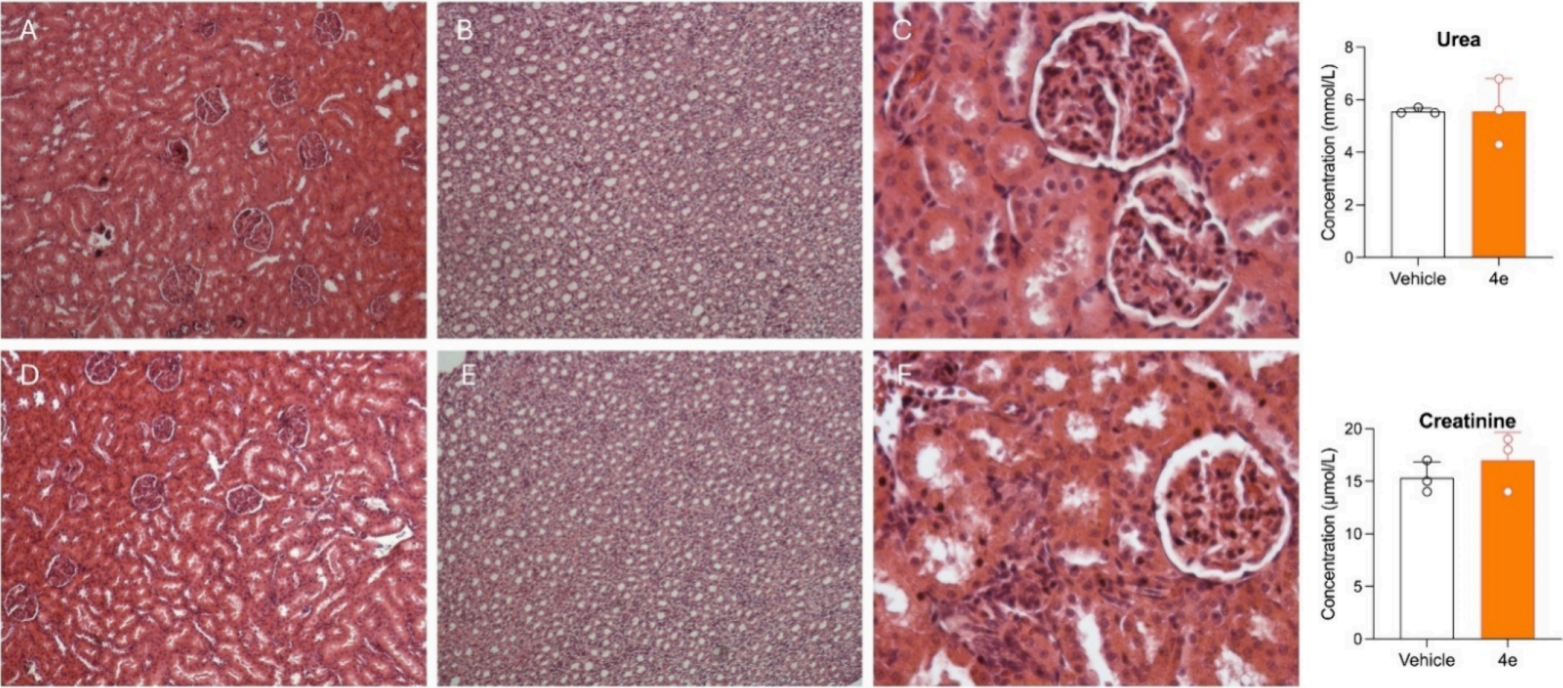
In the left panel, representative kidney histology, reporting the cortex (A, D), the medulla (B, E) (hematoxylin and eosin staining, 10× magnification), and glomeruli (C, F) (hematoxylin and eosin staining, 40× magnification) of a rat treated with vehicle (A–C) or with compound **4e** (D–F). In the right panel, plasma concentration of three markers of kidney function (urea and creatinine).

**Figure 6:**
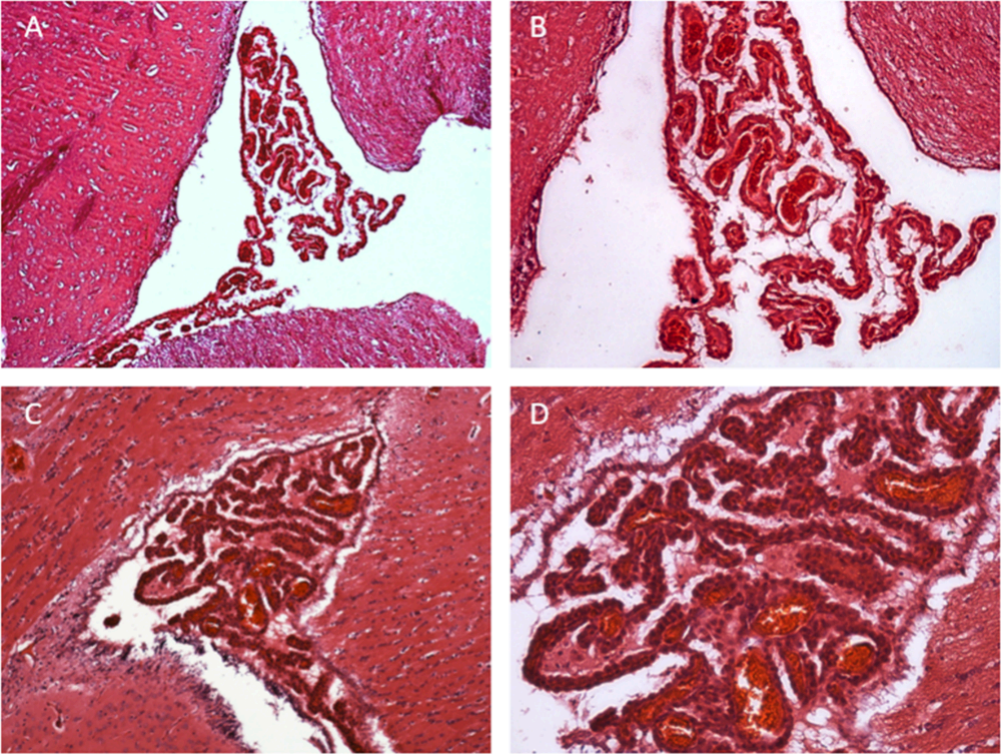
In the left panel, representative choroid plexi histology (hematoxylin and eosin staining, 10× magnification (A, C) and 40× magnification (B, D) of a rat treated with vehicle (A, B) or with compound **4e** (C, D).

## Conclusions

In this study, we report the design, synthesis, and evaluation of a novel series of fluorinated reversible *N*-alkyl carbamate derivatives of psilocin. Structural diversification of the carbamate promoiety through systematic fluorination enabled fine control over carbamate bond stability without compromising molecular properties relevant to oral absorption and brain distribution. Density Functional Theory (DFT) calculations supported the experimental findings, showing that increasing fluorination enhances the acidity of the carbamate NH proton and correlates with the observed hydrolysis trends under physiological conditions. To the best of our knowledge, this study represents the first systematic effort to fine-tune derivative stability within a consistent carbamate scaffold by varying fluorine content and positioning on the promoiety, thereby offering a potentially generalizable strategy for CNS-targeted agents.

As part of the synthetic workflow, we introduced key optimizations to established procedures for psilocin synthesis from 4-(benzyloxy)-1*H*-indole and for psilocybin phosphorylation using tetrabenzylpyrophosphate. Specifically, we addressed a known issue of intramolecular *O*-to-*N* benzyl migration by developing a reproducible and scalable two-step, one-pot protocol, which delivered psilocybin in the highest reported yield (85%) with markedly improved simplicity and efficiency.

Pharmacokinetic and pharmacodynamic studies identified compound **4e** as a representative lead within the synthesized derivatives. While **4e** exhibited favorable oral bioavailability and efficient brain penetration, it underwent partial bioconversion to psilocin in competition with oxidative metabolic pathways. Moreover, functional assays demonstrated that **4e** retains intrinsic serotonergic activity at 5-HT_2A_ and 5-HT_2C_ receptors, indicating that it does not behave as a pharmacologically inert prodrug. Importantly, despite this intrinsic serotonergic activity, compound **4e** induced attenuated psychotropic effects in vivo relative to psilocybin, consistent with its reduced acute psilocin exposure and altered pharmacokinetic profile. Taken together, these findings suggest that the behavioral outcome of **4e** reflects a context-dependent serotonergic signaling profile arising from the combined effects of intrinsic receptor activity and modified exposure kinetics, an aspect that warrants further investigation.

Collectively, these findings demonstrate that fluorinated carbamate chemistry provides a versatile platform for modulating psilocin exposure and serotonergic signaling, rather than a straightforward route to an ideal prodrug, offering a framework for the future design of serotonergic agents with improved pharmacological control and reduced acute psychoactive effects.

## Experimental Section

### General Chemistry Information

Reagent grade reagents and solvents purchased from commercial suppliers were used without additional purification. Reaction monitoring was performed by thin-layer chromatography (TLC) using plastic plates (40 × 80 mm) precoated with 200 μm silica gel (60 Å pore size, F_254_, Merck). Plates were revealed by 254 nm UV light irradiation and/or vanillin staining. Compound purification was performed by silica gel column chromatography (220–440 mesh, 60 Å pore size, Merck, positive nitrogen pressure), or by reverse phase semipreparative HPLC (Agilent 1260 Infinity II, column Phenomenex Luna C18, 5 μm, 100 Å, 250 × 21.2 mm, elution with water +0.1% TFA/ACN). ^1^H and ^13^C nuclear magnetic resonance (NMR) spectra were acquired on a Bruker Avance III HD 400 spectrometer operating at 400 MHz for ^1^H and 101 MHz for ^13^C. ^19^F NMR spectra were acquired on a Bruker Avance III HD 400 spectrometer operating at 376 MHz or a Bruker 200 spectrometer operating at 188 MHz. Chemical shifts (δ) are given in ppm and coupling constants (*J*) in Hz. MestReNova (v14.2.0-26256) software was used to process NMR spectra, which were calibrated with the residual solvent signal (^1^H and ^13^C) or with CFCl_3_ as reference (^19^F). Liquid chromatography/mass spectrometry (LC/MS) was performed on an Agilent 6550 IFunnel Q-TOF with UPLC 1290 Infinity LC/MS system. The purity of the final compounds was assessed by UPLC analysis on an Agilent 1290 Infinity system equipped with DAD detector and a ZORBAX Eclipse XDB-C18 (2.1 × 50 mm, 1.8 μm) column at 25 °C, injecting a 0.1 to 0.5 mM solution in water/acetonitrile or DMSO. Mobile phase A was water +0.1% TFA, while B was ACN + 0.1% TFA. Gradient from 5% B, reaching 100% B in 10 min (for compounds **4a–e** and **PSI**) or initial 1.5 min isocratic 2% B, then reaching 100% B in 10 min (for **PSY**). Chemical, metabolic, and plasma stability analyses were performed using an UPLC method with an isocratic hold at 2% solvent B for 1.5 min, followed by a linear gradient to 52.5% B over 5.3 min. Detection was carried out at 254 nm, with an injection volume of 5 μL. All tested compounds were >95% pure by UPLC analysis.

#### 2-(4-(Benzyloxy)-1*H*-indol-3-yl)-*N*,*N*-dimethyl-2-oxoacetamide (**1**)

A solution of oxalyl chloride (3.07 mL, 35.8 mmol, 2.0 equiv) in anhydrous diethyl ether (35 mL) was added dropwise over 10 min under nitrogen atmosphere to an ice-cooled solution of 4-(benzyloxy)-1*H*-indole (**BOI**, 4.00 g, 17.9 mmol, 1.0 equiv) in anhydrous diethyl ether (45 mL). The reaction mixture was stirred at 0 °C for 1 h, during which time the evolved HCl gas was continuously purged with a gentle nitrogen flow. When TLC showed the disappearance of the starting material, dimethylamine (2 M in THF) was added dropwise to the mixture until the solution turned basic (this required about 45 mL of dimethylamine, 89.5 mmol, 5.0 equiv). The reaction was stirred for 30 min, until no chloride intermediate was detectable by TLC. Then, the reaction mixture was diluted in EA (300 mL) and washed with a 1:1 solution of brine and saturated NaHCO_3_ (300 mL). The aqueous phase was extracted 5 times with 100 mL of EA. All the organic fractions were combined, dried over Na_2_SO_4_ and then concentrated to dryness. The crude product was purified via flash chromatography using DCM/acetone 8:2 as eluent system, obtaining **1** as a pale-yellow solid (4.780 g, 14.8 mmol, 83% yield). HRMS (ESI): *m*/*z* calculated for C_19_H_18_N_2_O_3_ + H^+^ [M + H^+^]: 323.1390 Found: 323.1375. ^1^H NMR (400 MHz, CDCl_3_) δ 10.23 (s, 1H), 7.54–7.48 (m, 3H), 7.40–7.33 (m, 2H), 7.32–7.26 (m, 1H), 7.02 (t, *J* = 8.0 Hz, 1H), 6.89 (dd, *J* = 8.2, 0.7 Hz, 1H), 6.62 (dd, *J* = 8.0, 0.7 Hz, 1H), 5.23 (s, 2H), 2.94 (s, 3H), 2.89 (s, 3H). ^13^C NMR (101 MHz, CDCl_3_) δ 186.67, 169.34, 152.92, 138.99, 137.35, 134.79, 128.71, 127.87, 127.28, 124.64, 115.07, 114.85, 106.05, 105.07, 70.79, 37.53, 34.32.

#### 2-(4-(Benzyloxy)-1*H*-indol-3-yl)-*N*,*N*-dimethylethan-1-amine (**2**)

To an ice-cooled solution of **1** (4.780 g, 14.8 mmol, 1.0 equiv) in anhydrous THF (70 mL), stirred under nitrogen atmosphere, was dropwise added LiAlH_4_ (1 M in THF, 30.0 mL, 30.0 mmol, 2.0 equiv). The mixture was stirred at room temperature for 1 h. After that, an additional portion of LiAlH_4_ (44.0 mL, 44.0 mmol, 3.0 equiv) was added, and the mixture was heated at reflux for 16 h. The reaction was cooled to 0 °C and slowly quenched by dropwise adding a solution of saturated Rochelle’s salt, until no more bubbling was detected. The suspension was then filtered through a Celite pad and the insoluble material was washed with EA. The organic phase was dried over Na_2_SO_4_ and evaporated under reduced pressure. The crude product was purified by column chromatography using CHCl_3_/MeOH 95:5 + 1% TEA to 9:1 + 1% TEA obtaining **2** as a brown solid (3.862 g, 13.1 mmol, 88% yield). HRMS (ESI): *m*/*z* calculated for C_19_H_22_N_2_O + H^+^ [M + H^+^]: 295.1805 Found: 295.1803. ^1^H NMR (400 MHz, CDCl_3_) δ 8.03 (s, 1H), 7.5–7.48 (m, 2H), 7.42–7.36 (m, 2H), 7.36–7.31 (m, 1H), 7.07 (t, *J* = 7.9 Hz, 1H), 6.98 (d, *J* = 7.7 Hz, 1H), 6.92 (d, *J* = 1.7 Hz, 1H), 6.57 (d, *J* = 7.7 Hz, 1H), 5.18 (s, 2H), 3.12–3.06 (m, 2H), 2.73–2.66 (m, 2H), 2.17 (s, 6H). ^13^C NMR (101 MHz, CDCl_3_) δ 153.90, 138.33, 137.55, 128.62, 127.95, 127.93, 122.67, 120.86, 117.52, 114.64, 104.89, 100.42, 70.04, 61.50, 45.07, 25.00.

#### 3-(2-(Dimethylamino)­ethyl)-1*H*-indol-4-ol (**PSI**)

A 250 mL round-bottom flask was charged with Pd/C (10% loading, 0.082 g, 15% w/w) and purged with nitrogen. Then, a solution of **2** (0.600 g, 2.04 mmol, 1.0 equiv) in MeOH (28 mL) was added, followed by a solution of ammonium formate (0.579 g, 9.2 mmol, 5.4 equiv) in MeOH (28 mL). The solution was heated to reflux for 20 min, and then quickly cooled to 0 °C. Then, the suspension was filtered over a short Celite pad, which was washed with MeOH. The crude product was purified by silica gel column chromatography using CHCl_3_/MeOH 95:5 + 1% NH_3_ (7 M in MeOH) to 9:1 + 1% NH_3_, obtaining **PSI** as an off-white crystalline solid (0.387 g, 1.9 mmol, 93% yield). UPLC purity >99% (RT 1.4 min). HRMS (ESI): *m*/*z* calculated for C_12_H_16_N_2_O + H^+^ [M + H^+^]: 205.1335 found: 205.1338. ^1^H NMR (400 MHz, acetone-d6) δ 12.21 (s, 1H), 9.76 (s, 1H), 6.94 (d, *J* = 2.3 Hz, 1H), 6.87 (t, *J* = 7.7 Hz, 1H), 6.80 (dd, *J* = 8.1, 1.1 Hz, 1H), 6.34 (dd, *J* = 7.5, 1.1 Hz, 1H), 2.95–2.89 (m, 2H), 2.69–2.63 (m, 2H), 2.31 (s, 6H). ^13^C NMR (101 MHz, acetone-d6) δ 153.29, 140.46, 123.32, 122.19, 118.53, 114.71, 106.10, 103.29, 62.70, 45.61, 29.84, 25.97.

#### 3-(2-(Dimethylamino)­ethyl)-1*H*-indol-4-yl Dihydrogen Phosphate (PSY)

A solution of **PSI** (0.100 g, 0.49 mmol, 1.0 equiv) in anhydrous THF (10.0 mL), maintained under nitrogen atmosphere, was cooled to −78 °C and LDA (2 M in THF/heptane/diethylbenzene, 0.304 mL, 0.61 mmol, 1.24 equiv) was added dropwise. After 10 min at −78 °C, tetrabenzyl pyrophosphate (TBPP, 0.328 g, 0.61 mmol, 1.24 equiv) was added, the solution was allowed to warm to −10 °C and stirred for 2 h. At completion, the reaction was quenched with 30 mL of saturated aq. NH_4_Cl, and extracted with EA (4 × 30 mL). The organic fractions were collected, dehydrated over Na_2_SO_4_ and evaporated under reduced pressure. The obtained oil was dissolved in MeOH (10.0 mL) and added to Pd/C (10% loading, 0.010 g, 10% w/w), maintained under inert atmosphere. The flask was purged with hydrogen gas and stirred for 2 h at rt. After that, the catalyst was removed by filtration through a short Celite pad, which was washed with MeOH. The solution was evaporated under reduced pressure, the residue dissolved in water/ACN 95:5 and purified via RP semiprep HPLC (ACN gradient 3%/min, RT 5.6 min). After freeze-drying, **PSY** was obtained as a fluffy white solid (0.119 g, 0.42 mmol, 85% yield). UPLC purity >99% (RT 1.04 min, isocratic 2% ACN). HRMS (ESI): *m*/*z* calculated for C_12_H_17_N_2_O_4_P + H^+^ [M + H^+^]: 285.0999 Found: 285.1004. ^1^H NMR (400 MHz, D_2_O) δ 7.22 (dt, *J* = 8.2, 0.8 Hz, 1H), 7.15–7.09 (m, 2H), 6.99 (dt, *J* = 7.9, 1.0 Hz, 1H), 3.37–3.31 (m, 2H), 3.24–3.17 (m, 2H), 2.82 (s, 6H). ^13^C NMR (101 MHz, D_2_O) δ 145.67 (d, *J* = 6.8 Hz), 138.68, 124.21, 122.66, 118.42 (d, *J* = 6.7 Hz), 108.93 (d, *J* = 2.9 Hz), 107.99, 107.82, 59.01, 42.78, 21.68.

#### General Procedure for the Synthesis of 4-Nitrophenyl Activated Carbamates (**3a–e**)

To an ice-cooled suspension of amine hydrochloride (1.0 equiv) and BNPC (1.0 equiv) in DCM (2.0 mL/mmol), a solution of DMAP (2.0 equiv) in DCM (1 mL/mmol) was added dropwise. The solution was stirred at 0 °C for 1 h. At completion, the solution was partitioned between DCM and 0.5 M aq. HCl. The aqueous layer was further extracted 2 times with DCM. The organic fractions were collected, dehydrated over Na_2_SO_4_, evaporated under reduced pressure and the crude product was purified by column chromatography using DCM as eluent system.

#### 4-Nitrophenyl Isobutylcarbamate (**3a**)

Obtained from isobutylamine hydrochloride (0.500 g, 6.8 mmol), as a fluffy white solid (1.392 g, 5.88 mmol, 86% yield). HRMS (ESI): *m*/*z* calculated for C_11_H_14_N_2_O_4_ + H^+^ [M + H^+^]: 239.1026 found: 239.1023. ^1^H NMR (400 MHz, CDCl_3_) δ 8.24 (d, *J* = 9.1 Hz, 2H), 7.32 (d, *J* = 9.2 Hz, 2H), 5.17 (s, 1H), 3.12 (t, *J* = 6.5 Hz, 2H), 1.85 (dh, *J* = 13.4, 6.7 Hz, 1H), 1.03–0.93 (m, 6H). ^13^C NMR (101 MHz, CDCl_3_) δ 156.18, 153.38, 144.80, 125.22, 122.03, 48.85, 28.78, 20.02.

#### 4-Nitrophenyl­(3,3,3-trifluoropropyl)­carbamate (**3b**)

Obtained from 2,2,2-trifluoropropylamine hydrochloride (0.100 g, 0.67 mmol), as a fluffy white solid (0.133 g, 0.48 mmol, 72% yield). HRMS (ESI): *m*/*z* calculated for C_10_H_9_F_3_N_2_O_4_ + H^+^ [M + H^+^]: 279.0587 found: 279.0589. ^1^H NMR (400 MHz, CDCl_3_) δ 8.31–8.19 (m, 2H), 7.37–7.27 (m, 2H), 5.53–5.34 (m, 1H), 3.57 (q, *J* = 6.4 Hz, 2H), 2.45 (qt, *J* = 10.6, 6.5 Hz, 2H). ^13^C NMR (101 MHz, CDCl_3_) δ 155.73, 153.19, 145.10, 126.37 (q, *J* = 277.4 Hz), 125.31, 122.11, 35.06 (q, *J* = 4.1 Hz), 33.94 (q, *J* = 27.9 Hz).

#### 4-Nitrophenyl­(2-fluoroethyl)­carbamate (**3c**)

Obtained from 2-fluoroethylamine hydrochloride (0.500 g, 5.02 mmol), as a fluffy white solid (0.800 g, 3.51 mmol, 70% yield). HRMS (ESI): *m*/*z* calculated for C_9_H_9_FN_2_O_4_ + H^+^ [M + H^+^]: 229.0619 found: 229.0615. ^1^H NMR (400 MHz, CDCl_3_) δ 8.30–8.20 (m, 2H), 7.37–7.28 (m, 2H), 5.50 (s, 1H), 4.67–4.48 (m, 2H), 3.68–3.53 (m, 2H). ^13^C NMR (101 MHz, CDCl_3_) δ 155.83, 153.33, 145.06, 125.31, 122.14, 82.43 (d, *J* = 167.7 Hz), 41.94 (d, *J* = 19.8 Hz).

#### 4-Nitrophenyl­(2,2-difluoroethyl)­carbamate (**3d**)

Obtained from 2,2-difluoroethylamine hydrochloride (0.500 g, 5.02 mmol), as a fluffy white solid (0.976 g, 3.96 mmol, 93% yield). HRMS (ESI): *m*/*z* calculated for C_9_H_8_F_2_N_2_O_4_ + H^+^ [M + H^+^]: 247.0525 found: 247.0523. ^1^H NMR (400 MHz, CDCl_3_) δ 8.30–8.21 (m, 2H), 7.38–7.29 (m, 2H), 6.12–5.72 (m, 1H), 5.43 (s, 1H), 3.75–3.60 (m, 2H). ^13^C NMR (101 MHz, CDCl_3_) δ 155.56, 153.44, 145.24, 125.35, 122.13, 113.41 (t, *J* = 241.6 Hz), 43.62 (t, *J* = 26.4 Hz).

#### 4-Nitrophenyl­(2,2,2-trifluoroethyl)­carbamate (**3e**)

Obtained from 2,2,2-trifluoroethylamine hydrochloride (1.000 g, 7.38 mmol), as a fluffy white solid (1.687 g, 6.40 mmol, 86% yield). HRMS (ESI): *m*/*z* calculated for C_9_H_7_F_3_N_2_O_4_ + H^+^ [M + H^+^]: 265.0431 found: 265.0430. ^1^H NMR (400 MHz, acetone-d6) δ 8.35–8.26 (m, 2H), 7.83 (s, 0H), 7.52–7.43 (m, 1H), 4.10–3.97 (m, 1H). ^13^C NMR (101 MHz, acetone-d6) δ 206.18, 156.93, 156.92, 154.62, 154.62, 145.97, 125.92, 125.63 (q, *J* = 278.1 Hz), 123.24, 116.57, 43.26 (q, *J* = 35.0 Hz).

#### General Procedure for the Synthesis of 4-*O*-(*N*-Alkyl carbamate) PSI Derivatives (**4a–e**)

To an ice-cooled solution of **PSI** (1.0 equiv) and **3** (2.0 equiv) in THF (7 mL/mmol) was added dropwise a solution of DMAP (1.2 equiv) in THF (3 mL/mmol). The solution was allowed to warm to room temperature and stirred for 16 h. At completion, most of the solvent was evaporated under reduced pressure and the residue was partitioned between DCM and sat. aq. NH_4_Cl/brine (3:2, until pH 7–8). The aqueous phase was extracted 3 more times, until all the product was recovered. The collected organic fractions were dehydrated over Na_2_SO_4_ and evaporated under reduced pressure. The crude product was purified by column chromatography using DCM/MeOH 97:3 to DCM/MeOH 9:1 + 1% NH_3_ 7 M in MeOH. The product was further purified via RP semiprep HPLC (ACN gradient 3.3%/min). Finally, the TFA^–^ counterion was exchanged with Cl^–^ eluting a 30% ACN solution of the compound through Amberlite IRA 400, followed by freeze-drying.

#### 3-(2-(Dimethylamino)­ethyl)-1*H*-indol-4-yl Isobutylcarbamate Hydrochloride (**4a**)

Obtained from **3a** (0.467 g, 1.96 mmol) as a gray solid (0.260 g, 0.76 mmol, 78% yield). UPLC purity >99% (RT 2.9 min). HRMS (ESI): *m*/*z* calculated for C_17_H_25_N_3_O_2_ + H^+^ [M + H^+^]: 304.2020 found: 304.2022. ^1^H NMR (400 MHz, MeOD) δ 7.26 (dd, *J* = 8.2, 0.8 Hz, 1H), 7.24 (s, 1H), 7.11 (t, *J* = 7.9 Hz, 1H), 6.74 (dd, *J* = 7.6, 0.9 Hz, 1H), 3.49–3.41 (m, 2H), 3.25–3.17 (m, 2H), 3.06 (d, *J* = 6.9 Hz, 2H), 2.90 (s, 6H), 1.86 (hept, *J* = 6.7 Hz, 1H), 0.99 (d, *J* = 6.7 Hz, 6H). ^13^C NMR (101 MHz, MeOD) δ 157.92, 145.47, 140.70, 125.57, 123.08, 121.15, 113.30, 110.36, 108.39, 60.03, 49.75, 43.61, 30.03, 23.03, 20.46.

#### 3-(2-(Dimethylamino)­ethyl)-1*H*-indol-4-yl­(3,3,3-trifluoropropyl)­carbamate Hydrochloride (**4b**)

Obtained from **3b** (0.133 g, 0.48 mmol) as a white solid (0.079 g, 0.21 mmol, 87% yield). UPLC purity >99% (RT 2.8 min). HRMS (ESI): *m*/*z* calculated for C_16_H_20_F_3_N_3_O_2_ + H^+^ [M + H^+^]: 344.1580 found: 344.1581. ^1^H NMR (400 MHz, acetone-d6) δ 12.43 (s, 1H), 10.26 (s, 1H), 8.60 (s, 1H), 7.24–7.17 (m, 2H), 7.15–7.02 (m, 2H), 3.53–3.35 (m, 4H), 3.35–3.20 (m, 2H), 2.90 (s, 3H), 2.89 (s, 3H), 2.75–2.59 (m, 2H). ^13^C NMR (101 MHz, acetone-d6) δ 155.57, 145.53, 139.85, 129.02 (q, *J* = 276.0 Hz),124.73, 124.56, 122.50, 120.34, 112.94, 109.50, 59.71, 43.20, 35.31, 34.22 (q, *J* = 27.6 Hz) 22.76. ^19^F NMR (376 MHz, acetone-d6) δ −64.92.

#### 3-(2-(Dimethylamino)­ethyl)-1*H*-indol-4-yl­(2-fluoroethyl)­carbamate Hydrochloride (**4c**)

Obtained from **3c** (0.800 g, 3.51 mmol) as an off-white foam (0.510 g, 1.54 mmol, 88% yield). UPLC purity >99% (RT 1.9 min). HRMS (ESI): *m*/*z* calculated for C_15_H_20_FN_3_O_2_ + H^+^ [M + H^+^]: 294.1612 found: 294.1619. ^1^H NMR (400 MHz, MeOD) δ 7.27 (dd, *J* = 8.2, 0.8 Hz, 1H), 7.22 (s, 1H), 7.10 (t, *J* = 7.9 Hz, 1H), 6.76 (dd, *J* = 7.7, 0.8 Hz, 1H), 4.62 (t, *J* = 4.8 Hz, 1H), 4.50 (t, *J* = 4.8 Hz, 1H), 3.57 (t, *J* = 4.9 Hz, 1H), 3.50 (t, *J* = 4.9 Hz, 1H), 3.40 (dd, *J* = 8.6, 6.7 Hz, 2H), 3.19 (dd, *J* = 8.6, 6.8 Hz, 2H), 2.89 (s, 6H). ^13^C NMR (101 MHz, MeOD) δ 157.78, 145.24, 140.64, 125.58, 123.03, 121.13, 113.34, 110.49, 108.45, 83.44 (d, *J* = 167.0 Hz), 60.05, 43.57, 42.80 (d, *J* = 20.3 Hz), 22.99. ^19^F NMR (188 MHz, MeOD) δ −223.87 (tt, *J* = 47.8, 27.1 Hz).

#### 3-(2-(Dimethylamino)­ethyl)-1*H*-indol-4-yl­(2,2-difluoroethyl)­carbamate Hydrochloride (**4d**)

Obtained from **3d** (0.771 g, 3.13 mmol) as an off-white foam (0.476 g, 1.37 mmol, 87% yield). UPLC purity >99% (RT 2.2 min). HRMS (ESI): *m*/*z* calculated for C_15_H_19_F_2_N_3_O_2_ + H^+^ [M + H^+^]: 312.1518 found: 312.1514. ^1^H NMR (400 MHz, MeOD) δ 7.28 (d, *J* = 8.1 Hz, 1H), 7.12 (s, 1H), 7.07 (t, *J* = 7.9 Hz, 1H), 6.75 (d, *J* = 7.6 Hz, 1H), 6.20–5.87 (m, 1H), 3.61 (td, *J* = 15.7, 3.5 Hz, 2H), 3.30–3.20 (m, 2H), 3.14–3.05 (m, 2H), 2.77 (s, 6H). ^13^C NMR (101 MHz, MeOD) δ 157.71, 145.03, 140.33, 125.56, 122.90, 120.92, 115.69 (t, *J* = 240.1 Hz), 113.24, 110.62, 108.46, 59.73, 44.19 (t, *J* = 25.5 Hz), 43.42, 22.79. ^19^F NMR (188 MHz, MeOD) δ −123.33 (dt, *J* = 55.8, 15.6 Hz).

#### 3-(2-(Dimethylamino)­ethyl)-1*H*-indol-4-yl­(2,2,2-trifluoroethyl)­carbamate Hydrochloride (**4e**)

Obtained from **3e** (0.793 g, 3.0 mmol) as an off-white solid (0.479 g, 1.31 mmol, 87% yield). UPLC purity >99% (RT 2.6 min). HRMS (ESI): *m*/*z* calculated for C_15_H_18_F_3_N_3_O_2_ + H^+^ [M + H^+^]: 330.1424 found: 330.1426. ^1^H NMR (400 MHz, acetone-d6) δ 11.67 (s, 1H), 10.35 (s, 1H), 8.20 (t, *J* = 6.6 Hz, 1H), 7.30–7.23 (m, 2H), 7.10 (t, *J* = 7.9 Hz, 1H), 6.96 (d, *J* = 7.7 Hz, 1H), 4.09–3.93 (m, 2H), 3.47–3.37 (m, 2H), 3.37–3.29 (m, 2H), 3.01 (s, 3H), 3.00 (s, 3H). ^13^C NMR (101 MHz, Acetone-d6) δ 161.89, 155.99, 145.27, 139.90, 127.27 (q, *J* = 280.3 Hz), 124.50, 122.52, 120.32, 112.94, 109.89, 109.31, 59.64, 43.24 (q, *J* = 34.7 Hz), 43.16, 22.67. ^19^F NMR (377 MHz, acetone) δ −72.17.

### DFT Calculations

All calculations were performed using the Amsterdam Density Functional (ADF) software package.^75,76^ The GGA exchange–correlation functional BLYP was used for the optimizations of all stationary points.^77,78^ The exchange–correlation functional was used in combination with the TZ2P basis set (triple-ζ quality augmented with two sets of polarization functions on each atom). Grimme dispersion was included.^79–81^ This level of theory is denoted in the text as BLYP-D3­(BJ)/TZ2P and was found to perform well in other studies involving organic molecules and their reactivity.^82,83^

### Chemical Stability

Stability of the synthesized compounds was tested in aqueous media at physiologically relevant pH values (1, 6.8, and 7.4) and in human plasma. 100 μM solutions of the compounds in HCl 0.1 M or PBS (0.1 M phosphate buffer, pH 6.8 or 7.4) were incubated at 37 °C for 24 h, and analyzed by HPLC-UV at different times (0, 2, 4, 6, 8, 12, 16, 20, 24 h). In the case of plasma stability, plasma was spiked with the compound (300 μM final concentration), and incubated at 37 °C for 6 h; 100 μL aliquots were withdrawn at different time points (15 and 30 min; 1, 2, 4, and 6 h), mixed with 400 μL of MeOH, centrifuged (10,000 × *g*, 7 min), and analyzed by UPLC/UV. **PSY** samples were further diluted 1:3 with water prior to injection to minimize peak tailing. The hydrolysis reaction rate constants (*k*) of the **PSI** derivatives were calculated through interpolation of experimental data with the equation for pseudo-first order reactions: [*C*] = [*C*]_0_ × e^–*kt*^, where [*C*]: concentration of the compound; [*C*]_0_: concentration of the compound at the initial time *t*_0_; *t*: time. Nonlinear curve fitting was performed using Origin 8.0 software.

### In Vitro Metabolic Stability

To evaluate the in vitro metabolic stability of compounds **4a–e**, human liver microsomal and S9 fractions were used, as previously described.^84^ Human pooled liver microsomes (HLMs) and S9 fractions were purchased from Sigma-Aldrich (St. Louis, MO, USA). Each **PSI** derivative was dissolved in DMSO at a concentration of 20 mM. For the assay, 2.5 μM of each compound was incubated at 37 °C for 10, 20, 30, 60, 120, 240, 360 min in 0.1 M phosphate buffer (pH 7.4) containing 50 μg of microsomal or S9 protein and 10 mM NADPH, with the addition of 50 μL NADPH regeneration system (starting solution: 5.2 mM NAD+, 13.2 glucose-6-phosphate, 1.6 UI/mL glucose-6-phosphate dehydrogenase). At the end of the incubation, enzymatic activity was quenched by the addition of cold acetonitrile containing 2% formic acid, followed by centrifugation at 12,000 × *g* for 10 min. The resulting supernatants were collected and analyzed by means of UPLC-MS, to assess the formation of **PSI** and evaluate the metabolic stability of the tested compounds.

### Fluorometric Imaging Plate Reader (FLIPR)/Ca^2+^ Assay

The FLIPR Ca^2+^ mobilization assay was conducted at Aptuit, an Evotec Company (Italy), to characterize the agonist activity of the compounds at human 5-HT_2A_, 5-HT_2B_, and 5-HT_2C_ receptors expressed in CHO-K1 (Chinese Hamster Ovary) cells using a 384-well format, as previously described.^69^ Briefly, to assess their agonist activity, **PSI**, **5-HT**, and **4e** were tested for the ability to induce intracellular calcium mobilization at 5-HT_2A_, 5-HT_2B_, or 5-HT_2C_ receptors in recombinant 5-HT_2A_, 5-HT_2B_, and 5-HT_2C_ CHO-K1 cell lines using Cal-520, a no-wash calcium-sensitive dye. The response was measured in real time following receptor stimulation. Each compound was profiled in 11-point concentration–response curves, performed in duplicate across two independent experiments following a standardized protocol. Pharmacodynamic parameters (pEC_50_ and Emax) were determined by fitting the experimental data to a nonlinear regression model.

### Head-Twitch Response (HTR) Behavioral Assessment

Male C57BL/6 mice were randomized into four different groups (*n* = 5), treated by oral gavage either with vehicle (saline), 1 mg/kg **PSY**, 3 mg/kg **PSY**, or **4e** (**PSY** equiv dose). Mice were habituated in a transparent container for 10 min prior to treatment. Following administration, they were returned into the transparent container and their behavior was recorded for 45 min using a video camera positioned horizontally, to facilitate HTRs detection. HTRs were manually scored by two investigators (MC and AS), who were blinded to the experimental conditions. The total number and time-course of HTRs during the observation period were quantified. Data were analyzed by one-way ANOVA, followed by the post hoc Newman–Keuls test. A *p*-value <0.05 was considered statistically significant.

### Plasma and Brain Pharmacokinetics in C57BL/6J Mice

For the pharmacokinetic analysis, a single oral dose of 20 mg/kg of compound **4e** or **PSY** was administered by gavage. Blood samples (*n* = 3–6 per time point) were collected from the submandibular plexus at *T*_0_ before gavage and at 0.25, 0.5, 1, 2, 3, 4, 6, 24, and 48 h after compound administration. Mice brains were collected at each time point after sacrifice. Plasma was separated by centrifugation and stored at −80 °C until analysis. **4e** and **PSI** concentrations were determined by LC-MS/MS, and pharmacokinetic parameters were calculated using PKSolver, an add-in for Microsoft Excel, based on noncompartmental analysis. Data were analyzed using GraphPad Prism (version 10.2; GraphPad Software Inc., San Diego, CA, USA) and expressed as mean ± standard deviation (SD), unless otherwise specified. Statistical comparisons were performed using Student’s *t* test or one-way ANOVA, where appropriate. A *p*-value <0.05 was considered statistically significant.

### Toxicological Analysis in Sprague-Dawley Rats

Male Sprague-Dawley rats (body weight 200 ± 20 g) were treated with either vehicle (saline) or 100 mg/kg **4e** via gastric gavage, following anesthesia induced by inhalation of isoflurane (Vetflurane Inhalation Vapor, Liquid, Virbac). 24 h after the administration, the animals were suppressed, blood was collected via intracardiac puncture and placed in appropriate tubes for evaluating blood cell counts and plasma biochemistry (Padova University Hospital). The main organs (kidneys, liver, brain, heart, and lungs) were collected without prior perfusion, washed in PBS and placed in 10% formalin for tissue histology using standard techniques.^74^

### Pharmacokinetic LC-MS/MS Analyses

Psilocin (CAS No. 520536) and Psilocin-d_10_ as internal standard (CAS No. 1435934-64-7) were acquired from Merck Life Science (Germany). In order to assess the **PSI**, and compound **4e** concentration in plasma and whole brain homogenate, after their oral administration in mice, an LC-MS/MS analytical method was developed and validated in accordance with the guidelines of the US FDA.^85^ For each analyte the following parameters were evaluated: linearity, accuracy, precision, selectivity and sensitivity, matrix effect, extraction recovery and stability (see Supporting Information S22, Tables S3–S7, and Figures S3–S5). The LC separation was conducted with a high-performance liquid chromatography with an Accela 600 pump and an online degasser connected to a CTC automatic injector (Thermo Scientific, MA, USA). Chromatographic separation was achieved using an analytical column Hypersil Gold (50 × 2.1 mm, 1.9 μm, Thermo Scientific) using a mobile phase consisting of 0.1% formic acid in water (A) and 0.1% formic acid in methanol (MeOH) (B). Detection was performed using an LTQ XL ion trap mass spectrometer (Thermo Scientific, MA, USA) with an electrospray ionization source, in positive mode. The gradient used and all the analytical parameters are reported in Supporting Information S23 Table S1 and S2.

#### Plasma Extraction

To an aliquot of 50 μL of plasma was added ascorbic acid and psilocin-d_10_ internal standard (IS used for **PSI**) in order to achieve a final concentration of 1 mM and 25 ng/mL respectively. Then 150 μL of dichloromethane was added. The mixture was vortexed for 10 min and centrifuged at 16,060 × *g*, 4 °C for 10 min. The supernatant was eliminated, and the solvent was transferred in an Eppendorf tube and evaporated to dryness under a stream of air at 50 °C with a Turbovap evaporator (Zymark, Hopkinton MA, USA). The residue was dissolved in 50 μL of mobile phase (90:10%, v/v, 0.1% formic acid in water and 0.1% formic acid in MeOH) with ascorbic acid 1 mM. The mixture was vortexed and centrifuged at 16,060 × *g* for 10 min before transferring into the vial.

#### Brain Extraction

Whole-brain samples were thawed and weighed before being manually ground with 1 mL of deionized water using a small pestle. The brain homogenate was sonicated for 10 min and then vigorously vortexed. To an aliquot of 50 μL of brain homogenate sample, 5 μL Internal standard solution (250 ng/mL psilocin-d_10_), 5 μL ascorbic acid 0.040 M and 150 μL acetonitrile were added. The mixture was vortexed for 10 min, then centrifuged at 16,060 × *g* at 4 °C for 10 min. The organic supernatant was transferred in an Eppendorf tube and evaporated to complete dryness under a stream of air at 50 °C with a Turbovap evaporator (Zymark, Hopkinton MA, USA). The residue was dissolved in 50 μL of mobile phase (90:10%, v/v, 0.1% formic acid in water and 0.1% formic acid in MeOH) with ascorbic acid 1 mM. The mixture was vortexed and centrifuged at 16,060 × *g* for 10 min before transferring into the vial.

### Animal Housing and Ethics Statement

The animal study was approved by the Ethics Committee of the University of Padova for the welfare of laboratory animals (OPBA) and the Italian Ministry of Health (authorization n. 875/2023-PR-PR of 10/10/2023). All procedures involving animals were performed according to the institutional guidelines, complying with European Union Directive 2010/63/UE for experimental design and analysis in pharmacology care, the ARRIVE (Animal Research: Reporting of In Vivo Experiments) guidelines, and the 3R principle, to minimize animal pain and discomfort. Mice and rats were housed in individually ventilated cages (IVC) or in conditional cages, respectively, under controlled environmental conditions (12/12 h light/dark cycle, controlled temperature and humidity) with ad libitum access to food and water.

Human plasma used for the stability studies was obtained from one of the authors (SDM) as a volunteer, with informed consent. All procedures involving plasma handling were performed in accordance with standard laboratory safety practices.

Human liver microsomes (HLMs) and S9 fractions were purchased from the indicated commercial vendors as pooled, anonymized biological materials. All these samples were obtained in compliance with applicable ethical guidelines and did not require additional institutional review or informed consent.

## Supplementary Material



## Notes

^1^H-, ^13^C, ^19^F-NMR spectra, and UPLC chromatograms for purity determination of compounds **4a–e**; LC-MS/MS analytical method validation; experimental data on the chemical, plasma, and metabolic stability of **PSY** and compounds **4a–e**; concentration–response curves from the FLIPR calcium-flux assay of compound **4e**; preliminary qualitative identification of **4e**-derived metabolites; Cartesian coordinates and electronic energies of compounds **4a–e** and their corresponding anions; and histological evaluation of organ toxicity following administration of compound **4e** (PDF)
