# Supplementary material for: Design, Synthesis, and Pharmacokinetic Profiling of Fluorinated Reversible N‑Alkyl Carbamate Derivatives of Psilocin for Sub-Hallucinogenic Brain Exposure
Source: J Med Chem. 2026 Jan 26;69(3):2145–59. doi: 10.1021/acs.jmedchem.5c01797 (PMC12910641; doi:10.1021/acs.jmedchem.5c01797)
Supplement: Supplementary file 1 [file jm5c01797_si_001.pdf]

SUPPORTING INFORMATION

Design, Synthesis, and Pharmacokinetic Profiling of Fluorinated Reversible *N*-Alkyl Carbamate Derivatives of Psilocin for Sub-Hallucinogenic Brain Exposure

Marco Banzato[1], Martina Colognesi[1], Lorena Lucatello[2], Stefano Comai[1,3,4], Gianfranco Pasut[1], Francesca Capolongo[2], Laura Orian[5], Lucia Biasutto[6], Anna Signor[1], Daniela Gabbia[1], Paolo L. Manfredi[7,*], Sara De Martin[1,*], Andrea Mattarei[1,*]

1. *Department of Pharmaceutical and Pharmacological Sciences, University of Padova, Via Francesco Marzolo 5, 35131, Padua, Italy*
2. *Department of Comparative Biomedicine and Food Science, University of Padova, Viale dell'Università 16, Legnaro, 35020, Padua, Italy*
3. *Department of Biomedical Sciences, University of Padova, Via Ugo Bassi 58/B, 35131,. Padua, Italy*
4. *Department of Psychiatry, McGill University, 1033 Pine Avenue West, H3A 1A1 Montreal, QC, Canada.*
5. *Department of Chemical Sciences, University of Padova, Via Francesco Marzolo 1, 35131, Padua, Italy*
6. *Italian National Research Council (CNR) Neuroscience Institute, Viale Giuseppe Colombo 3, 35131, Padua, Italy*
7. *MGGM Therapeutics, 85 Baker Road, Kerhonkson, 12446, New York, United States*

Please address correspondence to Andrea Mattarei at andrea.mattarei@unipd.it or Paolo L. Manfredi at paolo.manfredi@mggmrx.com or Sara De Martin at sara.demartin@unipd.it

Table of contents

NMR spectra, UPLC chromatograms of the synthesized compounds S2

LC-MS/MS analytical method validation S22

Experimental data of metabolic stability of **PSY** and compounds **4a-e** in HLMs and S9 .... S32

Experimental data of chemical and plasma stability of **PSY** and compounds **4a-e** and determination of hydrolysis rate constants from experimental data S33

Concentration–response curves from the FLIPR calcium-flux assay of compound **4e** S41

Preliminary qualitative identification of **4e**-derived metabolites S42

Cartesian coordinates and electronic energies of compounds **4a-e** and the corresponding anions. Level of theory: BLYP-D3(BJ)TZ S44

Histological evaluation of organ toxicity following administration of compound **4e** S52

NMR spectra, UPLC chromatograms of the synthesized compounds

^{1}H and ^{13}C NMR spectra of **2-(4-(benzyloxy)-1*H*-indol-3-yl)-*N*,*N*-dimethyl-2-oxoacetamide (1)**

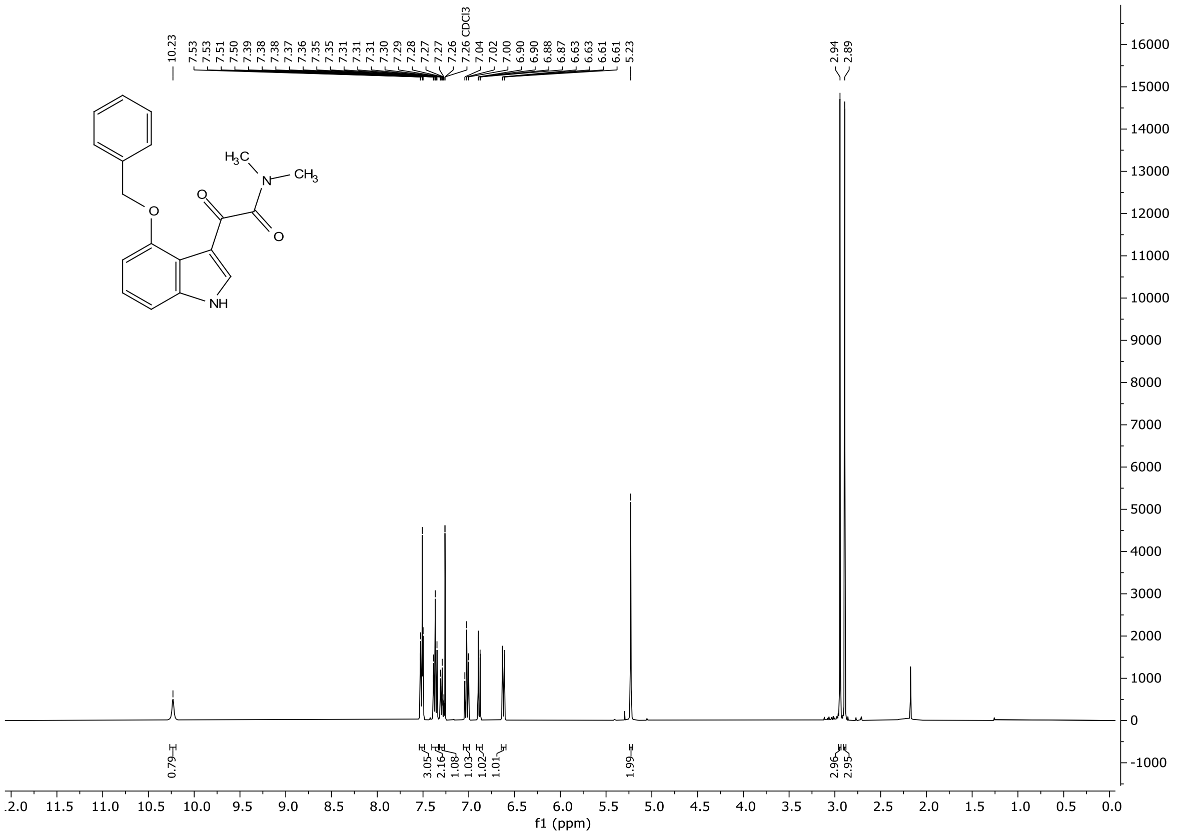

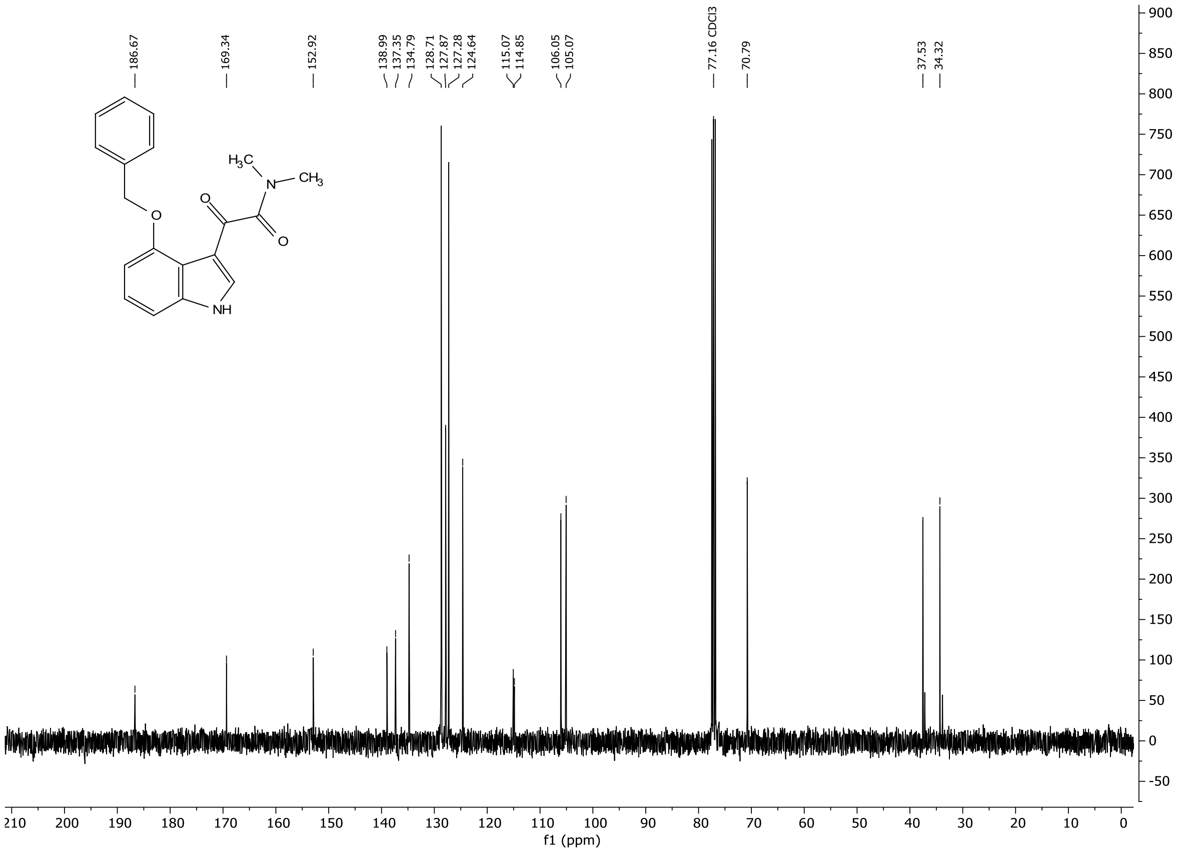

^{1}H and ^{13}C NMR spectra of **2-(4-(benzyloxy)-1*H*-indol-3-yl)-*N*,*N*-dimethylethan-1-amine (2)**

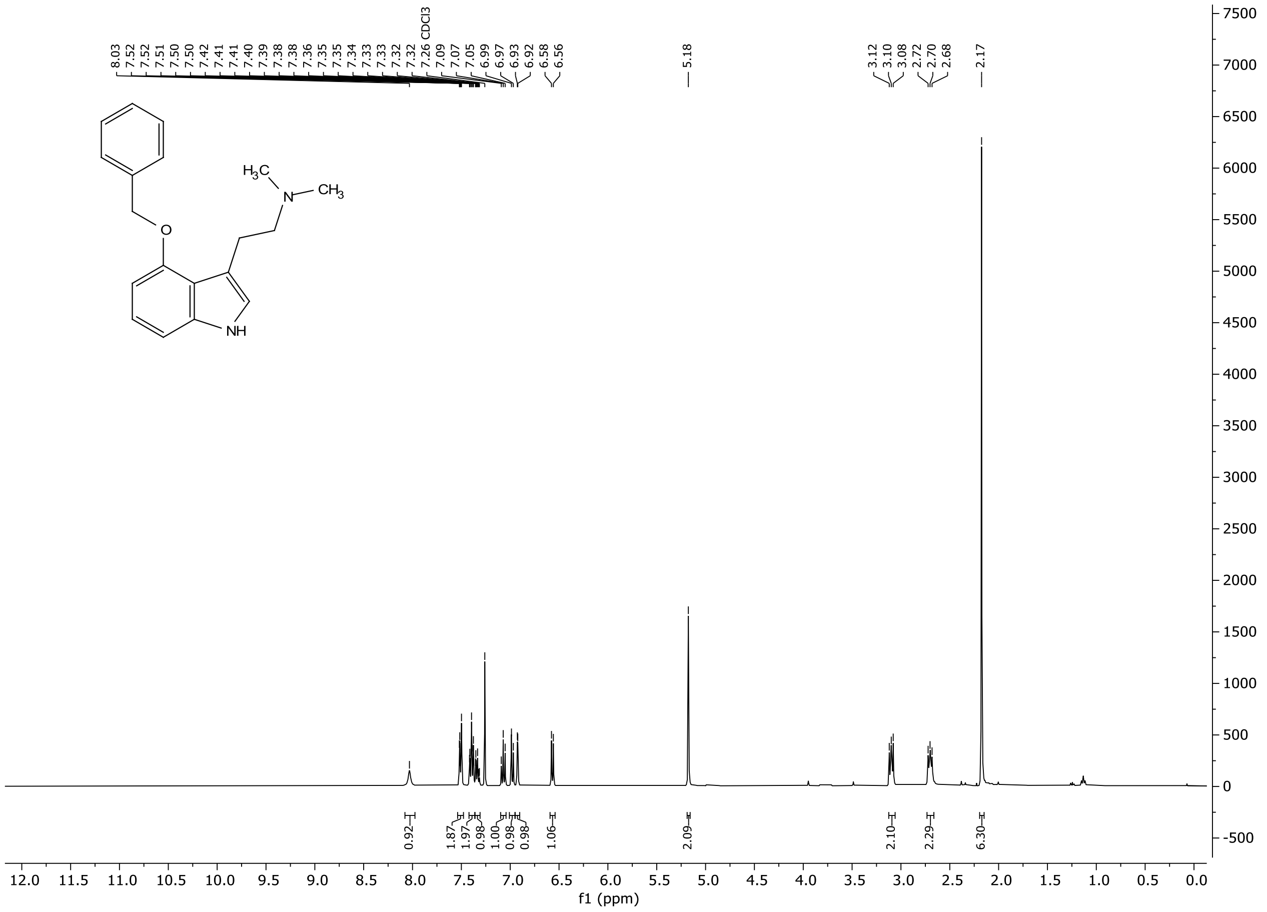

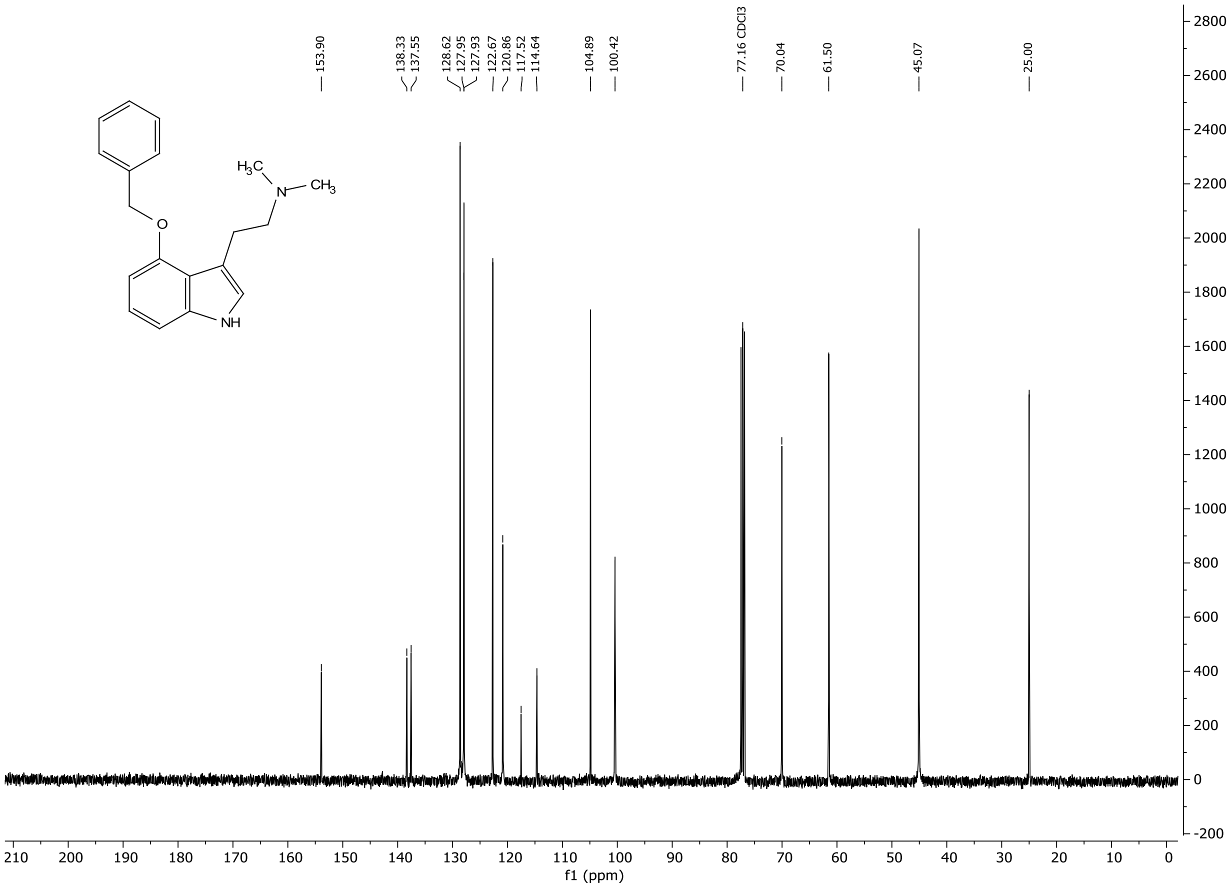

^{1}H, ^{13}C NMR spectra and UPLC chromatogram of **3-(2-(dimethylamino)ethyl)-1H-indol-4-ol (PSI)**

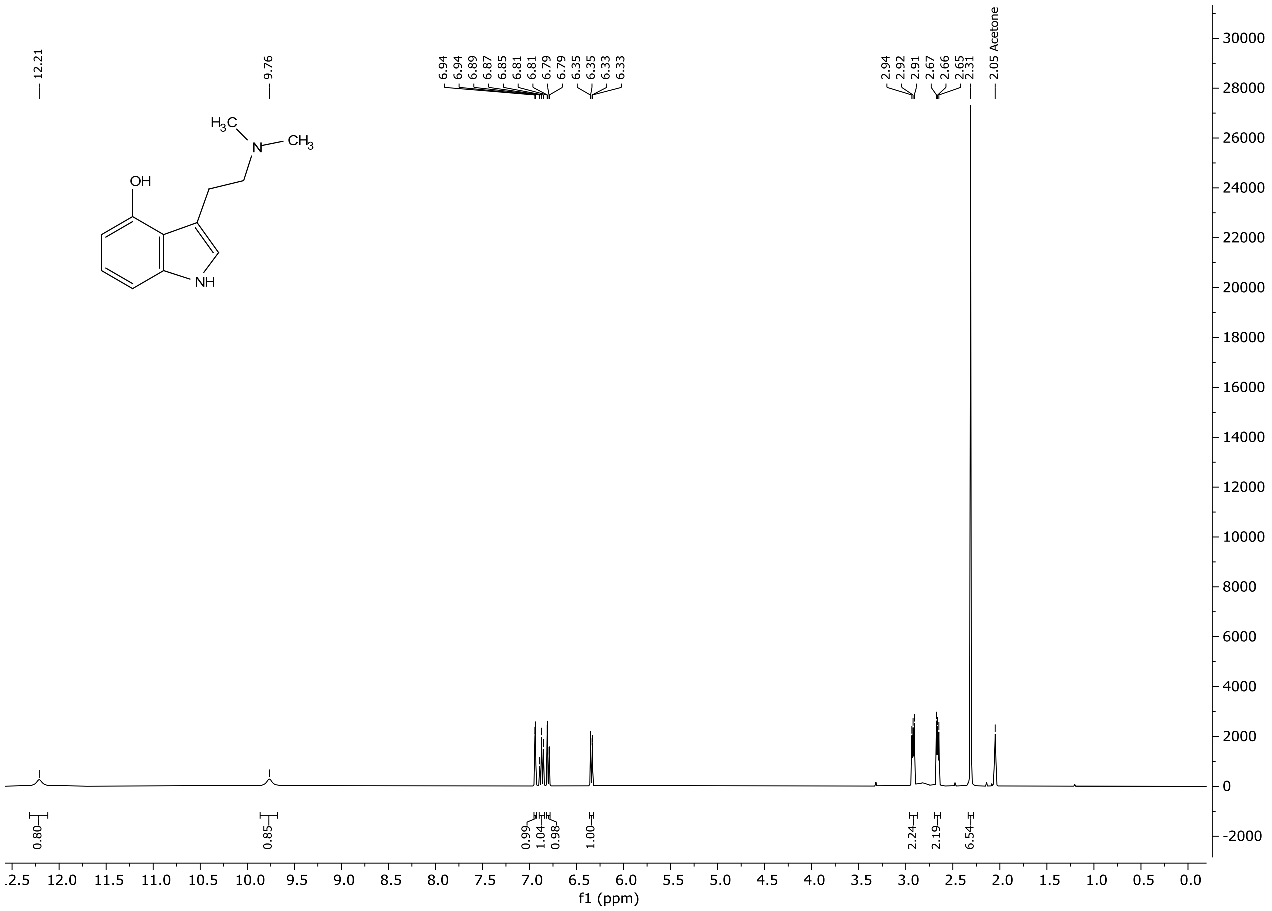

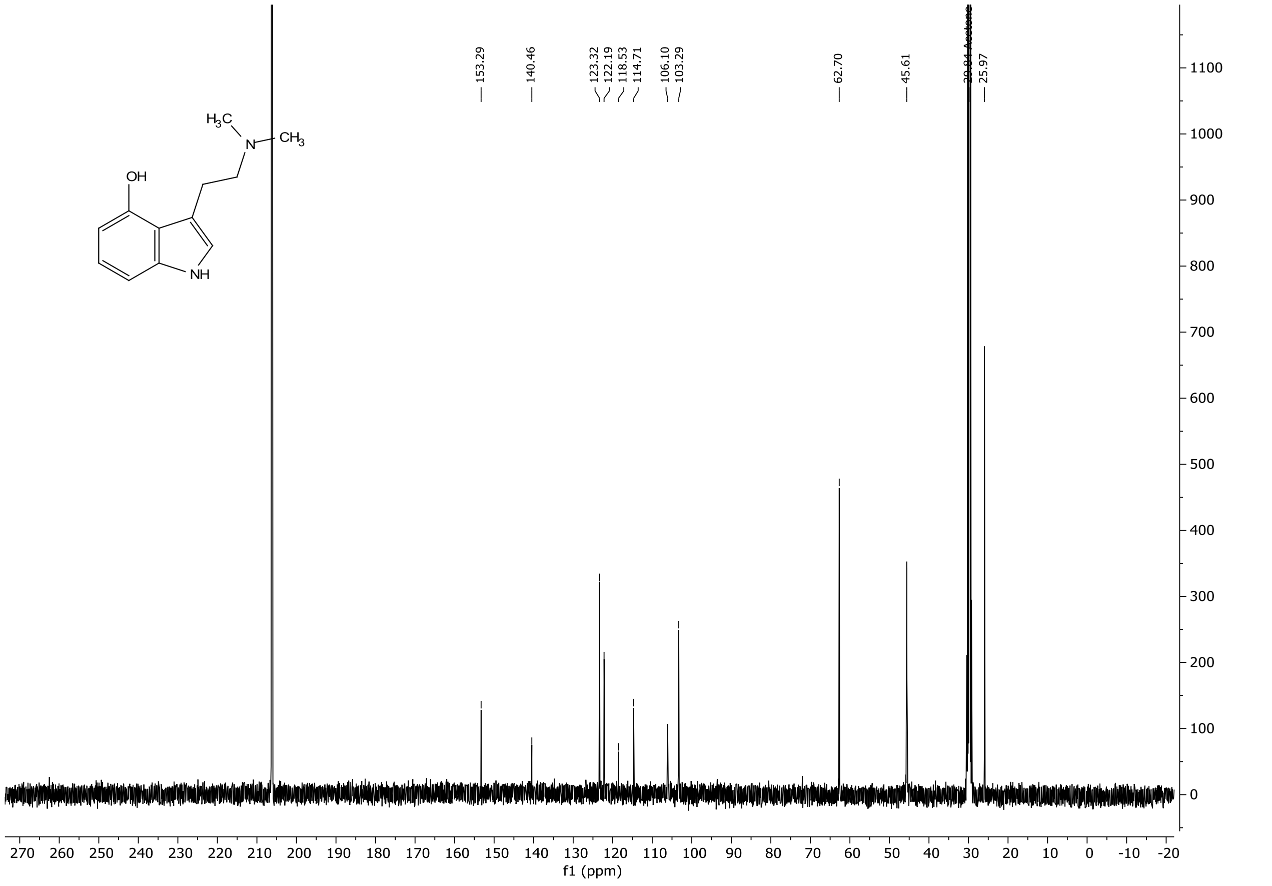

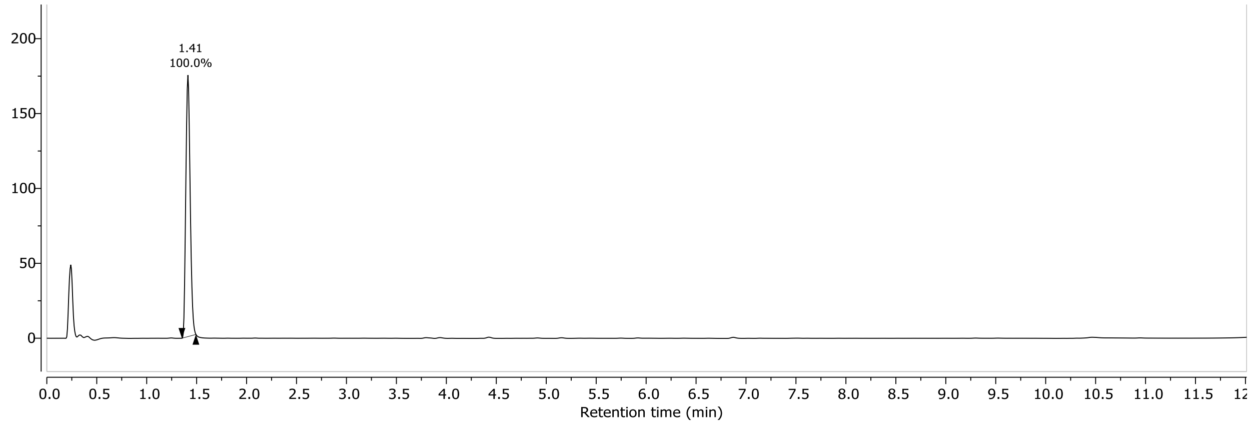

^{1}H, ^{13}C NMR spectra and UPLC chromatogram of **3-(2-(dimethylamino)ethyl)-1H-indol-4-yl dihydrogen phosphate (PSY)**

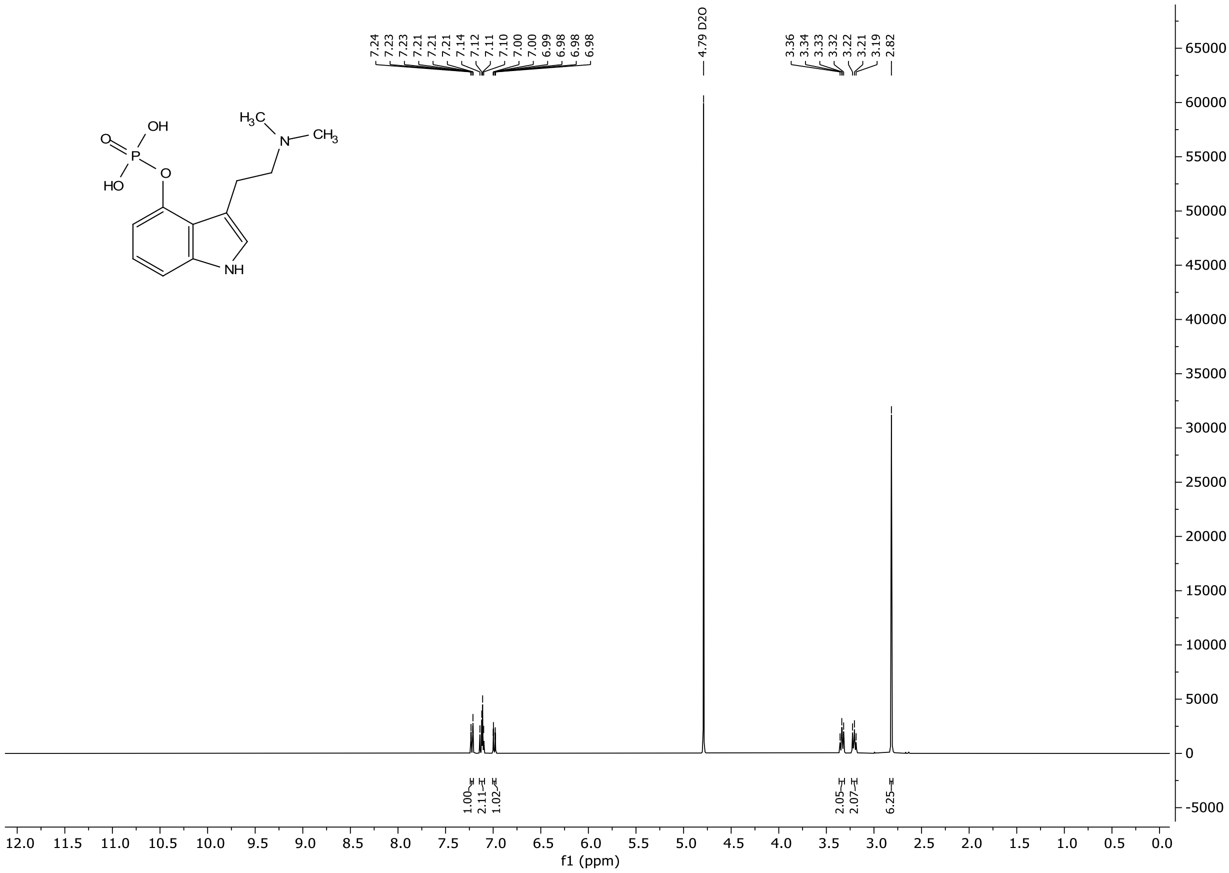

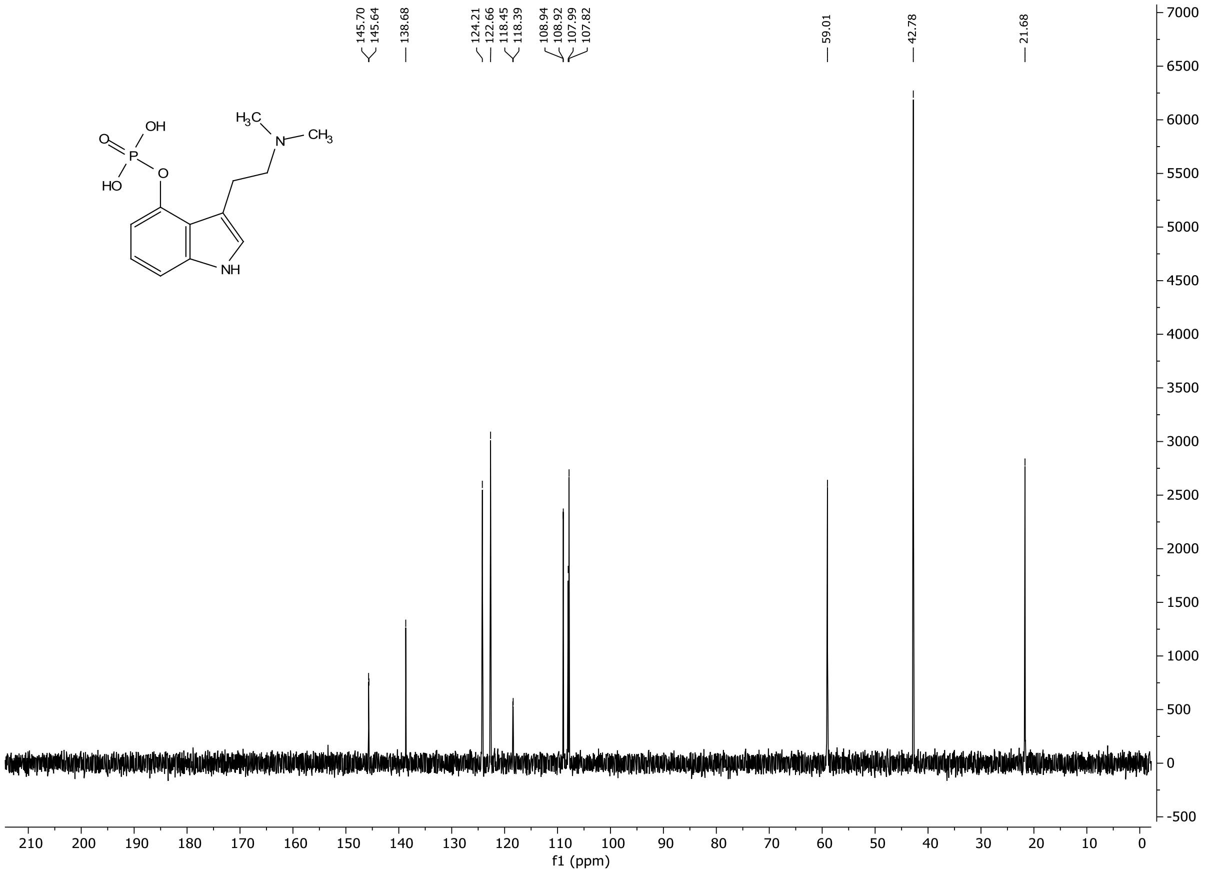
145.70
145.64
138.68
124.21
122.66
118.45
118.39
108.94
108.92
107.99
107.82
59.01
42.78
21.68
OH
O
P
HO
O
H_3C
N
CH_3
NH
f1 (ppm)

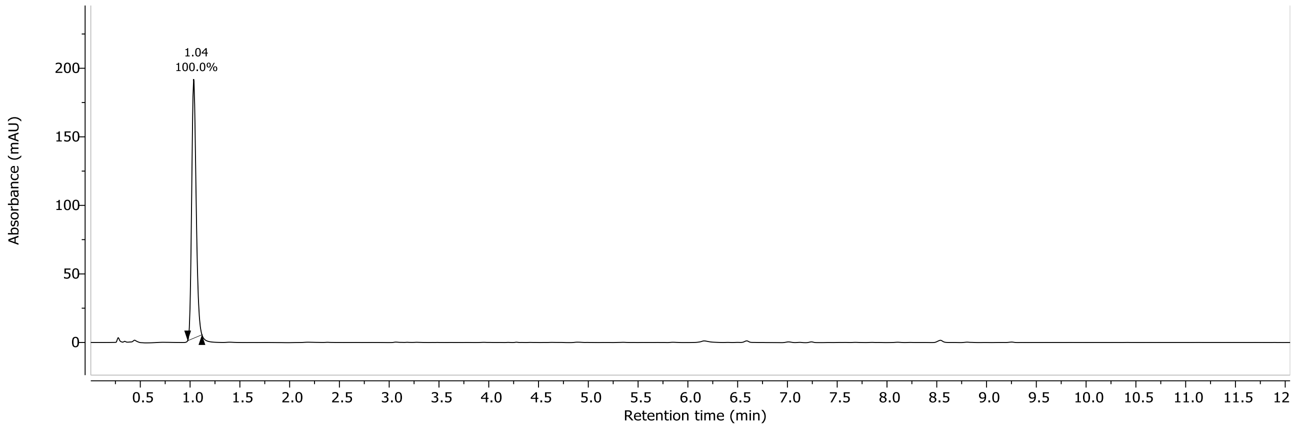
1.04
100.0%
Absorbance (mAU)
Retention time (min)

^{1}H and ^{13}C spectra of 4-nitrophenyl isobutylcarbamate (3a).

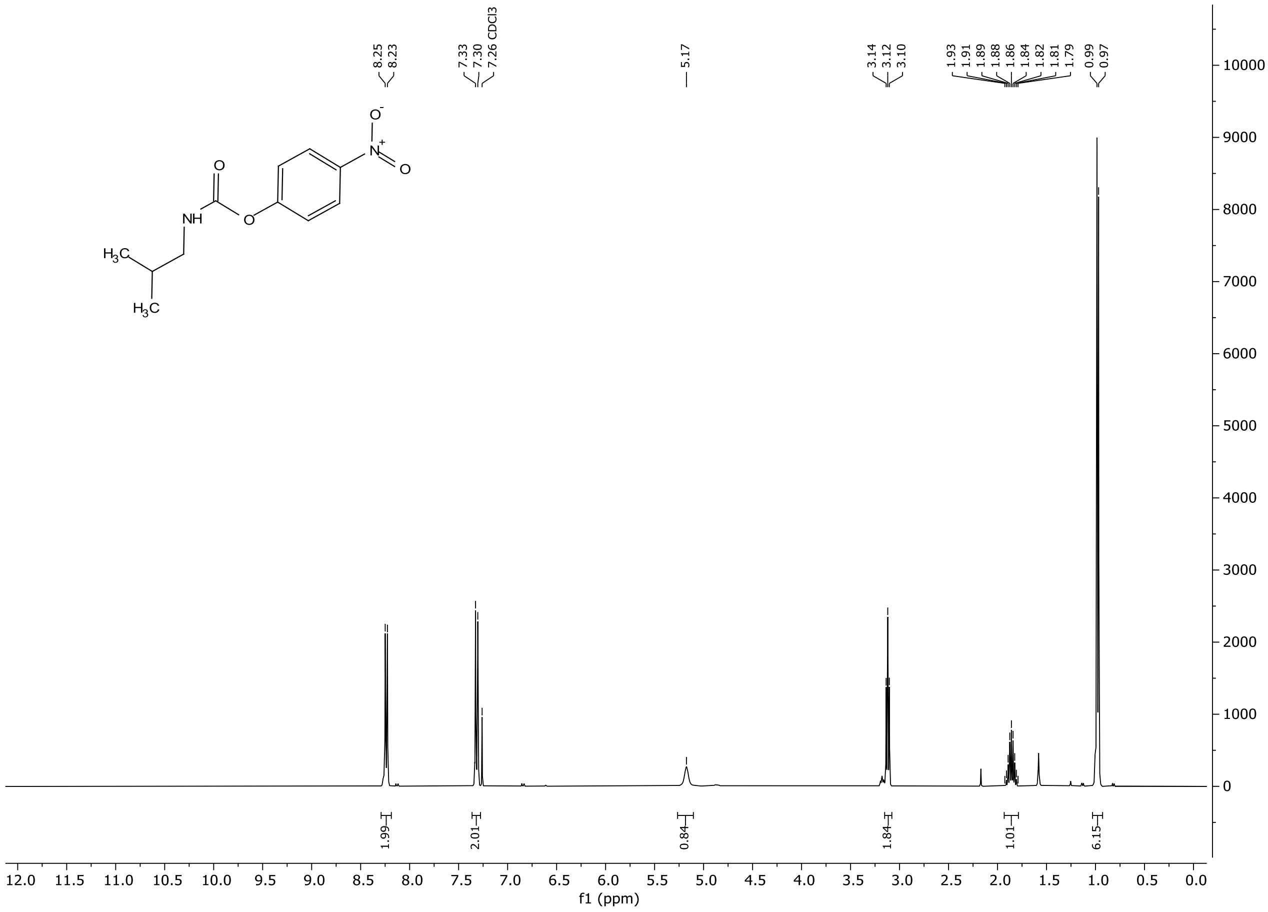

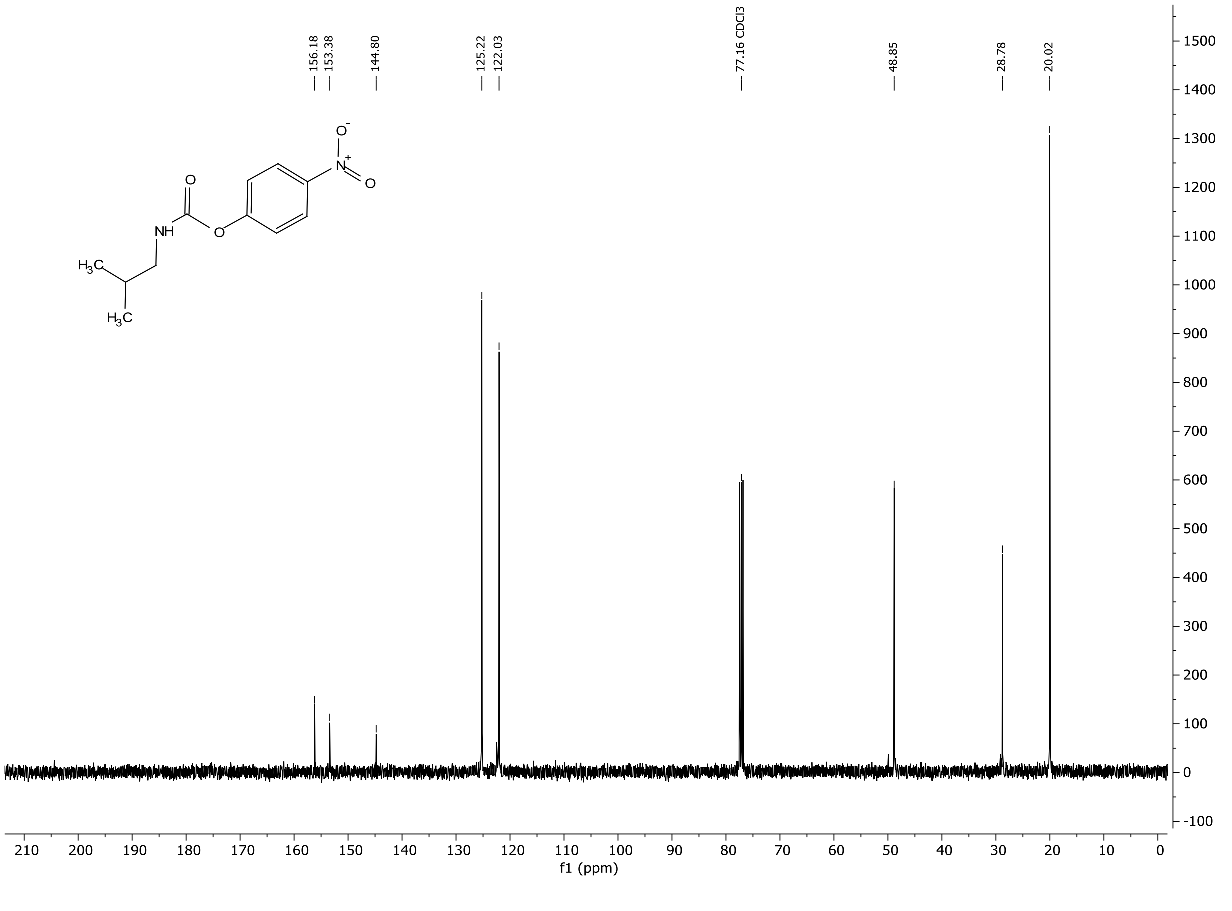

^{1}H and ^{13}C spectra of 4-nitrophenyl (3,3,3-trifluoropropyl)carbamate (3b).

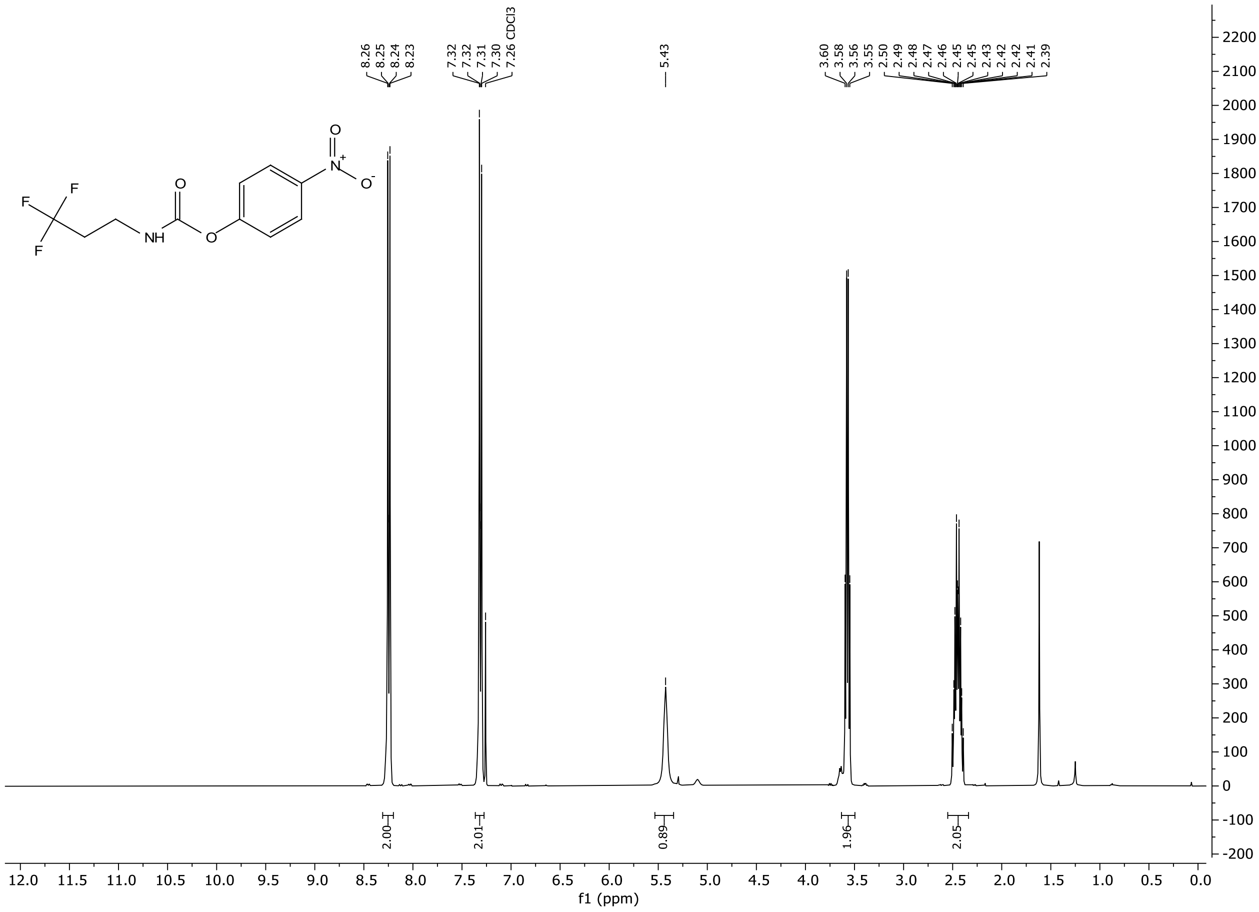

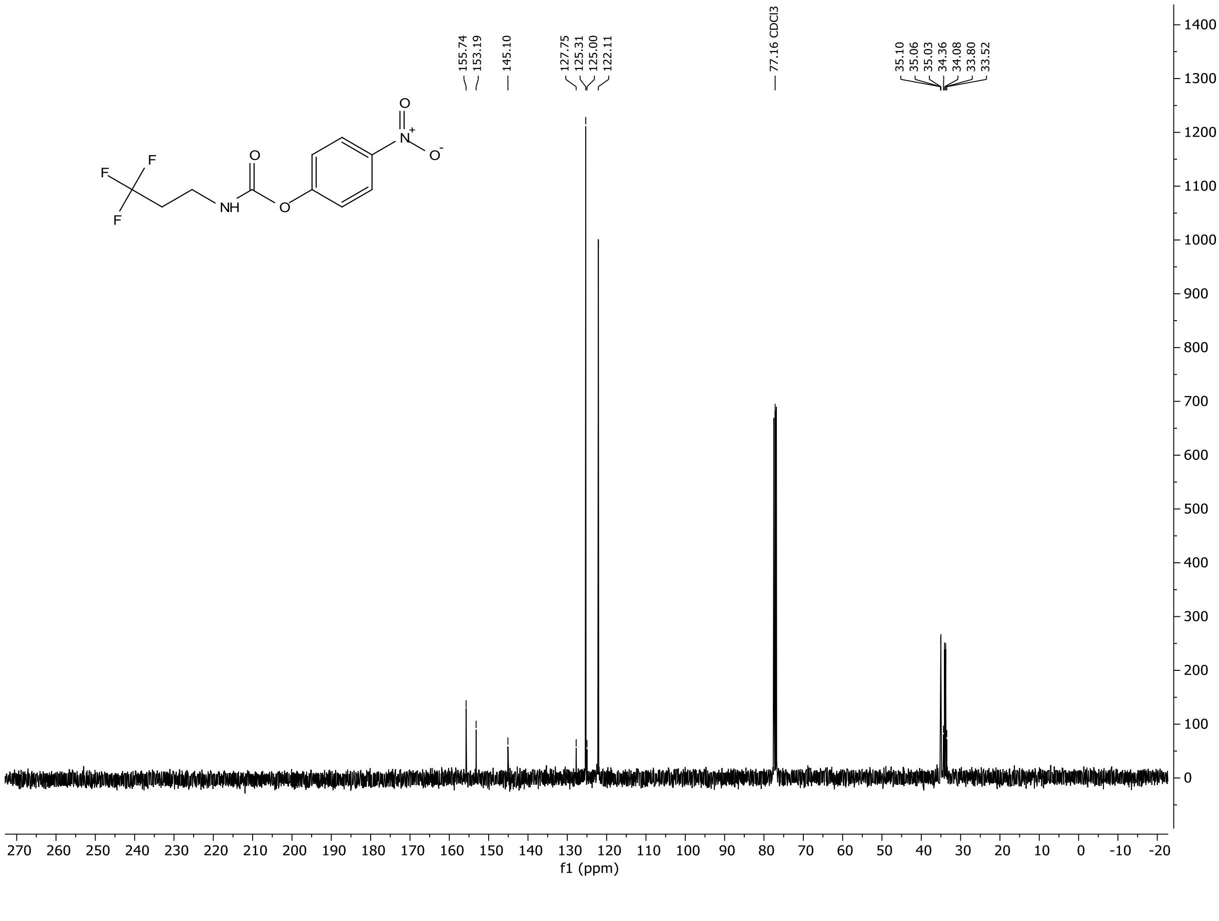

^{1}H and ^{13}C spectra of **4-nitrophenyl (2-fluoroethyl)carbamate (3c).**

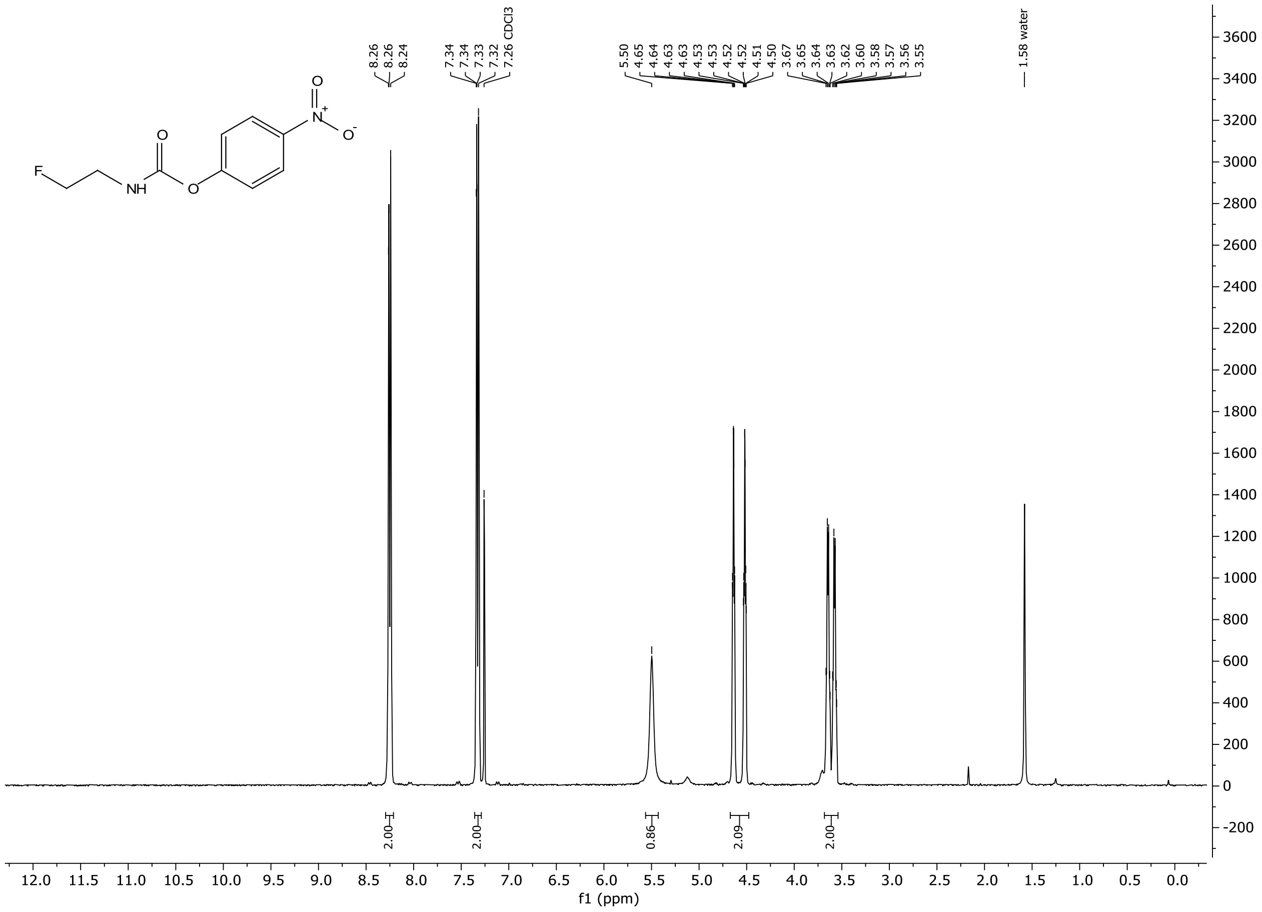

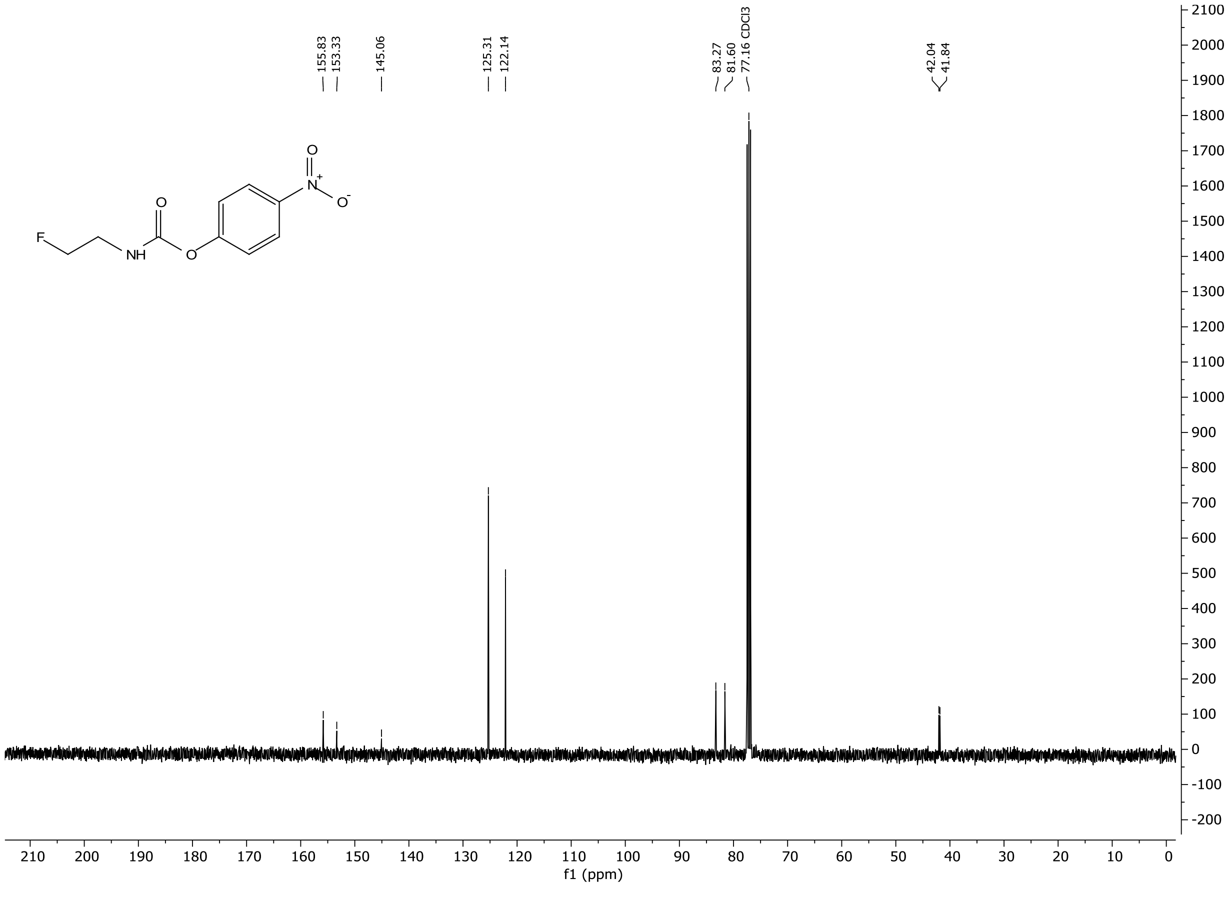

^{1}H and ^{13}C spectra of **4-nitrophenyl (2,2-difluoroethyl)carbamate (3d).**

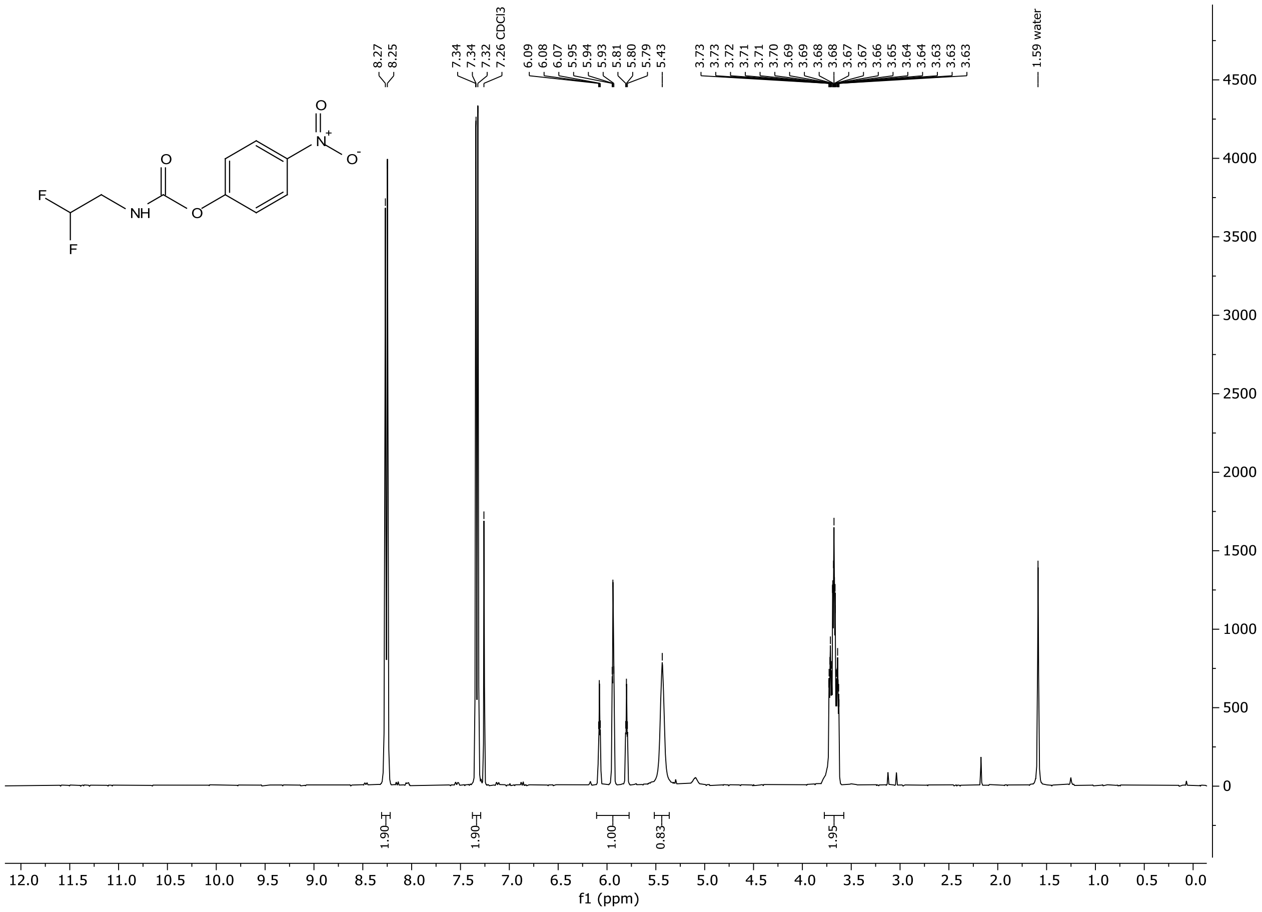

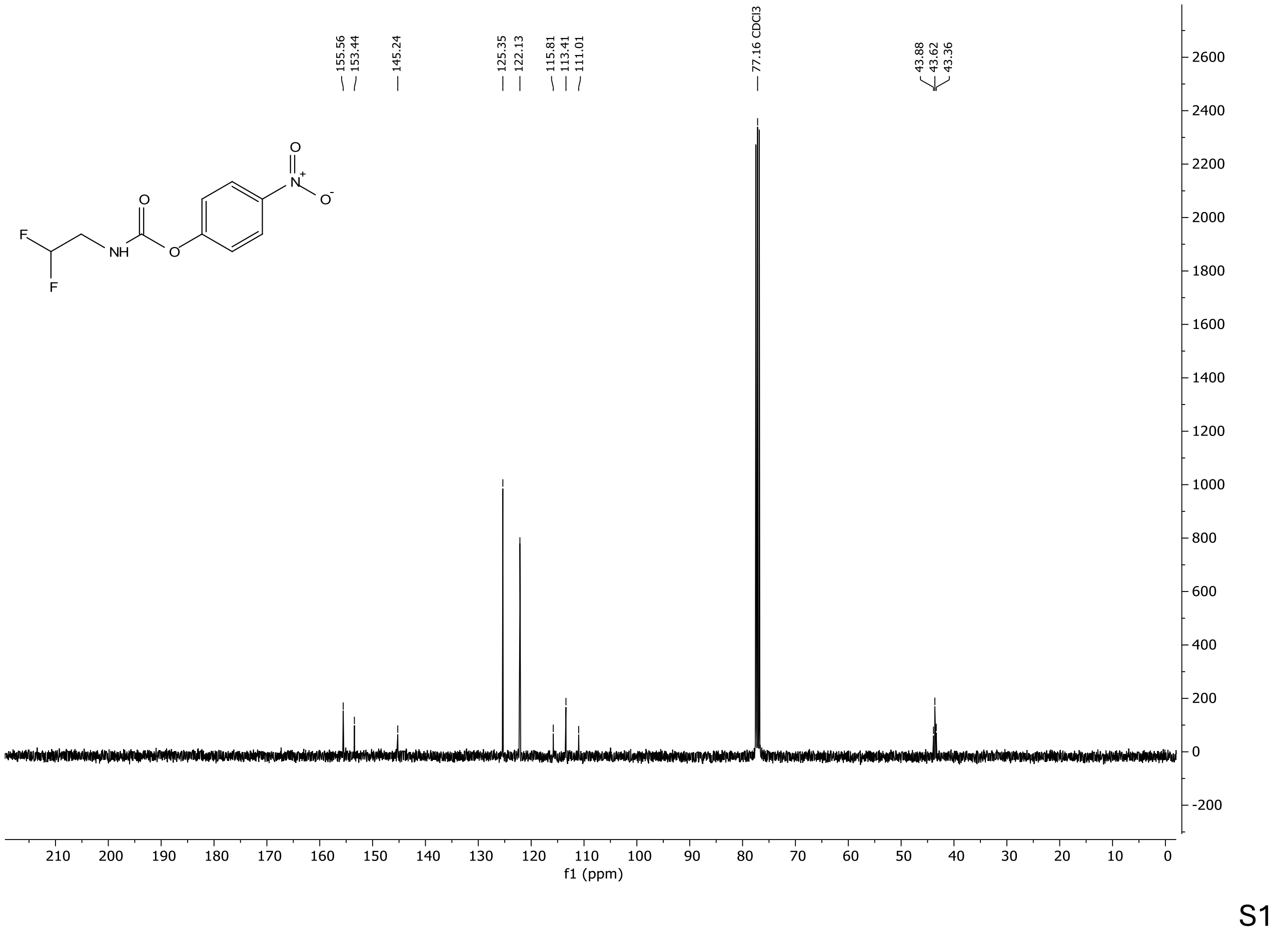

^{1}H and ^{13}C spectra of **4-nitrophenyl (2,2,2-trifluoroethyl)carbamate (3e).**

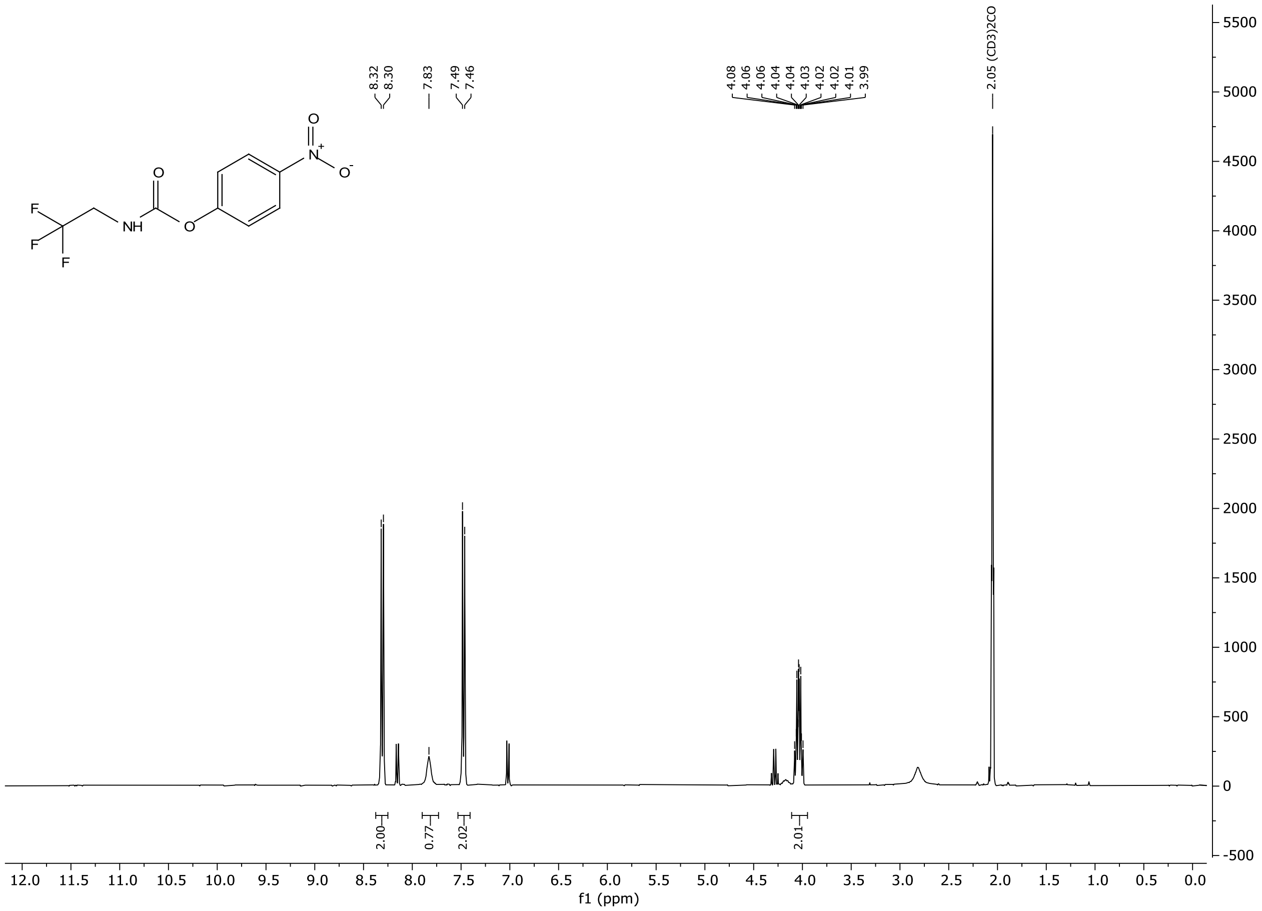

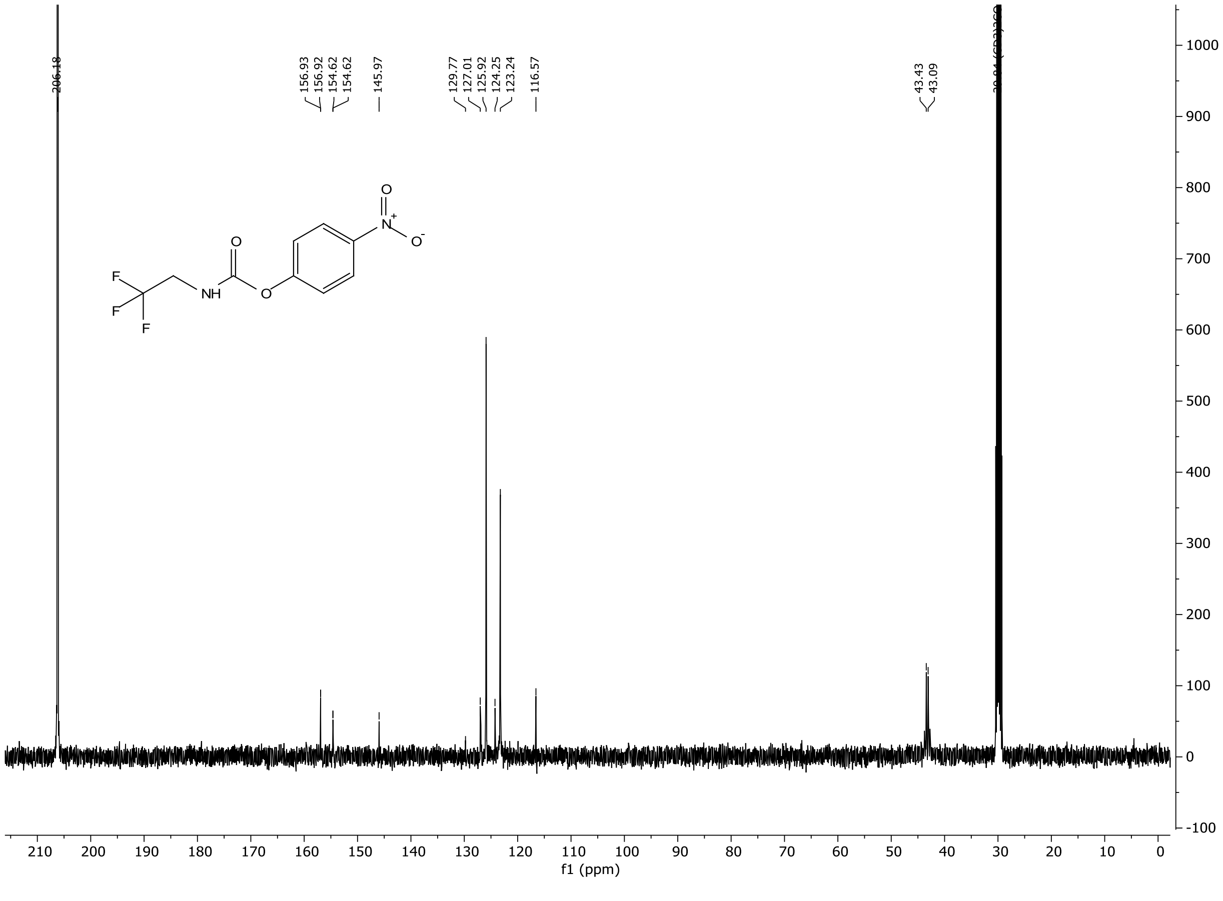

^{1}H, ^{13}C NMR spectra and UPLC chromatogram of **3-(2-(dimethylamino)ethyl)-1H-indol-4-yl isobutylcarbamate hydrochloride (4a).**

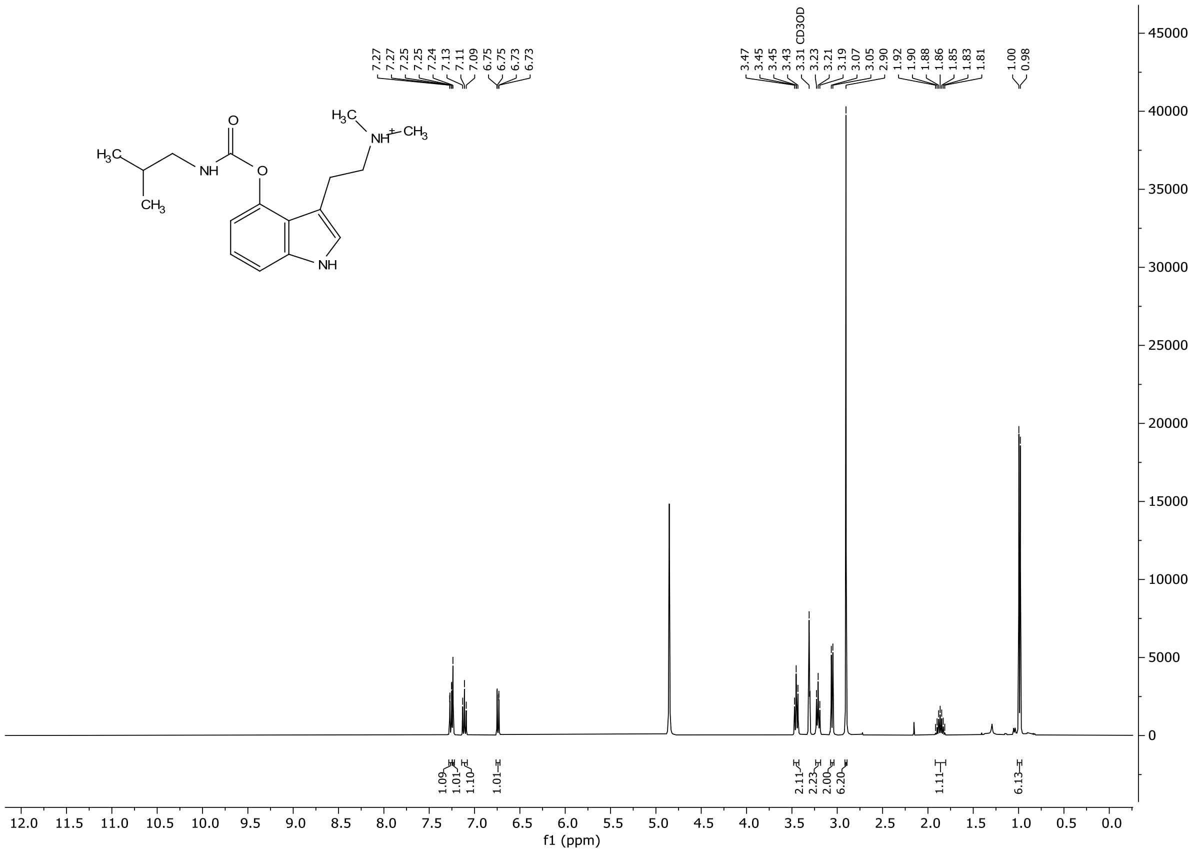

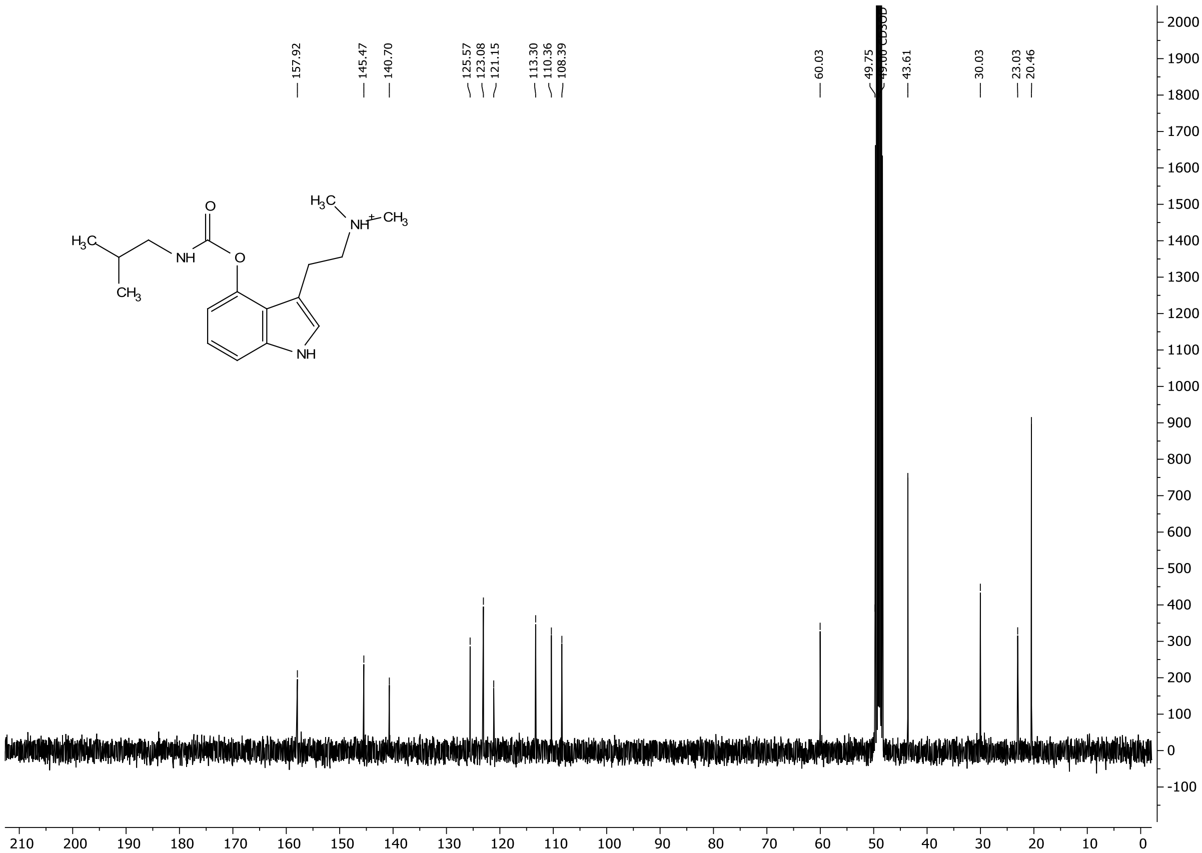

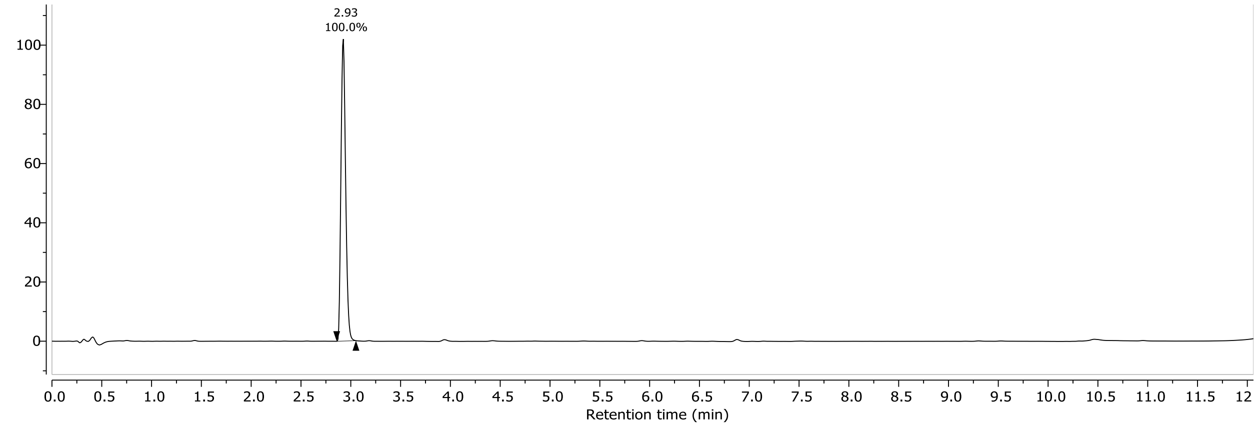

Blank run UPLC

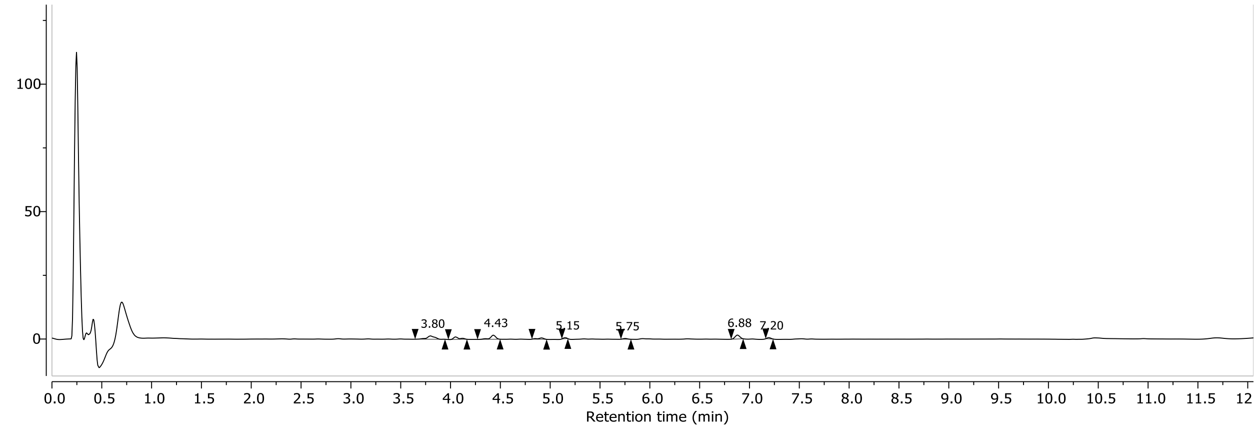

^{1}H, ^{13}C, ^{19}F NMR spectra and UPLC chromatogram of **3-(2-(dimethylamino)ethyl)-1H-indol-4-yl (3,3,3-trifluoropropyl)carbamate (4b).**

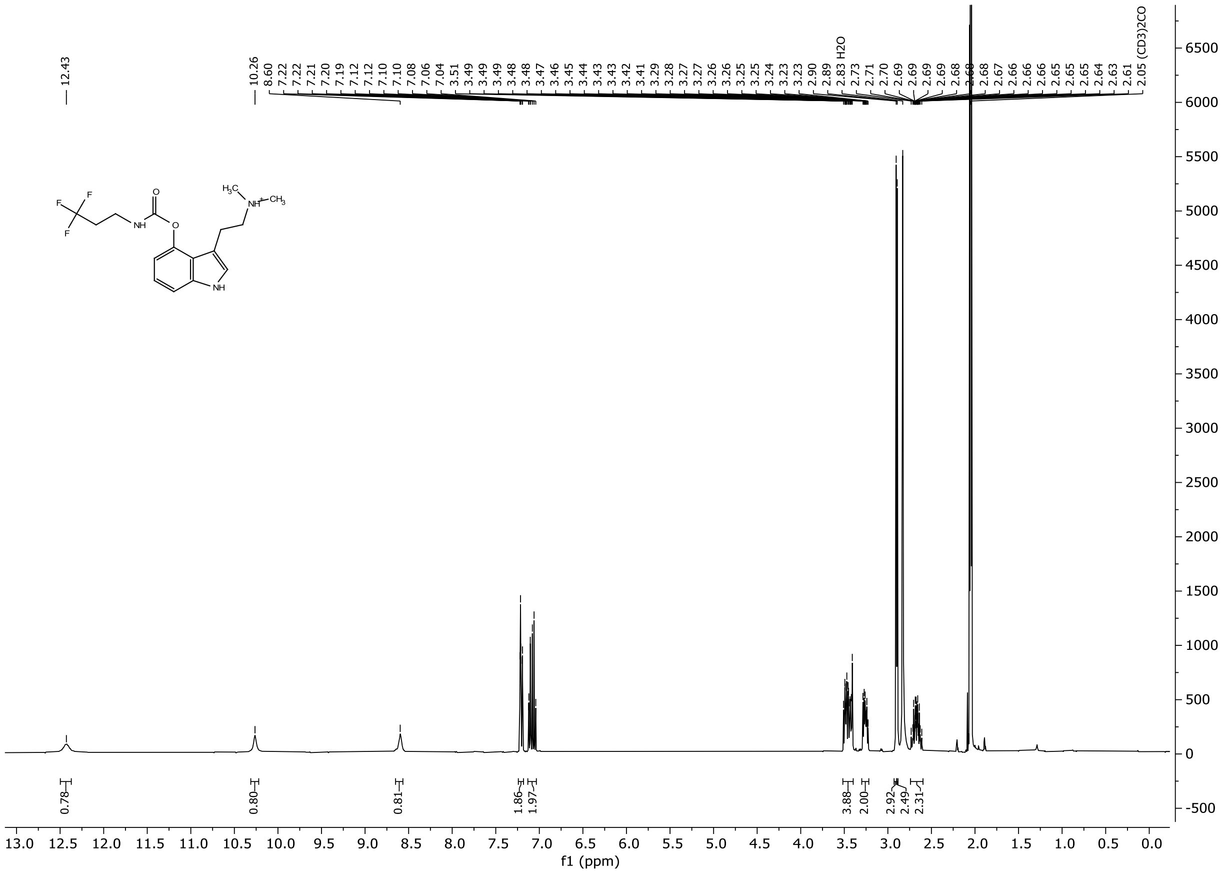

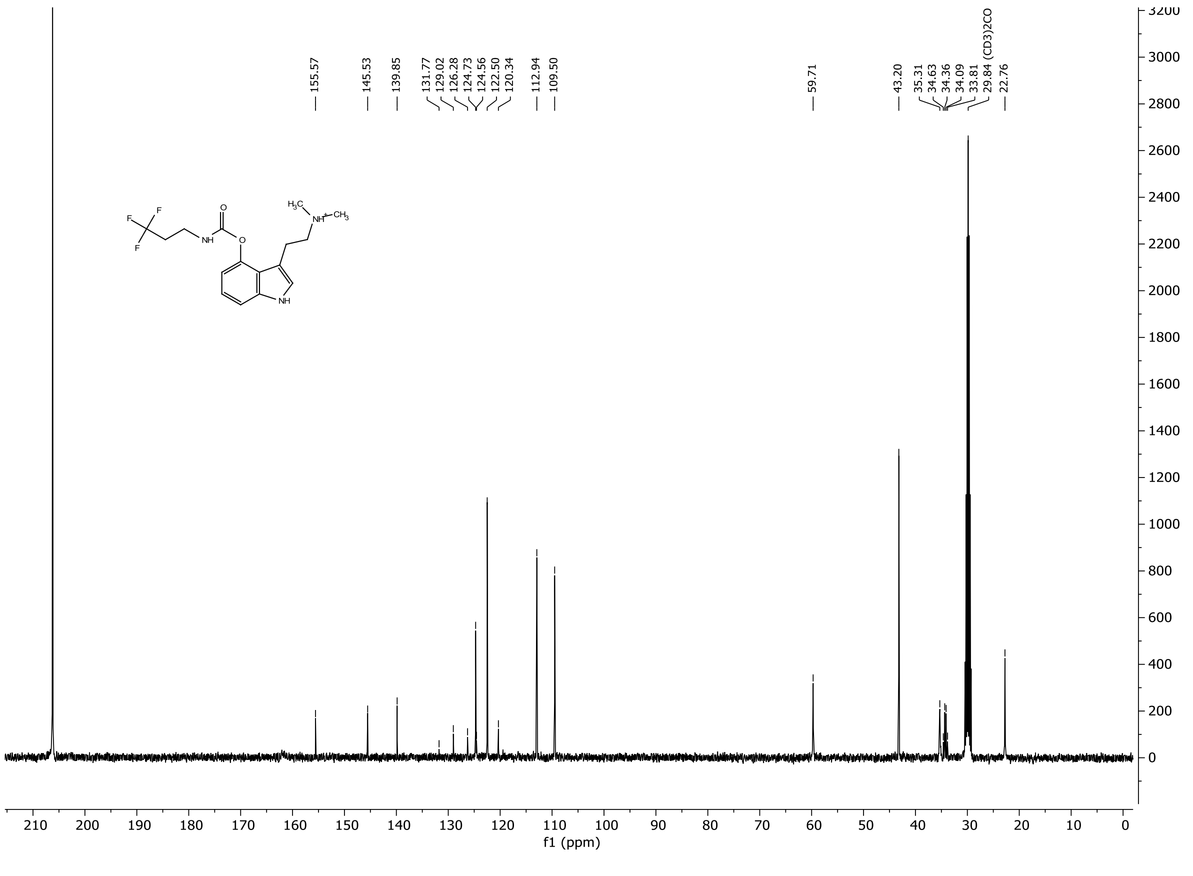

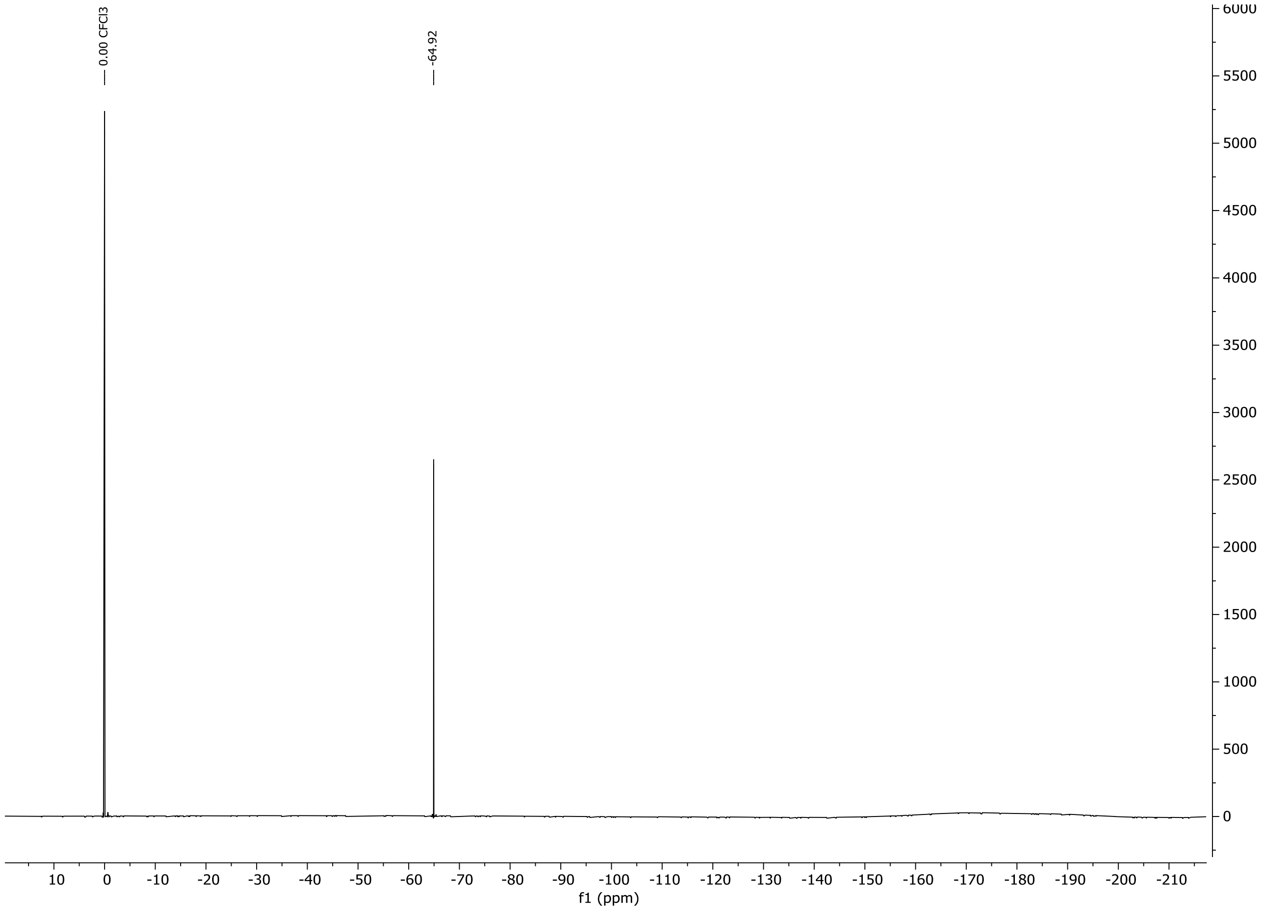

0.00 CFCl3
-64.92
f1 (ppm)

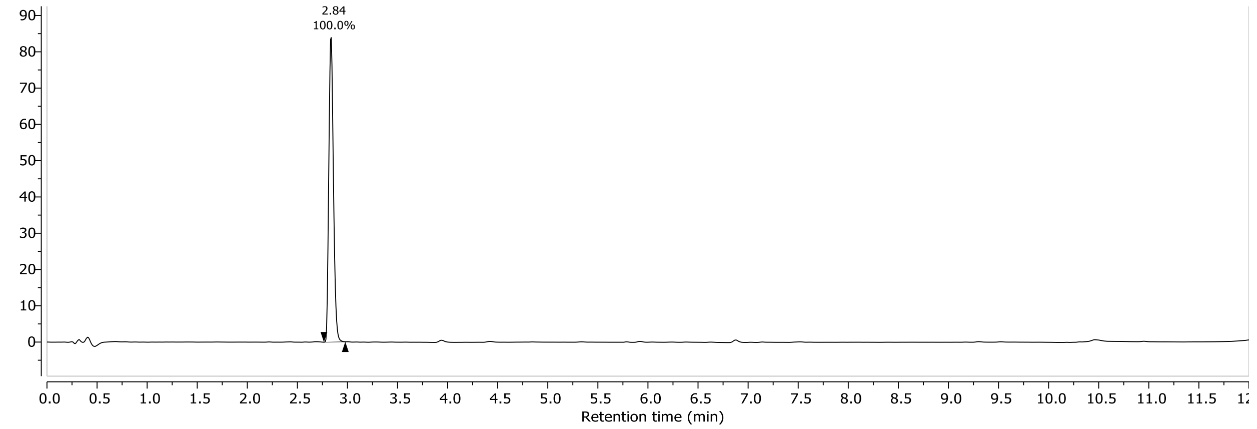

2.84
100.0%
Retention time (min)

^{1}H, ^{13}C, ^{19}F NMR spectra and UPLC chromatogram of **3-(2-(dimethylamino)ethyl)-1H-indol-4-yl (2-fluoroethyl)carbamate hydrochloride (4c).**

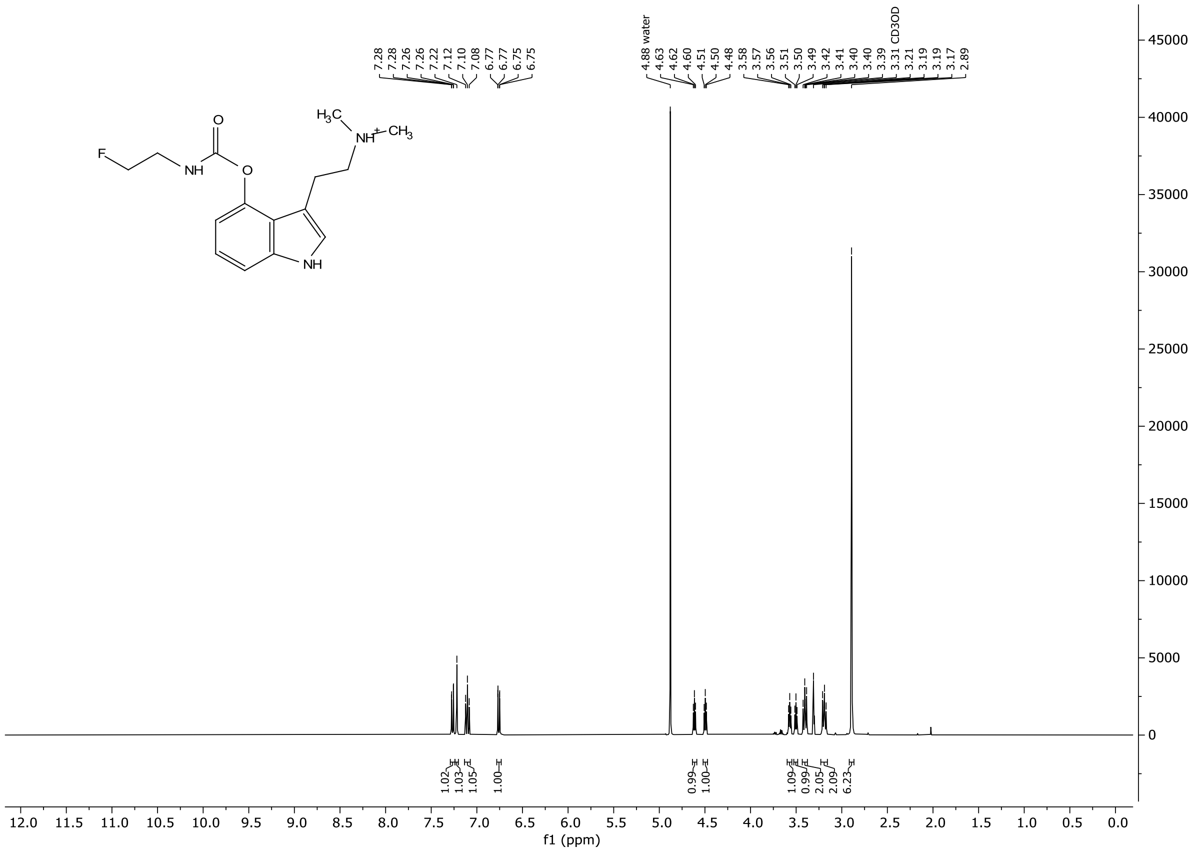

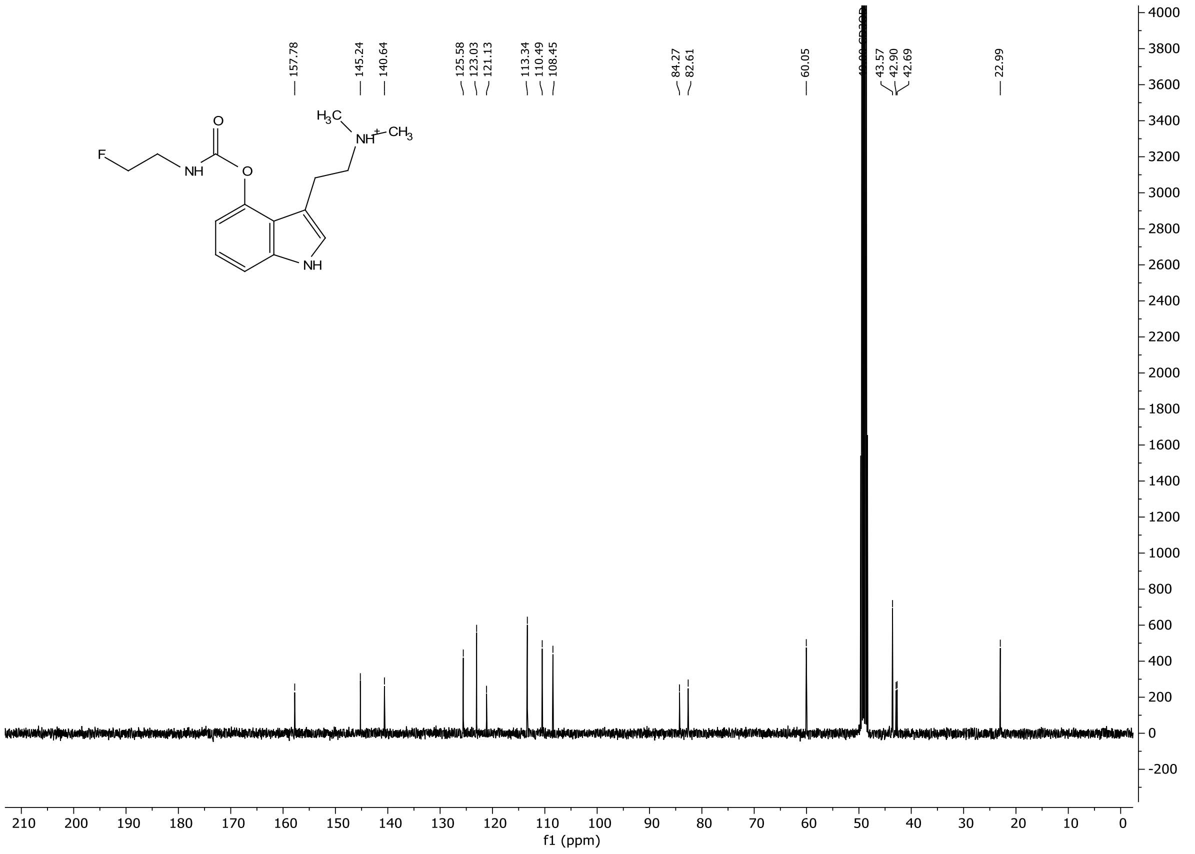

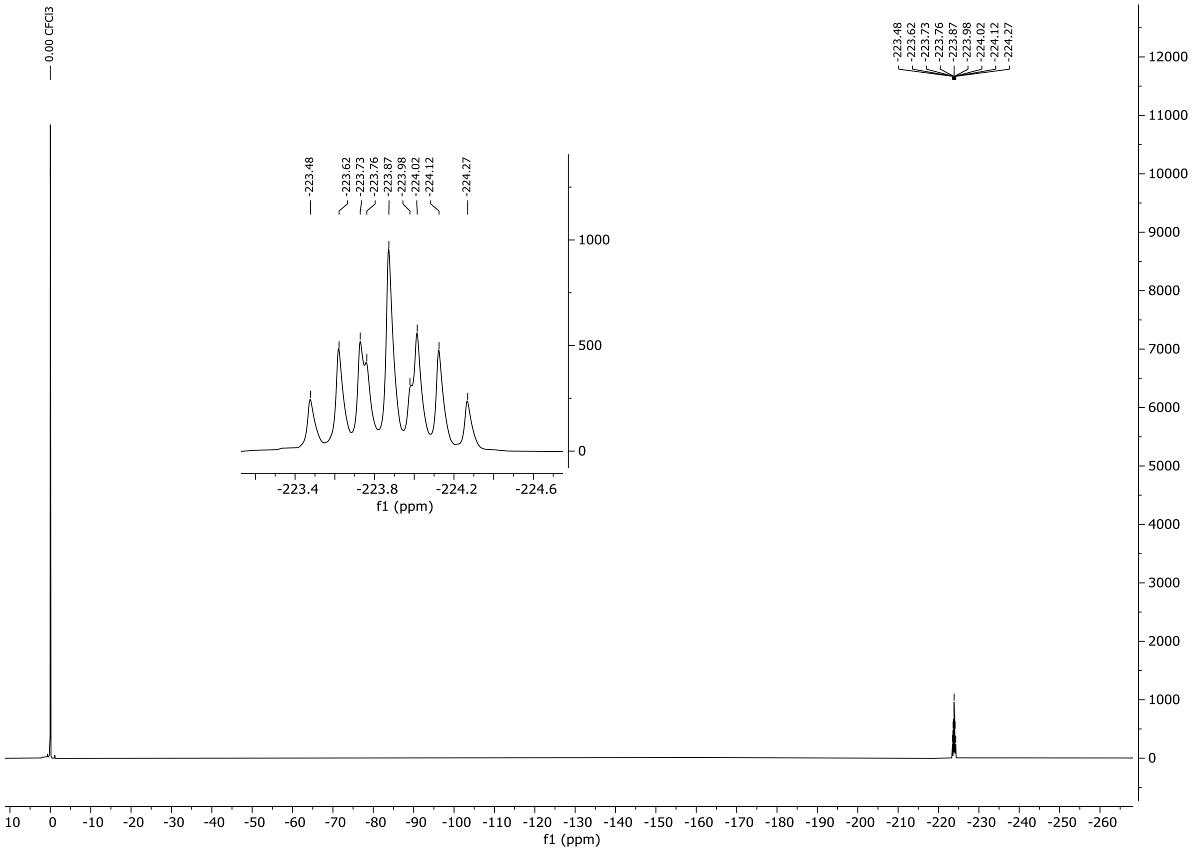
0.00 CFCl3
-223.48
-223.62
-223.73
-223.76
-223.87
-223.98
-224.02
-224.12
-224.27
f1 (ppm)

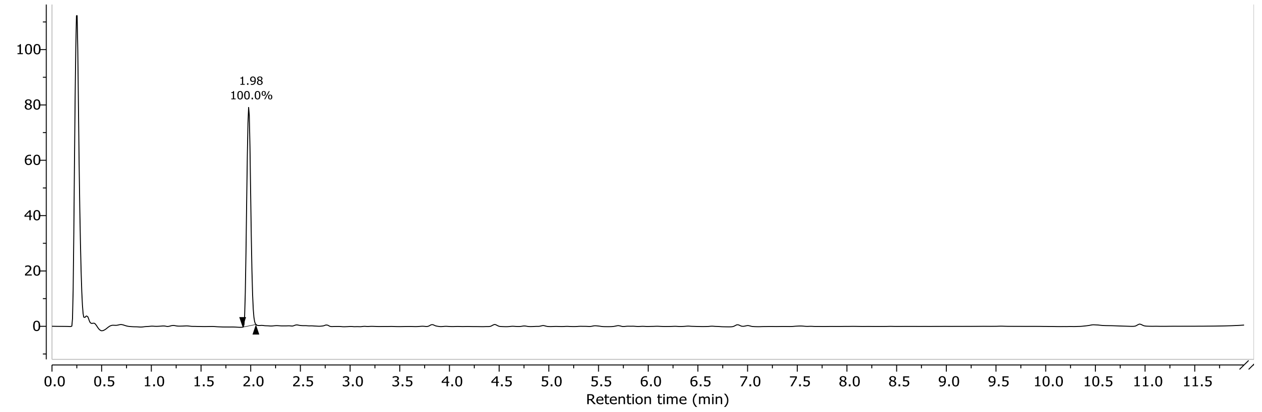
1.98
100.0%
Retention time (min)

^{1}H, ^{13}C, ^{19}F NMR spectra and UPLC chromatogram of **3-(2-(dimethylamino)ethyl)-1H-indol-4-yl (2,2-difluoroethyl)carbamate hydrochloride (4d).**

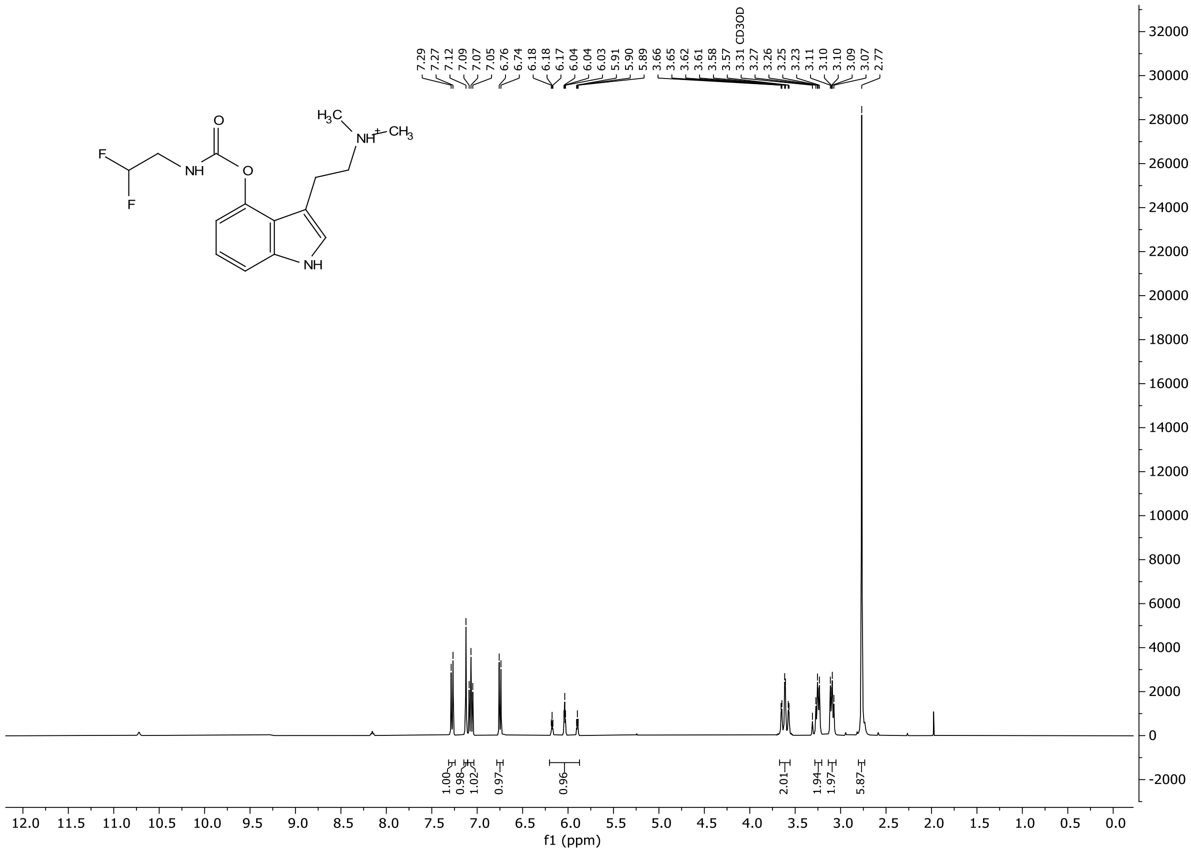

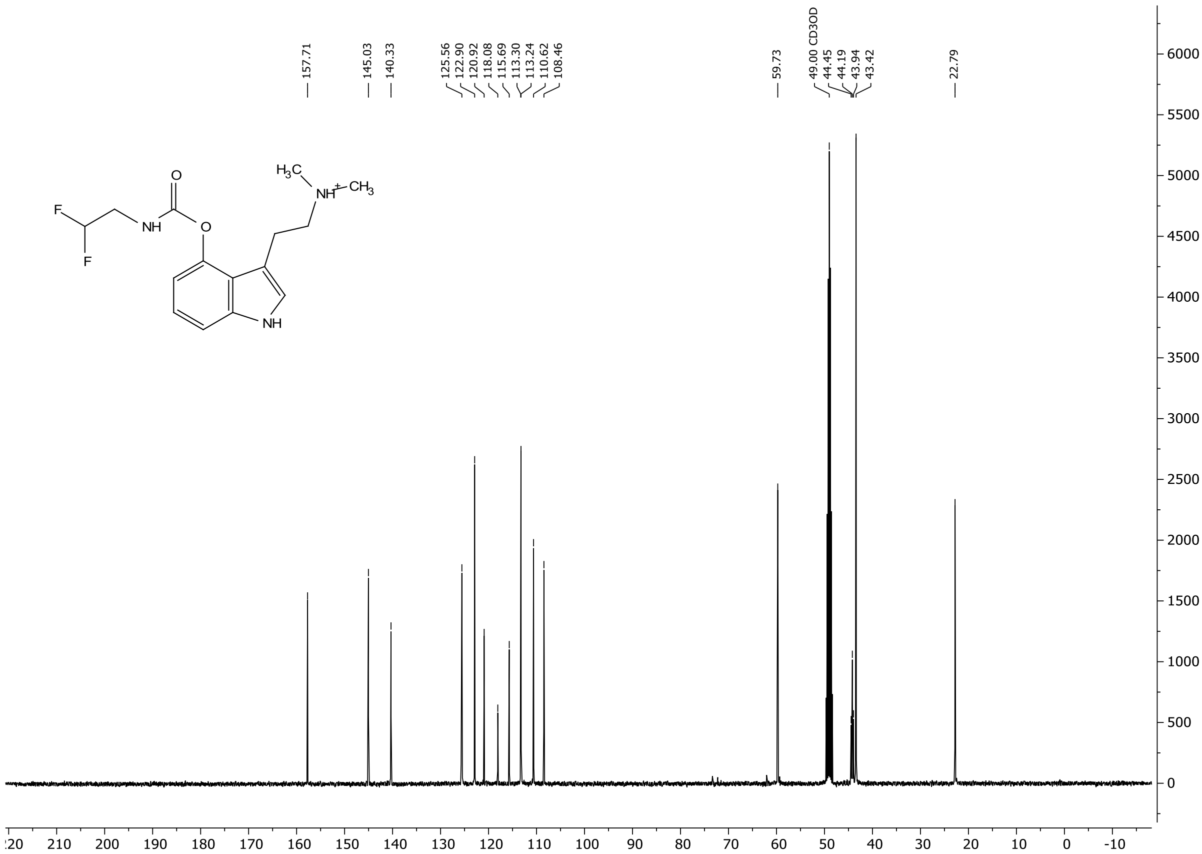

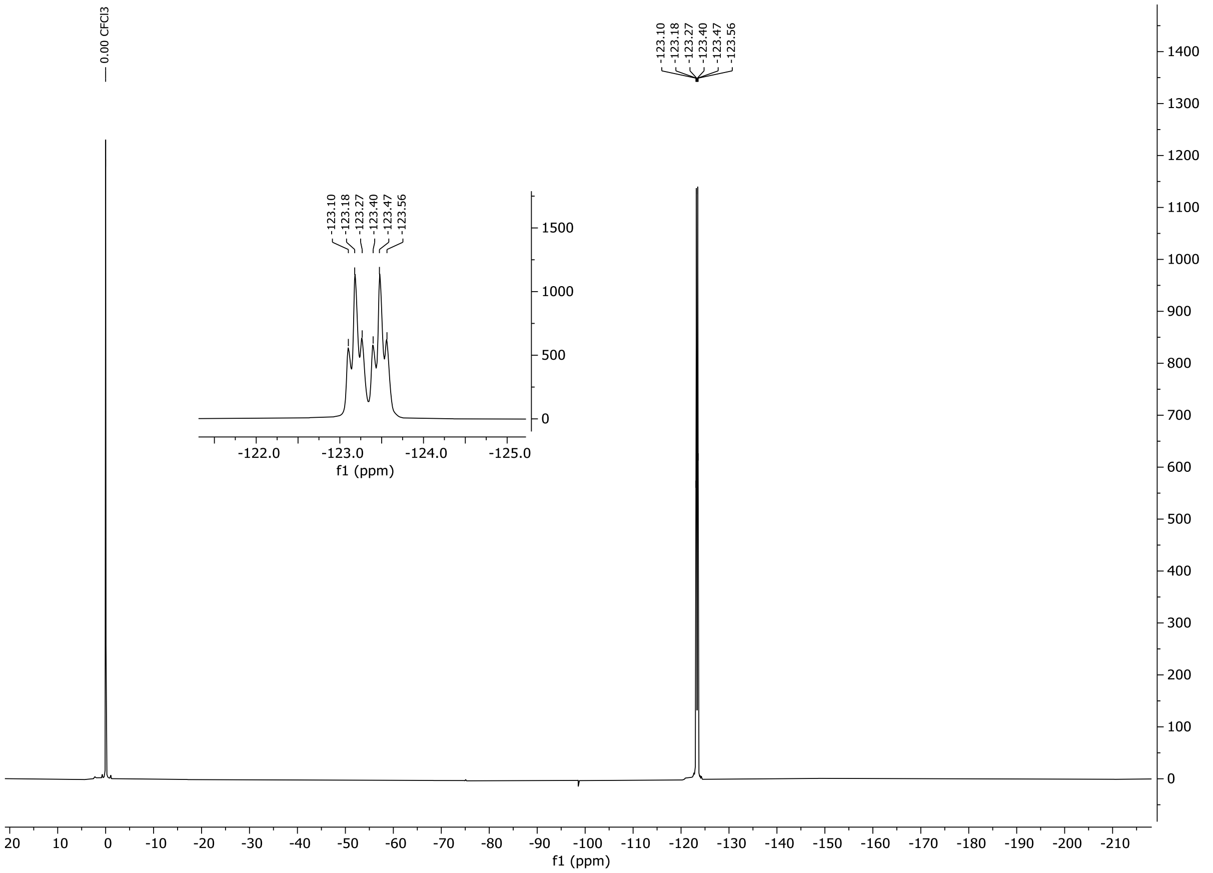

0.00 CFCl3
-123.10
-123.18
-123.27
-123.40
-123.47
-123.56
f1 (ppm)

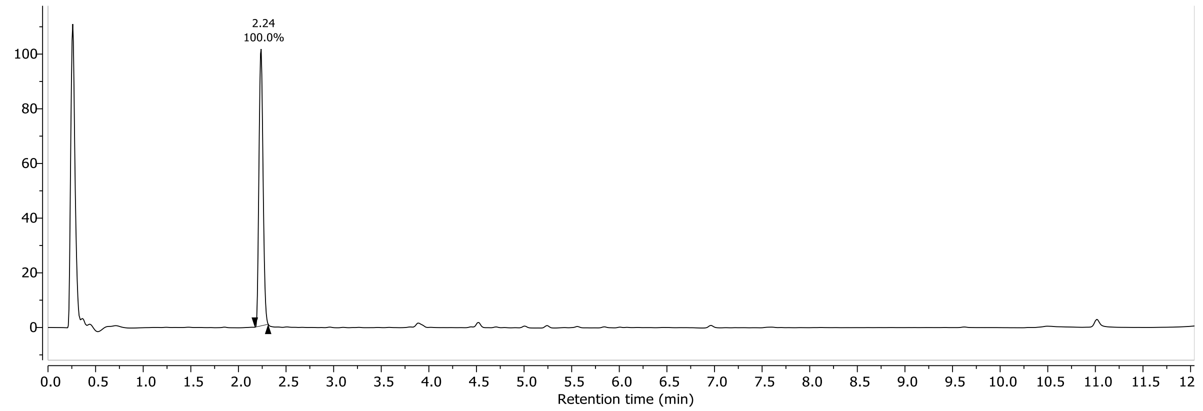

2.24
100.0%
Retention time (min)

^{1}H, ^{13}C, ^{19}F NMR spectra and UPLC chromatogram of **3-(2-(dimethylamino)ethyl)-1H-indol-4-yl (2,2,2-trifluoroethyl)carbamate hydrochloride (4e).**

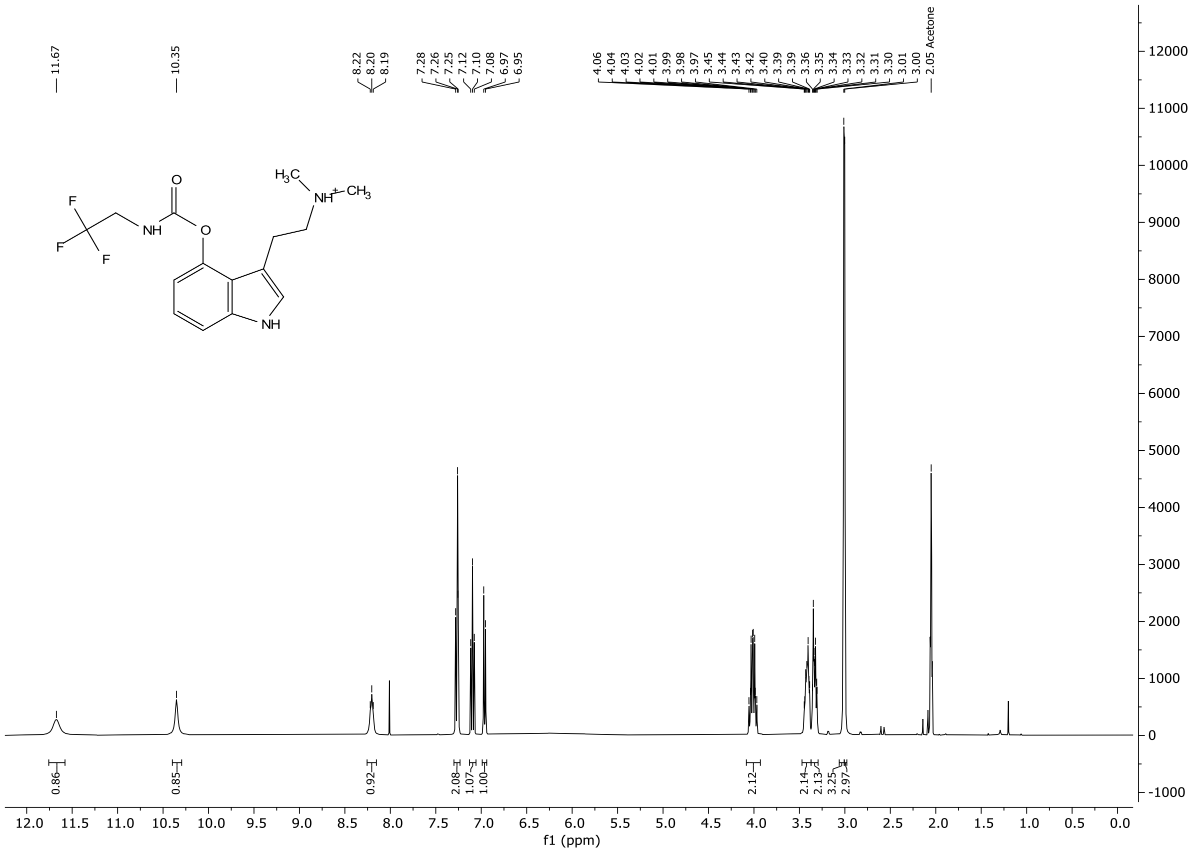

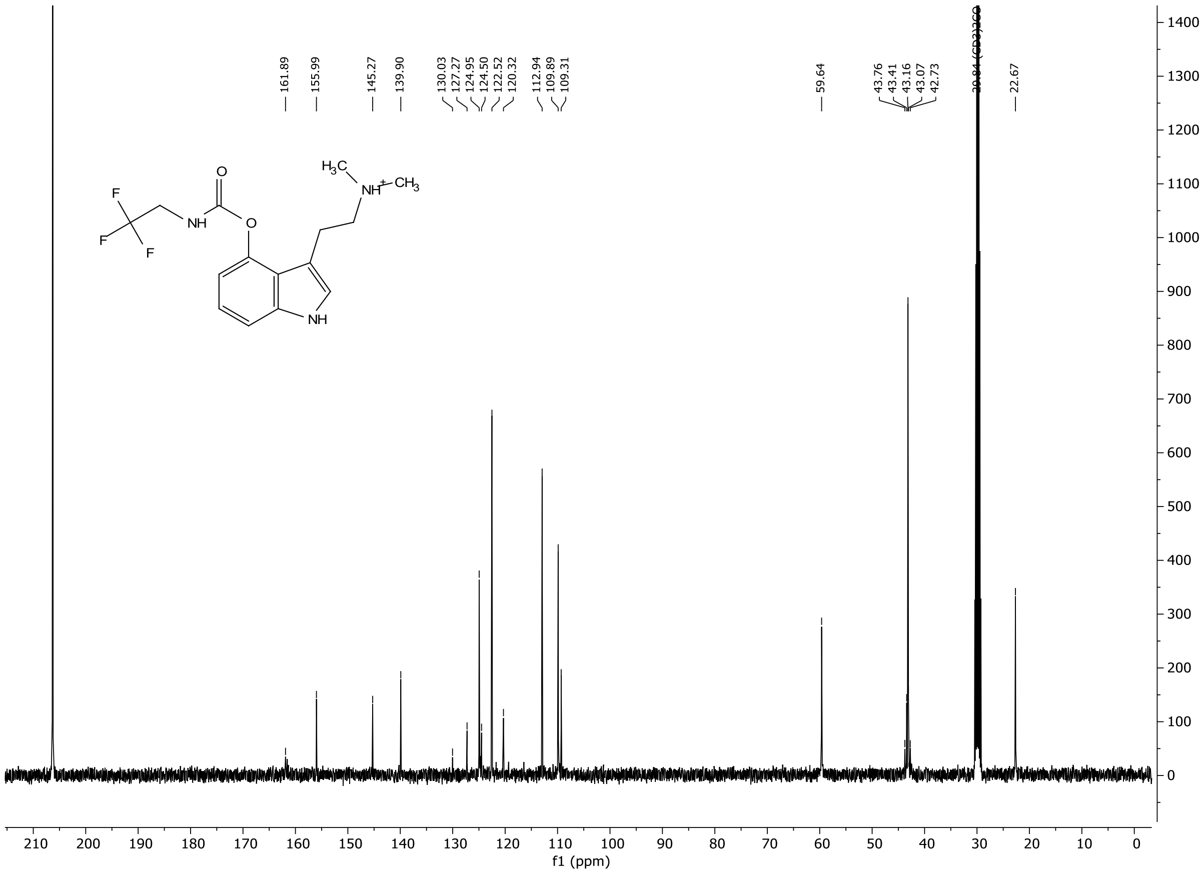

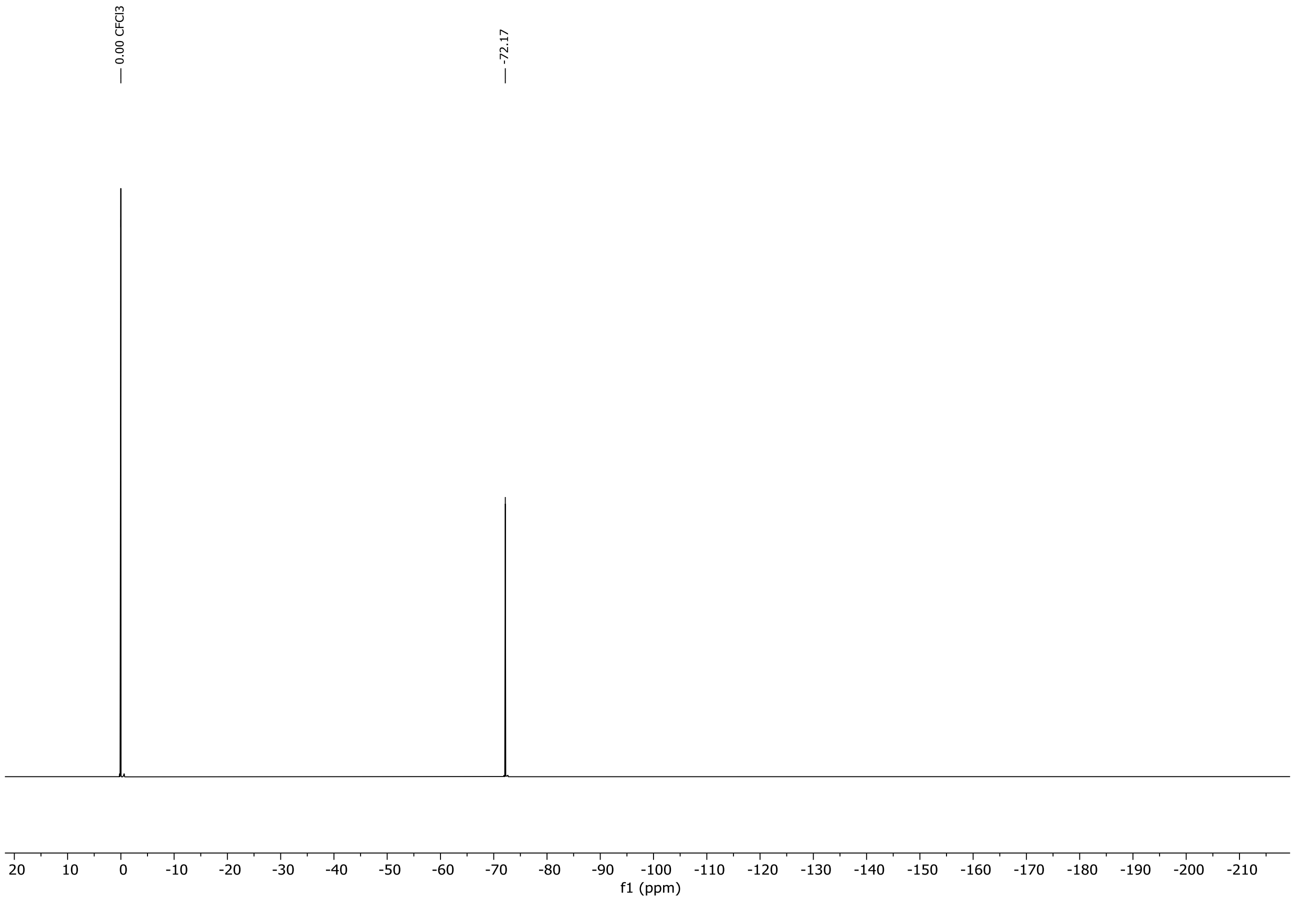
0.00 CFCl3
-72.17
20 10 0 -10 -20 -30 -40 -50 -60 -70 -80 -90 -100 -110 -120 -130 -140 -150 -160 -170 -180 -190 -200 -210
f1 (ppm)

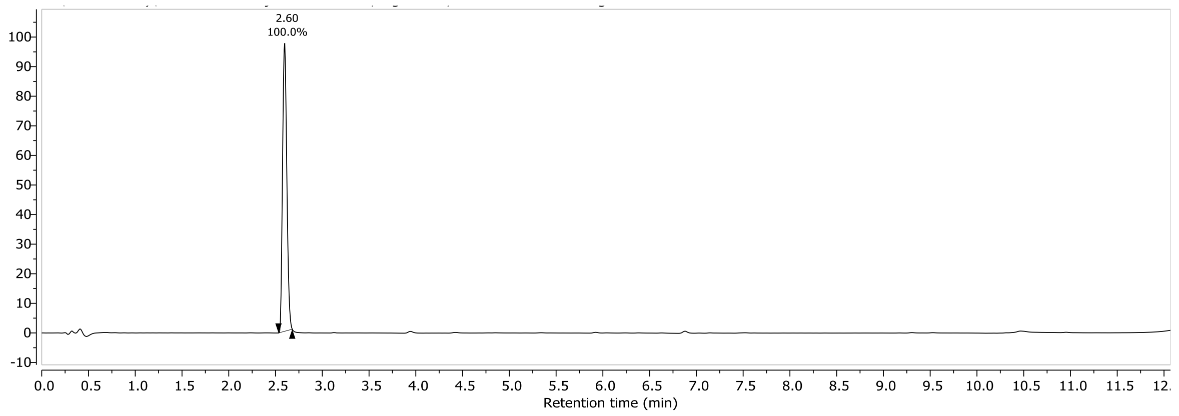
2.60
100.0%
100 90 80 70 60 50 40 30 20 10 0 -10
0.0 0.5 1.0 1.5 2.0 2.5 3.0 3.5 4.0 4.5 5.0 5.5 6.0 6.5 7.0 7.5 8.0 8.5 9.0 9.5 10.0 10.5 11.0 11.5 12.
Retention time (min)

LC-MS/MS analytical method validation

*Materials for method setup and validation of psilocin and compound **4e***

The setup and validation of the analytical method were performed using blank pooled rat/mice plasma and whole homogenate brain samples.

Instrumentation LC-MS/MS

Autosampler	CTC automatic injector (Thermo Fischer Scientific, San Jose, CA, USA)
Gradient	LC-pumps Accela 600 pump (Thermo Fischer Scientific, San Jose, CA, USA)
System controller	Xcalibur 2.1 (Thermo Fischer Scientific, San Jose, CA, USA)
Mass spectrometer	LTQ XL ion trap (Thermo Fischer Scientific, San Jose, CA, USA)
MS-probe	ESI

Analytical parameters

Analytical flow	0.2 mL/min
Analytical column	Hypersil Gold column, 1.9 µm, 50 x 2.1 mm (Thermo Fischer Scientific, San Jose, CA, USA)
Injection volume	5 µL
Time course	14 min
Mobile phase A	0.1% Formic acid in H_2O
Mobile phase B	0.1% Formic acid in MeOH
MS detection	ESI Positive mode

Chemicals

Methanol	LC-MS grade, , Merck Life Science (Germany)
Formic acid (98%)	Merck Life Science (Germany)
Water	Ultrapure water was obtained from a water purification system (PURELAB Classic, ELGA Lab Water, High Wycombe, UK).
Psilocin	Psilocin (CAS No. 520536), Merck Life Science (Germany)
Internal standard	Psilocin-d10 as internal standard (IS) (CAS No. 1435934-64-7), Merck Life Science (Germany)

Table S1. Gradient conditions

Time (min)	% H_2O 0.1% formic acid	% MeOH 0.1% formic acid
0	80	20
9	40	60
10	10	90
11	10	90
12	80	20
14	80	20

Table S2. Mass spectrometry conditions

Compounds	Rt (min)	Precursor ion (m/z)	Product ion (m/z)	Collision energy (volts)
Psilocin	1.0	205.0	160, 58	32
Compound **4e**	3.3	330.1	285, 205, 259	32

Rt = Retention time

Method validation for plasma

Selectivity and sensitivity

The selectivity of the method was tested for each analyte. A representative number of blank plasma samples collected from different animals (n=10) were prepared following the proposed extraction procedure and individually evaluated for interference. The chromatograms of these samples were compared with those obtained from the standard solutions and from the plasma samples spiked with psilocin and compound **4e**, for checking the absence of the potential matrix interference peaks at the retention time of the target analytes. Selectivity was confirmed when the response to interfering substances potentially present in the biological matrix was <20% of the analyte LLOQ and <5% of the IS.

There was no interference effect from the endogenous matrix at the retention time of the two analytes and IS.

Sensitivity was estimated by determining the lower limit of quantification (LLOQ), which is the lowest concentration of the analyte on the calibration curve that could be measured with a precision of 20%, accuracy between 80% and 120% and a signal-to-noise ratio (S/N) of 5.

The LLOQ for psilocin and compound **4e** was 2.5 ng/mL and the %CV values were always <10%.

Accuracy and precision

The accuracy and precision of the method were determined by performing tests on four sets of blank samples fortified with psilocin and compound **4e** at four different concentrations (five replicates each): 2.5 ng/mL

(Lower limit of quantification, LLOQ), 10 ng/mL (Lower quality control of quantification, LQC), 50 ng/mL (Medium quality control concentration, MQC) and 250 ng/mL (High quality control concentration, HQC). Samples were analyzed in the same day for the within-day accuracy and precision evaluation.

The inter-day accuracy and precision were obtained by repeating this experiment consecutively for three different days in the same laboratory, with the same instrument (n=15 replicates for each level of concentration).

Accuracy was calculated by dividing the mean measured value by the fortification level and multiplying by 100 to express the results as a percentage. The acceptance criteria were that each concentration should be within ± 15% for QC samples and within ± 20% for the LLOQ.

Precision was expressed as percentage of the coefficient of variation (%CV), and the acceptance criteria was that each concentration should be less than 15% for QC samples and less 20% for LLOQ.

The data about accuracy and precision are reported in **Table S3**.

Table S3. The intra-day and inter-day accuracy and precision of compounds. Data expressed as mean ± standard deviation. Lower limit of quantification (LLOQ). Low quality control concentration (LQC concentration). Medium quality control concentration (MQC concentration). High quality control concentration (HQC concentration).

Spiked concentration in plasma (ng/mL)		Psilocin			Compound **4e**		
		**Mean measured concentration (ng/mL) ± SD**	**Accuracy (%)**	**CV (%)**	**Mean measured concentration (ng/mL) ± SD**	**Accuracy (%)**	**CV (%)**
Intra-day (n=5)							
LLOQ	2.5	2.43 ± 0.08	97.34	3.47	2.47 ± 0.12	98.64	4.94
LQC	10	10.20 ± 0.74	102.03	7.20	10.28 ± 0.49	102.82	4.78
MQC	50	48.54 ± 3.65	97.07	7.53	48.50 ± 1.41	96.99	2.91
HQC	250	247.33 ± 8.45	98.93	3.42	240.38 ± 10.53	96.15	4.38
Inter-day (n=15)							
LLOQ	2.5	2.50 ± 0.11	99.86	4.51	2.42 ± 0.15	96.62	6.03
LQC	10	9.82 ± 0.62	98.24	6.30	10.34 ± 0.84	103.37	8.15
MQC	50	48.31 ± 2.70	96.63	5.59	48.15 ± 1.94	96.30	4.03
HQC	250	250.58 ± 14.63	100.23	5.84	222.74 ± 17.16	89.10	7.70

Linearity

Method linearity was evaluated by preparing three different matrix matched calibration curves on three different days by spiking the blank biological matrix at seven different concentration levels of psilocin and compound **4e** (2.5, 5, 10, 25, 50, 100, 250 ng/mL) before proceeding with the extraction.

Calibration lines were constructed by plotting the ratio of the analyte area to the IS area or peak area versus the added concentrations for psilocin and compound **4e**, respectively, and carrying out a linear regression analysis. Slope and intercept were determined by the square regression method, and the linearity was considered acceptable when the coefficient of correlation (R^2) was above 0.990. In the **Figure S1 (A, B)** are reported the mean calibration curves (n=3) with the equation, R^2 values and standard deviation. The results show a good linearity as R^2 is >0.999 for both the compounds considered.

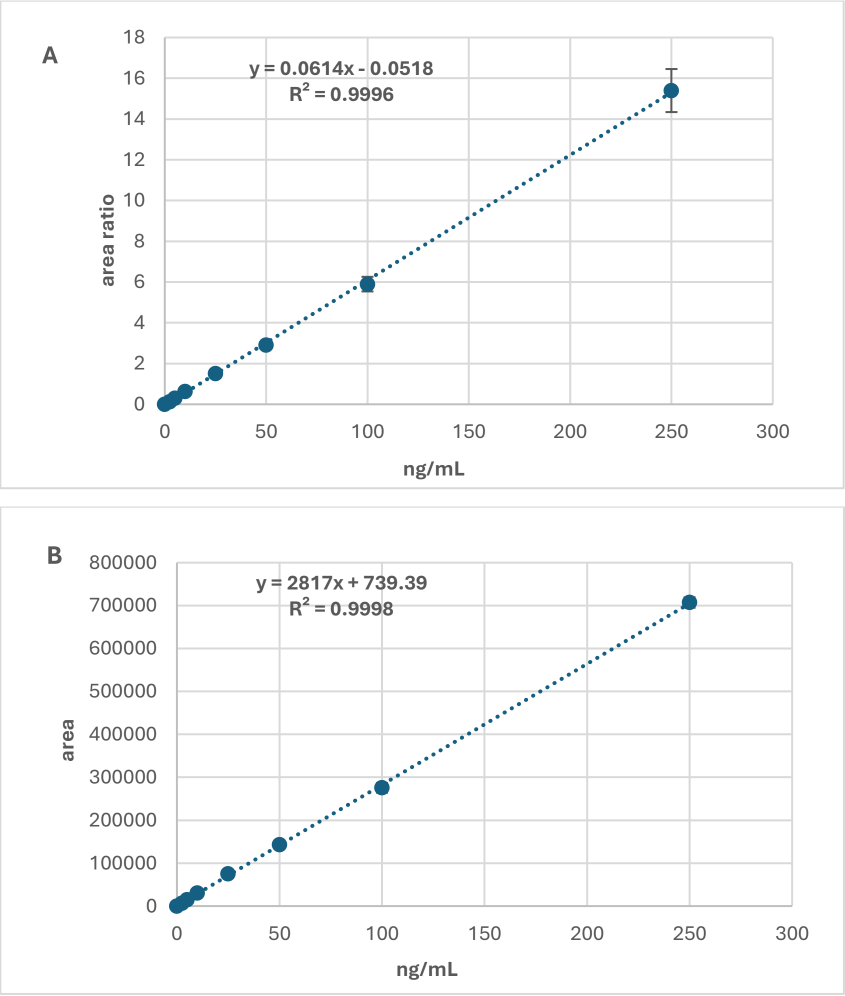

Figure S1. Calibration curves of psilocin (A) and compound **4e** (B)

Recovery and matrix effect

Recovery was determined by comparing the analytical results of the extracted analytes from fortified blank plasma samples (psilocin, compound **4e** and IS were added before the extraction procedure) with un-extracted standards added at the same concentrations in an extracted blank matrix, representing 100%

recovery. To meet acceptance criteria, the recovery should be precise, accurate, and repeatable with %CV less than 15%.

Matrix effects were evaluated by calculating the peak area of the analytes in the presence of matrix (analytes added to blank matrix after extraction), to the peak area in absence of matrix (pure solution of the analyte at the same concentration). The acceptable values should be within 0.8 – 1.2.

Recovery and matrix effect for each analyte were evaluated fortifying each plasma sample at three different QC concentrations (5 replicates for each level): 10 ng/mL (Low quality control concentration, LCQ), 50 ng/mL (Medium quality control concentration, MCQ), 250 ng/mL (High quality control concentration, HCQ).

Three sets of samples were used for determination: one consisting of neat standards (set 1), one prepared in a blank matrix extract and spiked after extraction (set 2) and one spiked before extraction (set 3).

Absolute recovery (REC%) and matrix effect (ME) were calculated using the following formulas:

REC (%) = set 3 area/set 2 area×100

ME = (set 2 area)/(set 1 area)

REC (%): absolute recovery

Set 1 area: area of analytical standard prepared in mobile phase

Set 2 area: area of blank matrix extract spiked with analytes after extraction

Set 3 area: area of blank matrix spiked with analytes before extraction

ME: Matrix effect

Since many samples required dilution before injection, a study on the effect of dilution was also performed. The accuracy has been evaluated after dilution 1:5, 1:10 and 1:20 of the extracted blank plasma samples fortified at three different levels.

Mean extraction recoveries, matrix effect and accuracy after dilution (1:5, 1:10 and 1:20) of the extracted plasma samples are summarized in **Table S4**. All the values respect the acceptance criteria (CV%<15%).

Table S4. Mean extraction recovery, matrix effect, and dilution effect at three levels. Data expressed as mean ± standard deviation (n=5). Low quality control concentration (LQC concentration). Medium quality control concentration (MQC concentration). High quality control concentration (HQC concentration).

Compound	Plasma concentration (ng/mL)		**Recovery**		**Matrix effect**		**Dilution**		
			Recovery %	CV %	Matrix effect	CV %		Accuracy %	CV %
Psilocin	LQC	10	100.07 ± 9.61	9.60	1.16	13.09	1:20	103.88	8.42
	MQC	50	87.18 ± 8.28	9.50	1.15	4.36	1:10	92.49	4.23
	HQC	250	91.66 ± 7.61	8.31	1.08	1.82	1:5	95.54	6.77
Compound 4e	LQC	10	80.19 ± 4.62	5.76	0.95	6.45	1:20	96.31	4.01
	MQC	50	82.92 ± 2.42	2.92	0.93	2.96	1:10	95.80	1.35
	HQC	250	89.51 ± 4.54	5.07	0.91	2.45	1:5	88.58	5.27

Stability

The stability of psilocin and compound 4e was determined after storing the fortified blank plasma samples or extracted samples under different ambient conditions at different time frames.

The stability experiments were performed at two concentration levels (LQC and HQC). Three replicates of each concentration were prepared and injected for each individual stability study.

Short-term stability was evaluated after leaving the fortified samples for 6 h at room temperature (22°C) before being extracted and after leaving the extracted samples inside the autosampler operated at 11°C for 24h and 48h before being analyzed.

Long-term stability test was performed by keeping the spiked samples at −80°C for 1 or 2 months before being extracted. The freeze–thaw stability was determined by analyzing the fortified sample after 3 freeze–thaw cycles in three consecutive days (thawed at 24 h interval).

All the data were compared with freshly prepared QC samples at LQC and HQC levels.

Samples were considered stable if the accuracy of the measured concentration was within 100% ± 15% of the nominal concentration measured at time zero with a CV% of ≤15%.

The results obtained are shown in **Table S5**.

All the values respect the acceptability criteria for all the conditions tested. The CV% for these three compounds are always <15%.

No significant levels of degradation products were detected during the sample preparation process.

Table S5. Stability data of psilocin and compound **4e**.

Storage conditions	Concentration (ng/mL)		Measured concentration (ng/mL, mean ± SD)	Accuracy (%)	CV (%)
**Psilocin**					
Short term stability (6h at room temperature)	LQC	10	8.94 ± 1.16	89.38	12.99
	HQC	250	228.44 ± 10.33	91.38	4.52
Post preparative stability (24h at autosampler)	LQC	10	9.62 ± 0.83	96.22	8.60
	HQC	250	253.93 ± 3.28	101.57	1.29
Post preparative stability (48h at autosampler)	LQC	10	9.58 ± 1.16	95.77	12.08
	HQC	250	248.03 ± 20.10	99.21	8.10
Freeze-thaw stability (3 cycles)	LQC	10	9.41 ± 1.16	94.07	12.34
	HQC	250	256.66 ± 28.55	102.66	11.13
Long term stability (1 month at -80°C)	LQC	10	9.22 ± 0.25	92.22	2.68
	HQC	250	240.19 ± 5.65	96.08	2.35
Long term stability (2 months at -80°C)	LQC	10	9.89 ± 0.16	98.93	1.62
	HQC	250	248.97 ± 1.99	99.59	0.80
**Compound 4e**					
Short term stability (6h at room temperature)	LQC	10	9.86 ± 0.49	98.63	4.96
	HQC	250	245.30 ± 2.91	98.12	1.19
Post preparative stability (24h at autosampler)	LQC	10	9.20 ± 0.53	92.00	5.75
	HQC	250	247.97 ± 2.25	99.19	0.91
Post preparative stability (48h at autosampler)	LQC	10	8.67 ± 0.22	86.74	2.51
	HQC	250	252.14 ± 20.14	100.86	7.99
Freeze-thaw stability (3 cycles)	LQC	10	9.46 ± 0.59	94.57	6.21
	HQC	250	242.19 ± 3.22	96.88	1.33
Long term stability (1 month at -80°C)	LQC	10	9.69 ± 0.48	96.88	4.92
	HQC	250	240.08 ± 21.31	96.03	8.88
Long term stability (2 months at -80°C)	LQC	10	9.99 ± 0.97	99.91	9.76
	HQC	250	251.82 ± 10.06	100.73	4.00

Method validation for brain

To verify the applicability of the method to the brain matrix, the following parameters were evaluated: accuracy, precision, linearity, recovery and matrix effect.

Intra-day accuracy and precision were evaluated by analyzing blank brain samples spiked at four different levels (5, 10, 50 and 250 ng/mL) with three replicates on the same day **(Table S6).**

Table S6. The intra-day accuracy and precision (%CV) of psilocin and compound **4e**. Data expressed as mean ± standard deviation (n=3).

Spiked concentration in brain (ng/mL)	Psilocin			Compound **4e**		
	**Mean measured concentration (ng/mL) ± SD**	**Accuracy (%)**	**CV (%)**	**Mean measured concentration (ng/mL) ± SD**	**Accuracy (%)**	**CV (%)**
Intra-day (n=3)						
5	4.35 ± 0.20	87.07	4.70	4.62 ± 0.01	92.43	0.25
10	9.02 ± 0.19	90.20	2.11	9.80 ± 0.16	97.96	1.68
50	44.23 ± 2.02	88.47	4.57	49.80 ± 0.83	99.59	1.66
250	252.62 ± 4.46	101.05	1.76	256.08 ± 6.82	102.43	2.66

To validate linearity, calibration curves were freshly prepared over three different working days by spiking the blank whole brain homogenate at seven different concentration levels of psilocin and compound **4e** (2.5, 5, 10, 25, 50, 100, 250 ng/mL) before proceeding with the extraction. In the **Figure S2 (A, B)** were reported the mean calibration curves (n=3) with the equation, R^2 values and standard deviation.

The lower limit of quantification was 2.5 ng/mL for both analytes.

The recovery of the psilocin and compound **4e** from brain tissue, was evaluated by analyzing blank brain homogenate samples fortified with standard solution at four different concentrations (5, 10, 50 and 250 ng/mL; n = 4 for each level) before and after extraction procedure.

The matrix effect (ME) was calculated by dividing the peak areas of the analytes spiked in post-extracted blank brain to the peak areas of the analytes in the mobile phase. Four replicates for three different levels (10, 50 and 250 ng/mL) were considered.

Mean extraction recovery for each analyte in brain is summarized in **Table S7**.

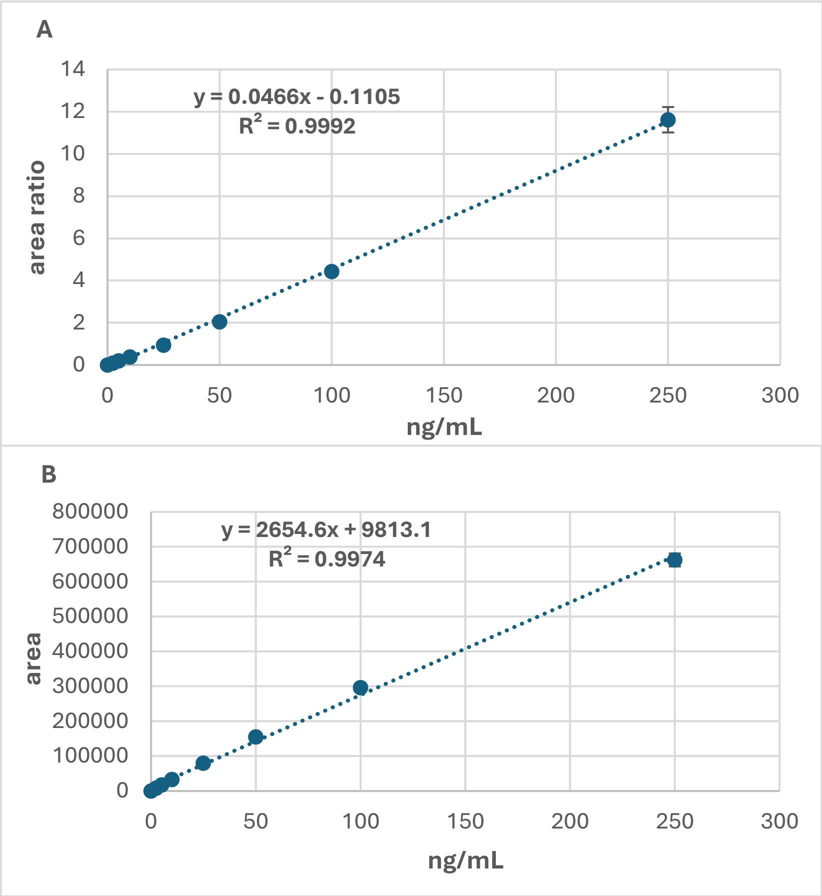

Figure S2. Calibration curves of psilocin (A) and compound **4e**(B)

Table S7. Mean extraction recovery and matrix effect for psilocin and compound **4e** in whole homogenate brain. Data expressed as mean ± standard deviation (n=4).

**Compound**	Brain concentration (ng/mL)	**Recovery**		**Matrix effect**	
		Recovery (%)	CV%	Matrix effect	CV%
Psilocin	5	93.69	6.20	/	/
	10	103.44	8.64	0.91	6.84
	50	95.97	4.29	0.96	11.24
Compound **4e**	5	104.11	0.20	/	/
	10	99.38	1.61	0.89	7.59
	50	98.42	3.55	0.91	7.10

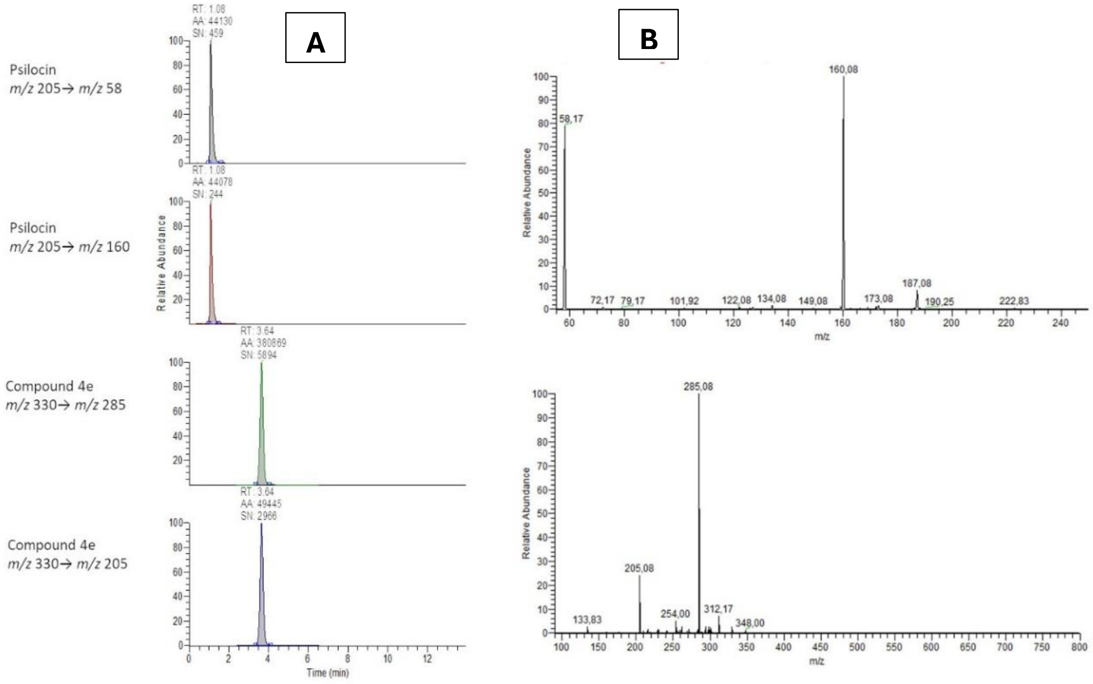

Figure S3. Representative ion chromatograms (A) of standard solution of psilocin and compound **4e** (100 ng/ml in mobile phase) and relative ion spectra $[MH]^+$ (B).

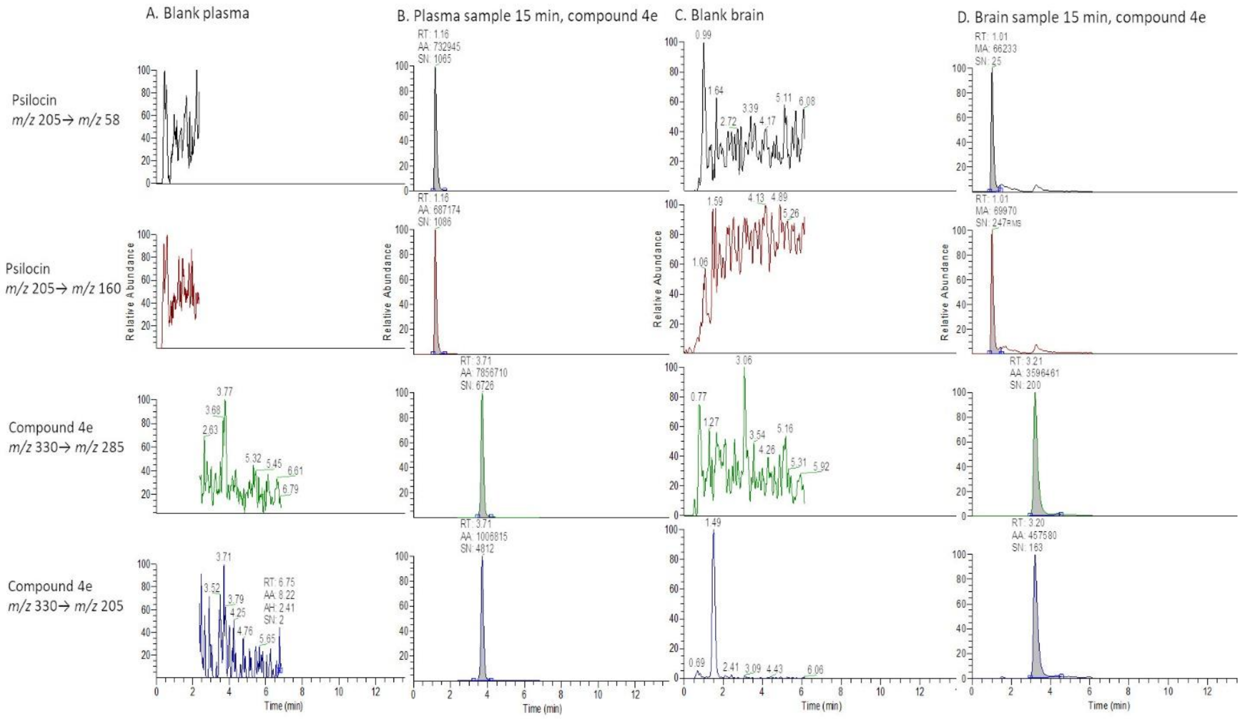

Figure S4. Representative ion chromatograms (obtained using LTQ-XL ion trap) of blank plasma and brain (A, C); and real plasma (B) and brain (D) samples collected 15 min after compound **4e** administration.

The two specific transitions monitored were: *m/z* 205→*m/z* 58 and *m/z* 205→*m/z* 160 for psilocin and *m/z* 330→*m/z* 285 and *m/z* 330→*m/z* 205 for compound **4e**.

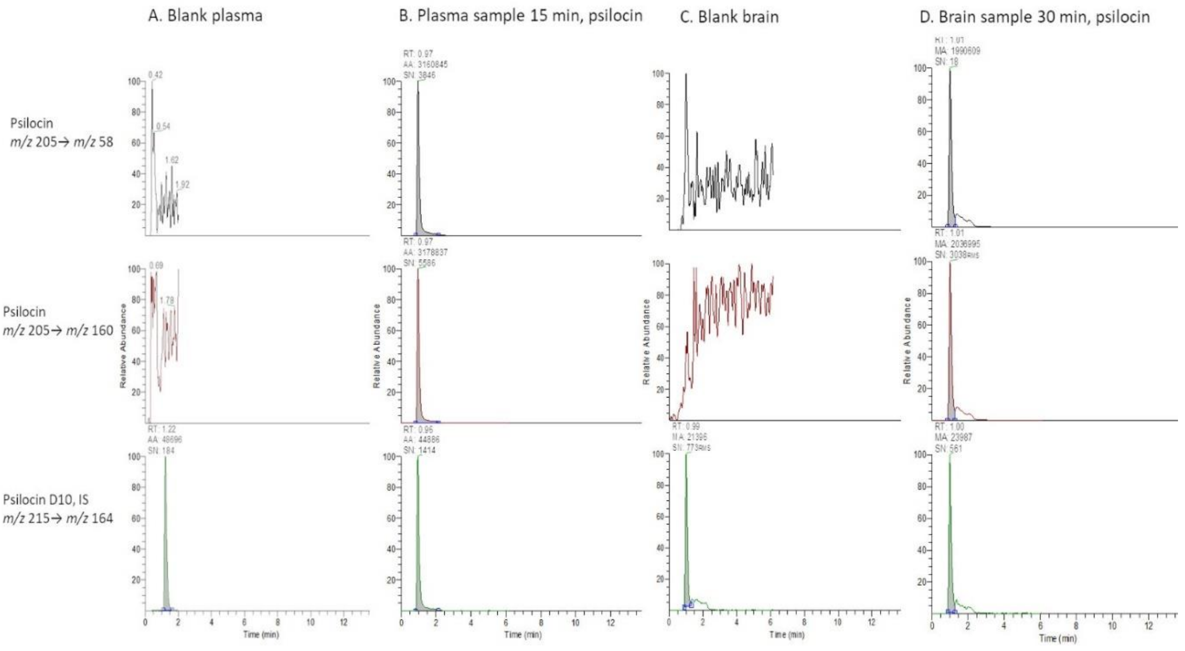

Figure S5. Representative ion chromatograms (obtained using LTQ-XL ion trap) of blank plasma and brain (A, C); and real plasma (B) and brain (D) samples collected 15 and 30 min after psilocin administration, respectively. The two specific transitions monitored were *m/z* 205→*m/z* 58 and *m/z* 205→*m/z* 160 for psilocin and *m/z* 215→*m/z* 164 for internal standard D10.

Experimental data of metabolic stability of PSY and compounds 4a-e in HLMs and S9

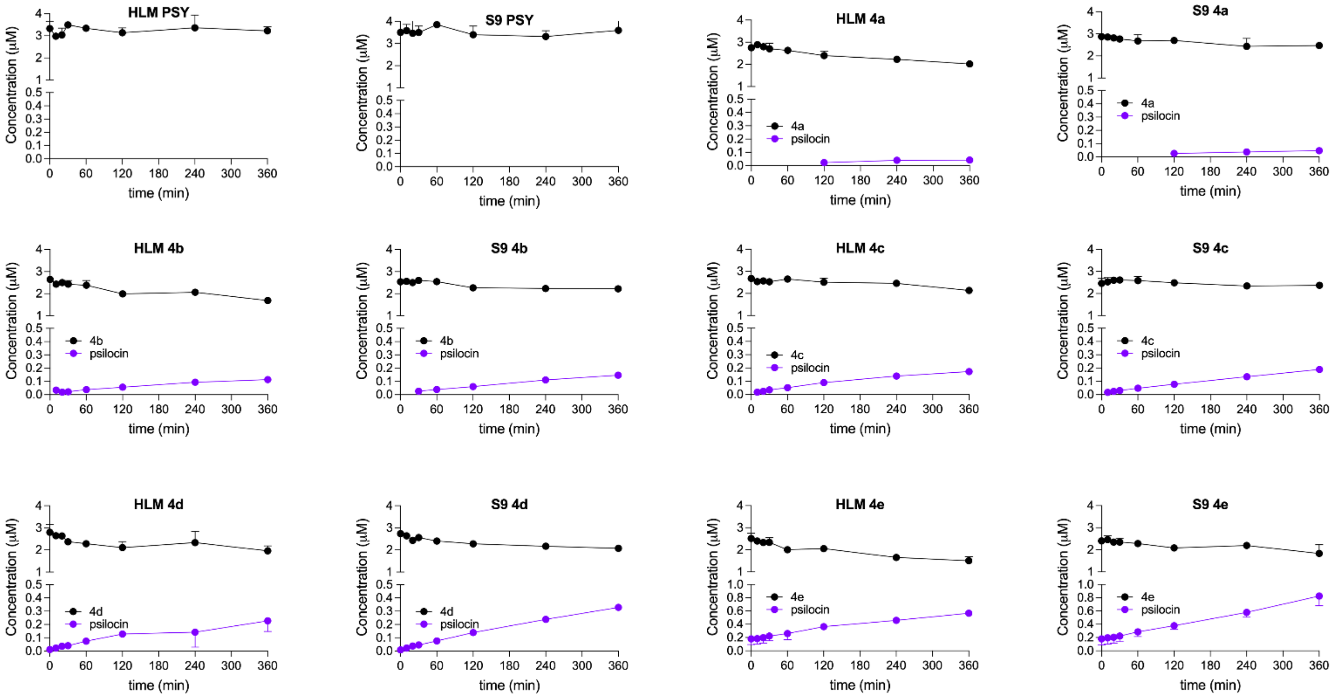

Figure S6. Metabolic stability of **PSY**, **4a**, **4b**, **4c**, **4d**, and **4e** expressed as parent compound and **PSI** concentration in the incubation media at time 0, 10, 20, 30, 60, 120, 240 and 360 minutes.

Experimental data of chemical and plasma stability of PSY and compounds 4a-e and determination of hydrolysis rate constants from experimental data

Interpolation of experimental data with the equation for pseudo-first order reactions: $[C] = [C]_0 * e^{-kt}$ (where: $[C]$: concentration of the compound; $[C]_0$: concentration of the compound at the initial time t_0; t: time), using Origin 8.0 software and non-linear curve fitting. Experimental data are expressed as percentage of the total amount of species recovered at each time point.

Figure S7. Interpolation of data from hydrolysis in HCl, pH 1, 37°C: (A) **PSY**; (B) **4a**; (C) **4b**; (D) **4c**; (E) **4d**; (F) **4e**.

A) PSY

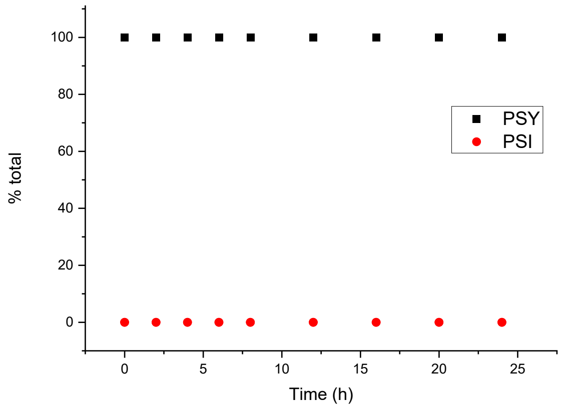

B) 4a

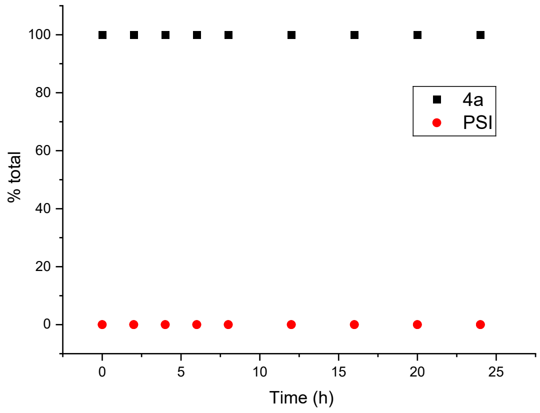

C) 4b

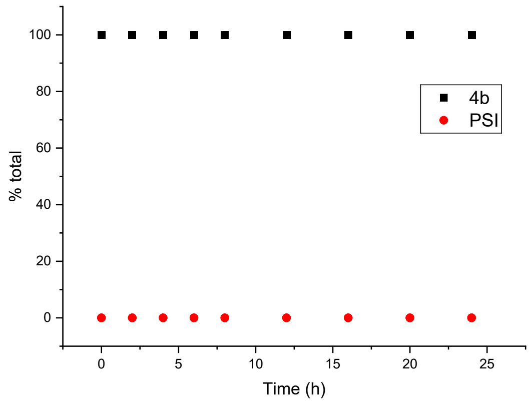

100
80
60
40
20
0
% total
0
5
10
15
20
25
Time (h)
4b
PSI

D) 4c

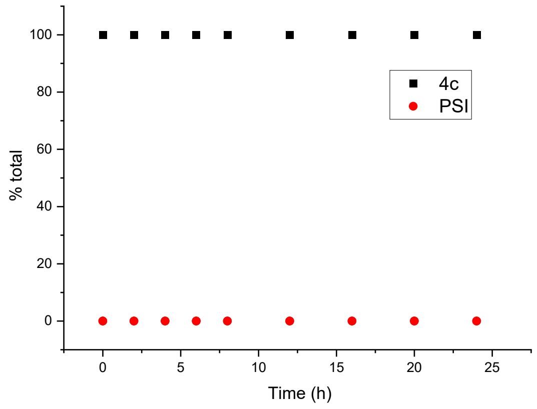

100
80
60
40
20
0
% total
0
5
10
15
20
25
Time (h)
4c
PSI

E) 4d

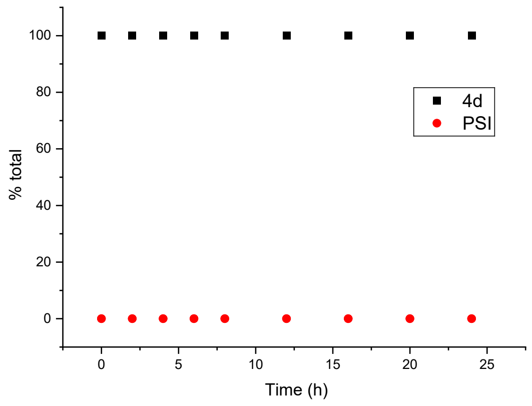

100
80
60
40
20
0
% total
0
5
10
15
20
25
Time (h)
4d
PSI

F) 4e

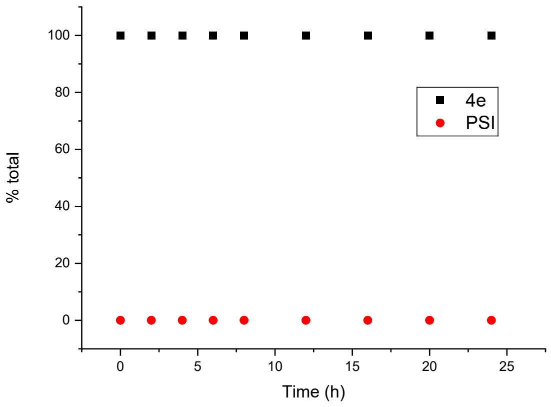

Figure S8. Interpolation of data from hydrolysis in PBS, pH 6.8, 37°C: (A) **PSY**; (B) **4a**; (C) **4b**; (D) **4c**; (E) **4d**; (F) **4e**.

A) PSY

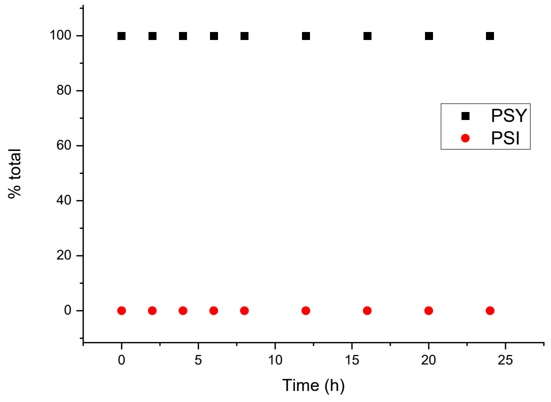

B) 4a

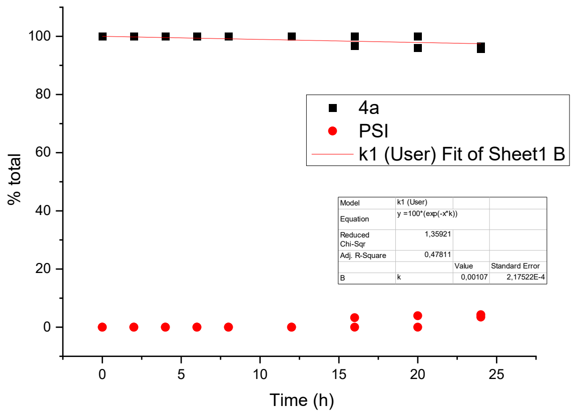

C) 4b

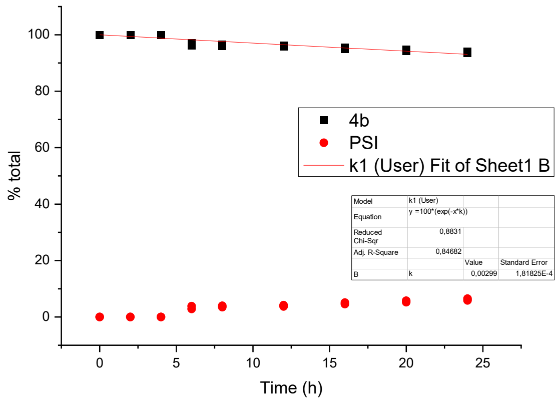
4b
PSI
k1 (User) Fit of Sheet1 B
Model k1 (User)
Equation y =100*(exp(-x*k))
Reduced Chi-Sqr 0,8831
Adj. R-Square 0,84682
Value Standard Error
B k 0,00299 1,81825E-4
% total
Time (h)

D) 4c

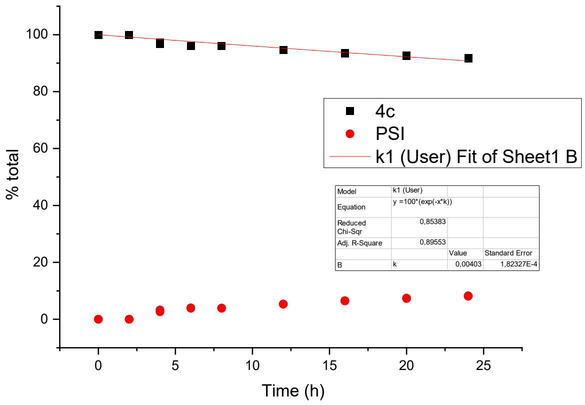
4c
PSI
k1 (User) Fit of Sheet1 B
Model k1 (User)
Equation y =100*(exp(-x*k))
Reduced Chi-Sqr 0,85383
Adj. R-Square 0,89553
Value Standard Error
B k 0,00403 1,82327E-4
% total
Time (h)

E) 4d

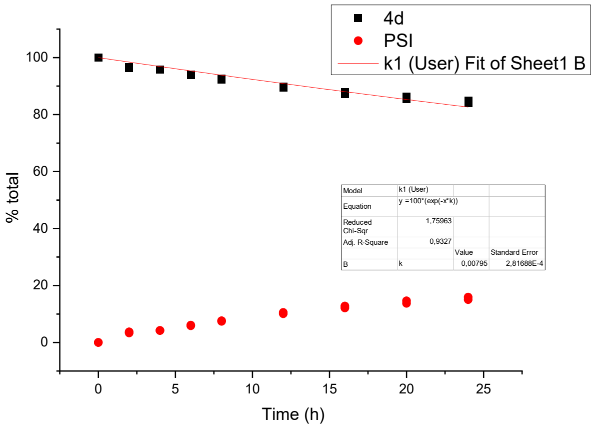
4d
PSI
k1 (User) Fit of Sheet1 B
Model k1 (User)
Equation y =100*(exp(-x*k))
Reduced Chi-Sqr 1,75963
Adj. R-Square 0,9327
Value Standard Error
B k 0,00795 2,81688E-4
% total
Time (h)

F) 4e

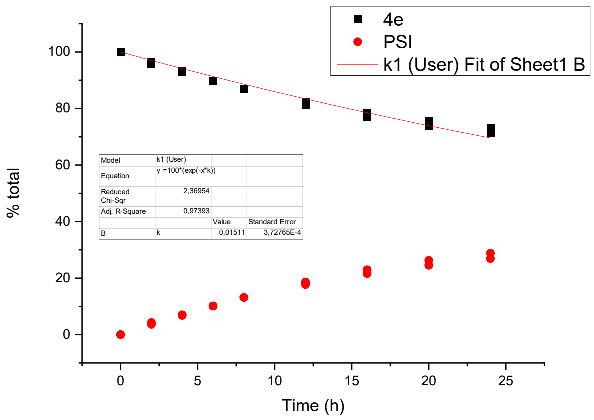

Figure S9. Interpolation of data from hydrolysis in PBS, pH 7.4, 37°C: (A) **PSY**; (B) **4a**; (C) **4b**; (D) **4c**; (E) **4d**; (F) **4e**.

A) PSY

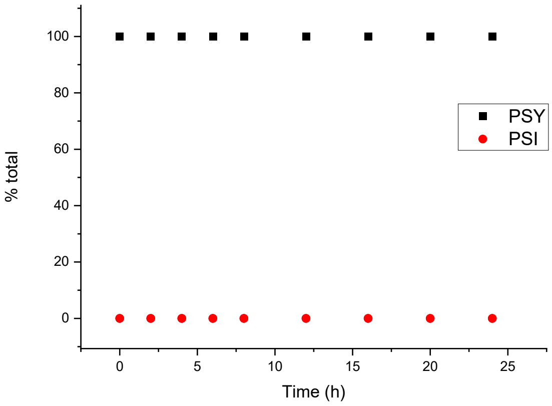

B) 4a

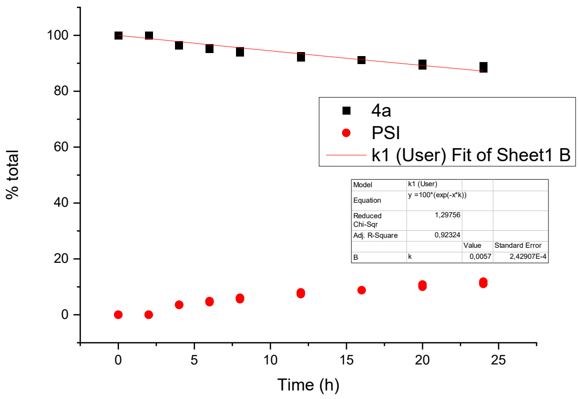

C) 4b

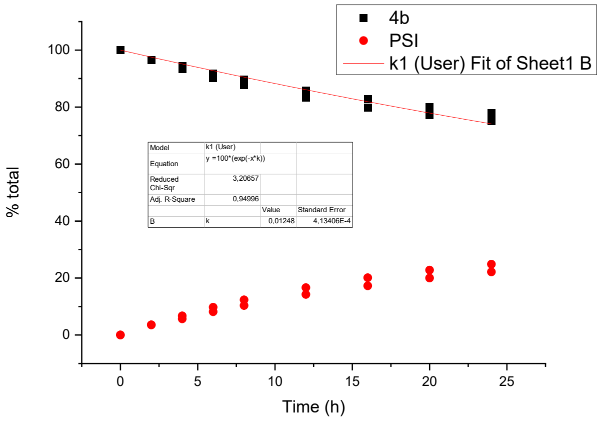
4b
PSI
k1 (User) Fit of Sheet1 B
Model | k1 (User)
Equation | y =100*(exp(-x*k))
Reduced Chi-Sqr | 3,20657
Adj. R-Square | 0,94996
Value | Standard Error
B | k | 0,01248 | 4,13406E-4
% total
Time (h)
0
5
10
15
20
25
0
20
40
60
80
100

D) 4c

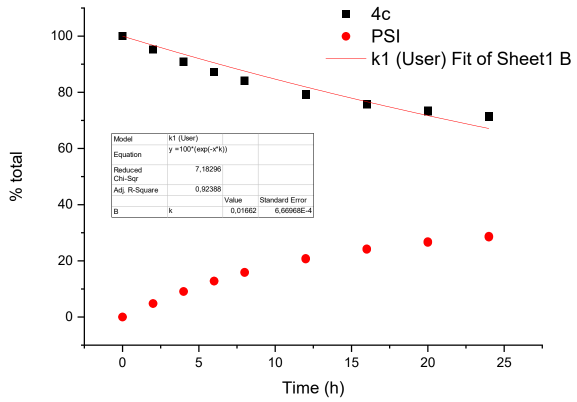
4c
PSI
k1 (User) Fit of Sheet1 B
Model | k1 (User)
Equation | y =100*(exp(-x*k))
Reduced Chi-Sqr | 7,18296
Adj. R-Square | 0,92388
Value | Standard Error
B | k | 0,01662 | 6,66968E-4
% total
Time (h)
0
5
10
15
20
25
0
20
40
60
80
100

E) 4d

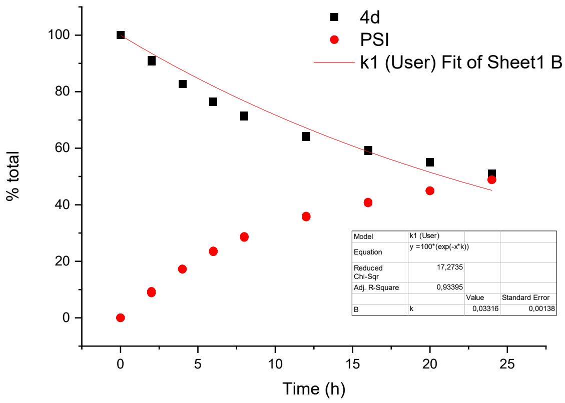
4d
PSI
k1 (User) Fit of Sheet1 B
Model | k1 (User)
Equation | y =100*(exp(-x*k))
Reduced Chi-Sqr | 17,2735
Adj. R-Square | 0,93395
Value | Standard Error
B | k | 0,03316 | 0,00138
% total
Time (h)
0
5
10
15
20
25
0
20
40
60
80
100

F) 4e

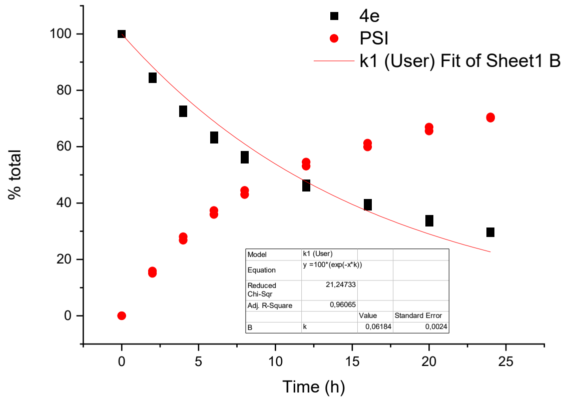

Figure S10. Interpolation of data from hydrolysis in human plasma, 37°C: (A) **PSY**; (B) **4a**; (C) **4b**; (D) **4c**; (E) **4d**; (F) **4e**.

A) PSY

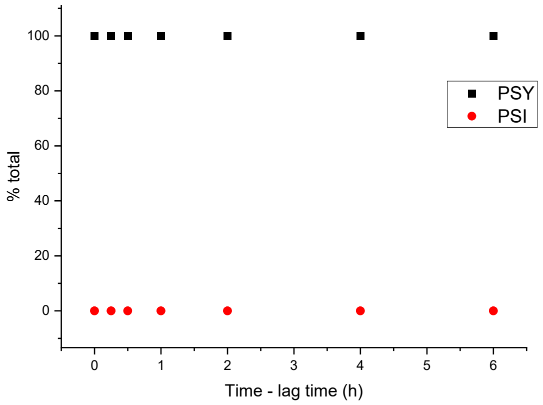

B) 4a

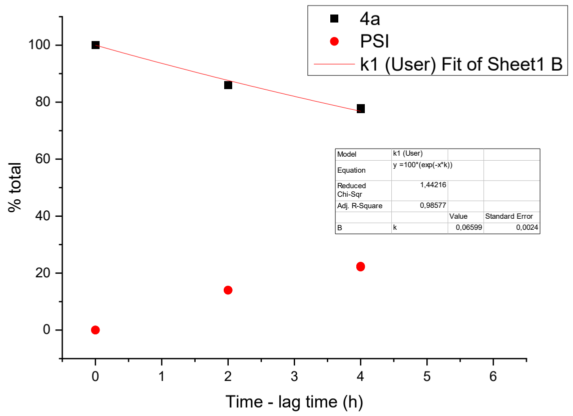

C) 4b

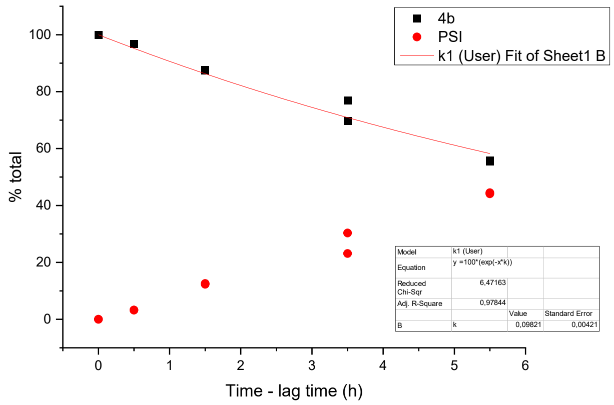
4b
PSI
k1 (User) Fit of Sheet1 B
Model | k1 (User)
Equation | y =100*(exp(-x*k))
Reduced Chi-Sqr | 6,47163
Adj. R-Square | 0,97844
Value | Standard Error
B | k | 0,09821 | 0,00421
% total
Time - lag time (h)

D) 4c

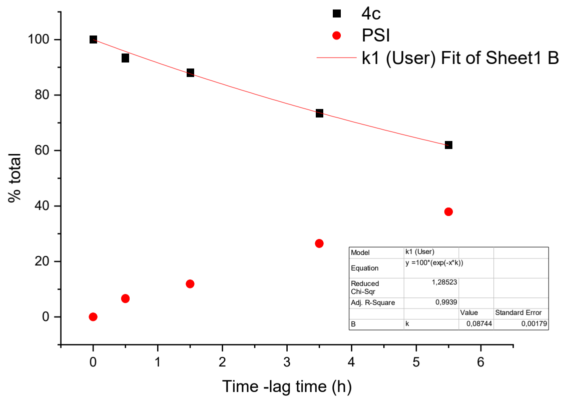
4c
PSI
k1 (User) Fit of Sheet1 B
Model | k1 (User)
Equation | y =100*(exp(-x*k))
Reduced Chi-Sqr | 1,28523
Adj. R-Square | 0,9939
Value | Standard Error
B | k | 0,08744 | 0,00179
% total
Time -lag time (h)

E) 4d

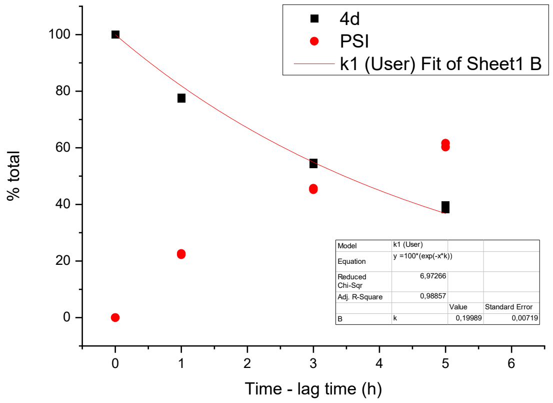
4d
PSI
k1 (User) Fit of Sheet1 B
Model | k1 (User)
Equation | y =100*(exp(-x*k))
Reduced Chi-Sqr | 6,97266
Adj. R-Square | 0,98857
Value | Standard Error
B | k | 0,19989 | 0,00719
% total
Time - lag time (h)

F) 4e

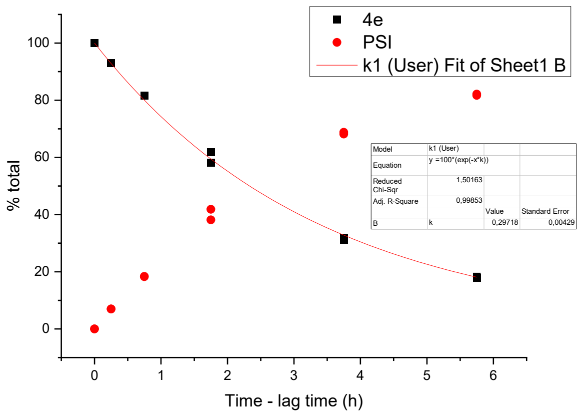

Concentration–response curves from the FLIPR calcium-flux assay of compound 4e

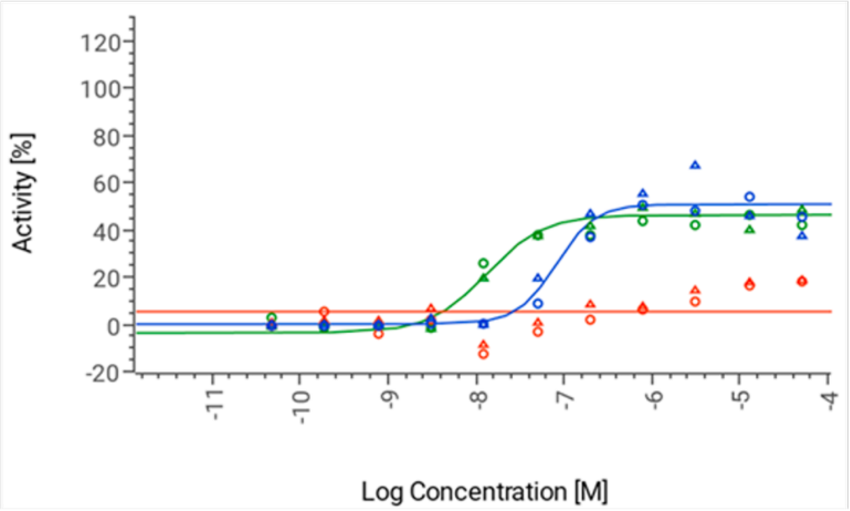

Figure S11. Partial agonist activity of **4e** at human 5-HT_2 receptor subtypes. Concentration–response curves were generated using a FLIPR calcium-flux assay in CHO-K1 cells expressing 5-HT_{2A} (green), 5-HT_{2B} (red), or 5-HT_{2C} (blue) receptors. **4e** elicited a concentration-dependent activation of 5-HT_{2A} and 5-HT_{2C} receptors, and no significant agonist activity at the 5-HT_{2B} subtype.

Preliminary qualitative identification of 4e-derived metabolites

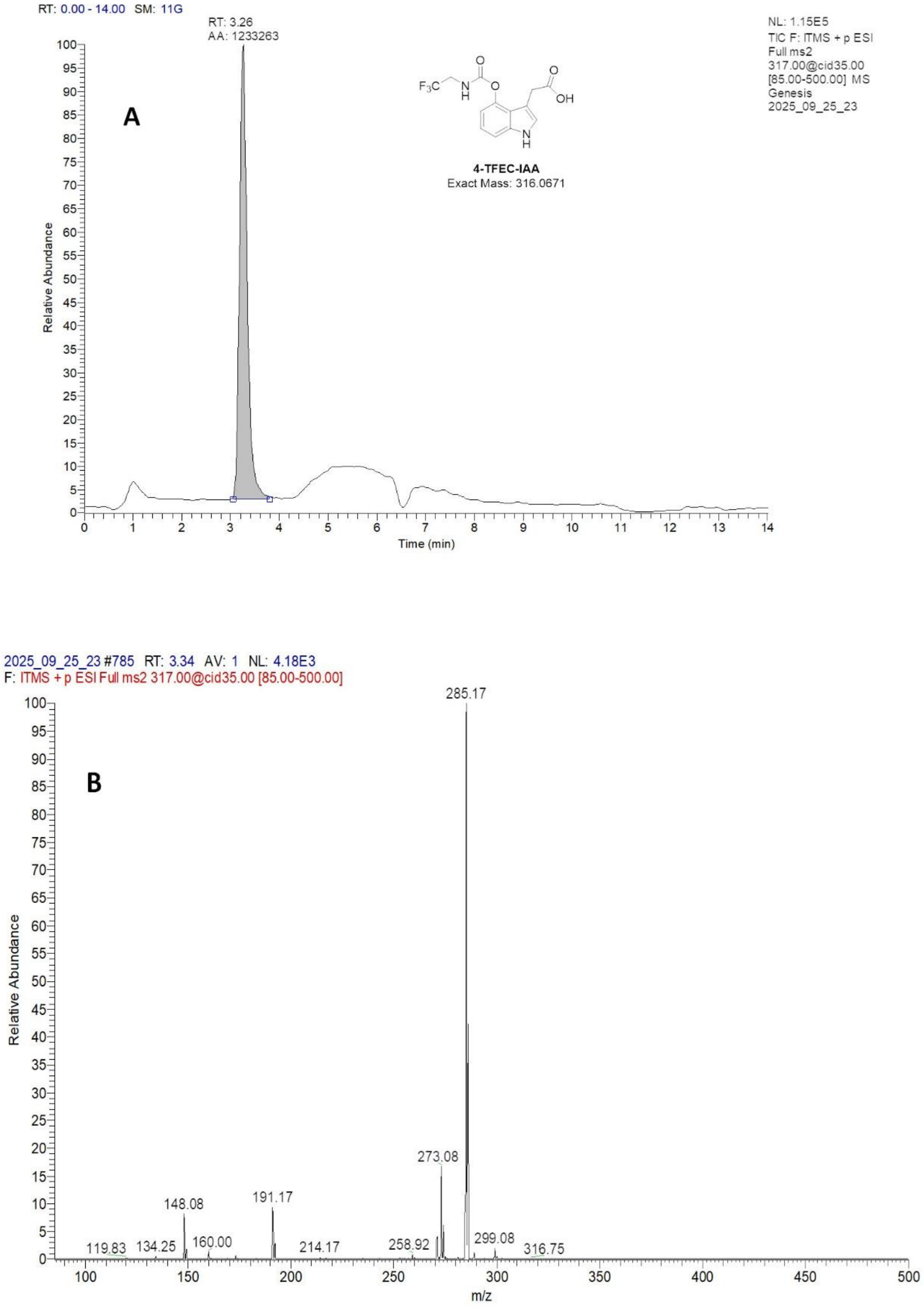

Figure S12. Representative ion chromatogram of putative metabolite 4-trifluoroethyl carbamate indoleacetic acid (**4-TFEC-IAA**) $[MH]^+$ (A) and relative ion spectra (B) in plasma sample collected 2h after administration of a single oral dose of 20 mg/kg **4e**.

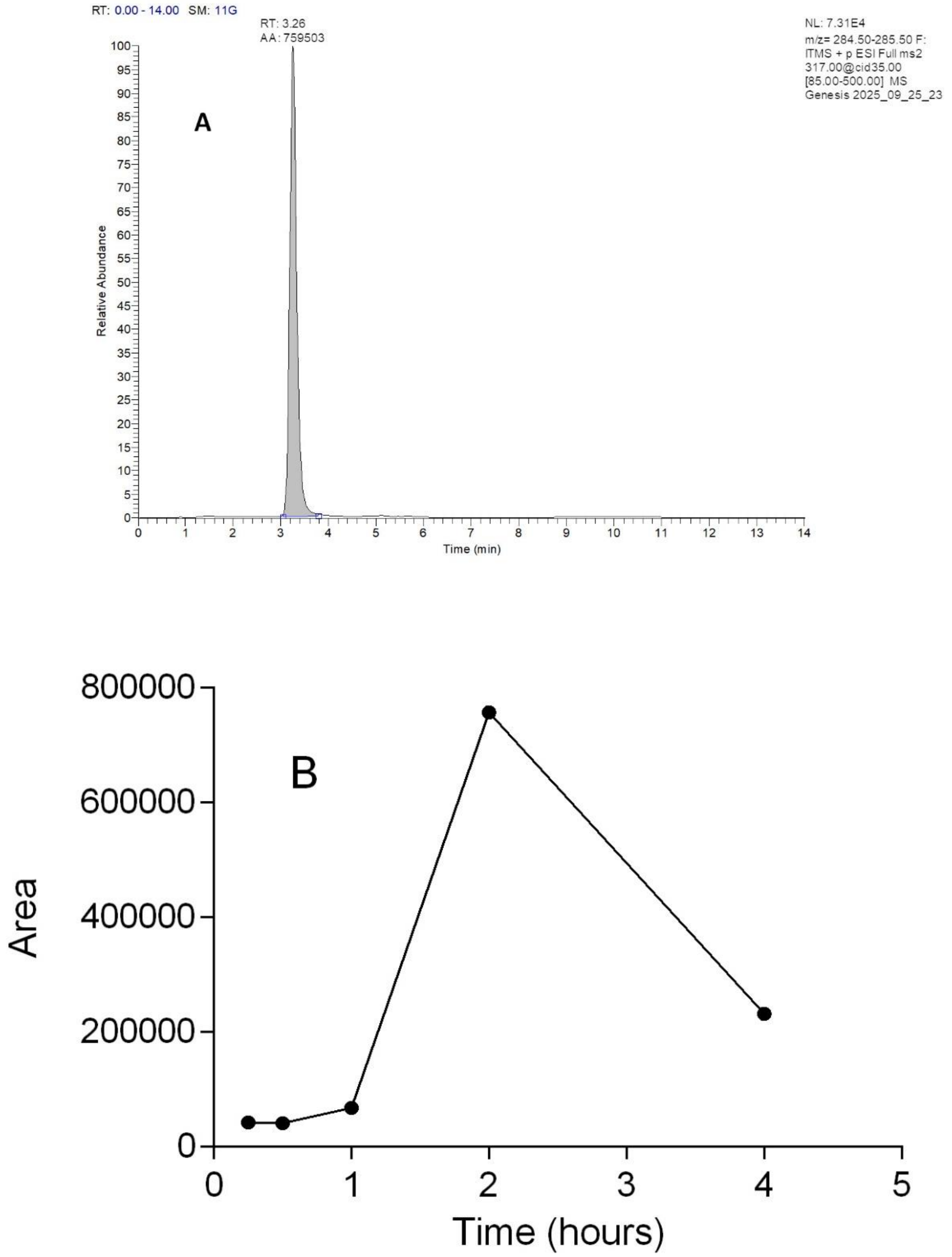

Figure S13. Representative ion chromatogram of the specific transitions monitored *m/z* 317→*m/z* 285 of the 4-trifluoroethyl carbamate indoleacetic acid (**4-TFEC-IAA**) in plasma sample collected 2 hours after administration of a single oral dose of 20 mg/kg **4e** (A) and area of the m/z 285 transition peak vs time profile (B).

Cartesian coordinates and electronic energies of compounds 4a-e and the corresponding anions. Level of theory: BLYP-D3(BJ)TZ

4a

E= -9.95694060 a.u.

NIMAG=0

C	0.091983	-0.023475	-0.649441000
N	-2.740908	2.802938	0.393439000
C	-3.672856	2.031350	1.063692000
C	-3.256681	0.713991	1.081969000
C	0.332548	1.297547	-1.082325000
C	-0.550545	2.329231	-0.775493000
C	-1.698060	2.000203	-0.045755000
C	-1.992008	0.665872	0.384483000
C	-1.047683	-0.328959	0.078791000
H	-4.564727	2.480579	1.480864000
H	-0.353389	3.350550	-1.090188000
H	-2.795504	3.805592	0.269924000
C	-4.055332	-0.463411	1.572219000
C	-4.778937	-1.066027	0.356390000
N	-5.573870	-2.348621	0.665559000
C	-4.686509	-3.507754	1.066700000
H	-4.106680	-3.226358	1.944662000
H	-5.320345	-4.367399	1.291735000
H	-4.023151	-3.716397	0.227098000
C	-6.443776	-2.726926	-0.512005000
H	-5.786740	-2.917318	-1.362609000
H	-7.013303	-3.623994	-0.261273000
H	-7.117279	-1.897377	-0.733209000
H	-6.192164	-2.140259	1.459647000
H	-5.507860	-0.354248	-0.039584000
H	-4.067752	-1.342442	-0.426633000
H	-4.782482	-0.146344	2.331991000
H	-3.385227	-1.197778	2.029529000
H	1.233754	1.511473	-1.649571000
H	0.812408	-0.808282	-0.862551000
O	-1.176402	-1.631305	0.628463000
C	-1.606978	-2.659365	-0.207507000
O	-2.396174	-2.503950	-1.134974000
N	-1.103116	-3.839429	0.231883000
C	-1.269361	-5.106203	-0.497781000
H	-2.145982	-4.986789	-1.140801000
H	-1.478752	-5.894682	0.235691000
C	-0.038964	-5.497260	-1.350882000
H	1.550942	-4.831576	0.006846000
H	-0.316878	-6.446366	-1.832464000
H	0.540602	-3.496744	-2.022370000
C	0.247597	-4.462056	-2.453209000
H	1.071201	-4.800837	-3.090570000
H	-0.630423	-4.293393	-3.086025000
C	1.202477	-5.754550	-0.476037000
H	0.999812	-6.496992	0.305703000
H	2.032181	-6.127966	-1.085414000
H	-0.372916	-3.778001	0.933443000

4a deprotonated

E= -9.96236454 a.u.

NIMAG=0

C	-0.186092	0.267692	-0.944686000

N	-2.869611	2.996562	0.646182000
C	-3.740618	2.148433	1.323384000
C	-3.309537	0.846356	1.182330000
C	0.027628	1.623582	-1.265394000
C	-0.795125	2.638869	-0.773358000
C	-1.863446	2.247071	0.042972000
C	-2.111638	0.882216	0.373107000
C	-1.238780	-0.117036	-0.114694000
H	-4.597480	2.543860	1.853325000
H	-0.617272	3.682254	-1.022237000
H	-2.950792	4.002673	0.606170000
C	-3.981798	-0.409854	1.665302000
C	-4.515579	-1.155743	0.434789000
N	-4.969942	-2.611266	0.681535000
C	-4.059517	-3.435929	1.574851000
H	-4.025978	-2.971414	2.559595000
H	-4.500918	-4.433012	1.647440000
H	-3.055413	-3.487984	1.109854000
C	-5.170560	-3.285219	-0.660796000
H	-4.223265	-3.233410	-1.203775000
H	-5.476220	-4.319688	-0.493941000
H	-5.942359	-2.738010	-1.206560000
H	-5.881912	-2.564570	1.147875000
H	-5.392725	-0.649680	0.018478000
H	-3.751643	-1.221097	-0.342317000
H	-4.789497	-0.175673	2.372423000
H	-3.238757	-1.030453	2.173824000
H	0.862882	1.884708	-1.911204000
H	0.474961	-0.499029	-1.337896000
O	-1.411027	-1.415915	0.307493000
C	-1.745452	-2.454681	-0.717457000
O	-2.261074	-2.035201	-1.777946000
N	-1.535586	-3.633500	-0.196753000
C	-1.832973	-4.773594	-1.061710000
H	-2.336905	-4.471538	-1.996183000
H	-2.511040	-5.463004	-0.523609000
C	-0.558503	-5.575052	-1.436672000
H	0.428967	-5.354385	0.486966000
H	-0.893290	-6.410657	-2.073279000
H	0.763547	-3.861123	-1.647834000
C	0.414951	-4.705996	-2.252970000
H	1.290829	-5.286978	-2.568818000
H	-0.072278	-4.298744	-3.147051000
C	0.126589	-6.161366	-0.189216000
H	-0.554352	-6.825520	0.360366000
H	1.017307	-6.741461	-0.463297000

4b

E= -9.42649103 a.u.

NIMAG=0

C	0.134201	-0.314994	-0.386595000
N	-2.602342	2.728717	0.221205000
C	-3.630096	2.057040	0.858352000
C	-3.294084	0.724503	1.000405000
C	0.486407	0.957577	-0.884265000
C	-0.355079	2.055248	-0.723163000
C	-1.575546	1.843062	-0.072616000
C	-1.982027	0.560815	0.418944000
C	-1.078501	-0.501917	0.259012000
H	-4.525210	2.581118	1.167096000

H	-0.071174	3.038891	-1.087521000
H	-2.587761	3.721738	0.027864000
C	-4.189156	-0.373764	1.506124000
C	-4.806369	-1.071300	0.283636000
N	-5.674843	-2.294999	0.632827000
C	-4.869299	-3.437872	1.211921000
H	-4.347815	-3.092421	2.103528000
H	-5.552021	-4.251203	1.464530000
H	-4.152635	-3.749475	0.451610000
C	-6.457904	-2.752670	-0.577574000
H	-5.740414	-3.036670	-1.349642000
H	-7.078028	-3.607035	-0.299205000
H	-7.080740	-1.927314	-0.926112000
H	-6.348842	-1.995048	1.348546000
H	-5.465065	-0.383113	-0.252436000
H	-4.034157	-1.443396	-0.395104000
H	-4.978228	0.039282	2.149013000
H	-3.602655	-1.081145	2.100860000
H	1.442765	1.081695	-1.383833000
H	0.822384	-1.150129	-0.483573000
O	-1.341019	-1.757457	0.875503000
C	-1.653257	-2.828101	0.050309000
O	-2.368454	-2.750882	-0.944919000
N	-1.139742	-3.976997	0.565435000
C	-1.177756	-5.232827	-0.193870000
H	-2.195131	-5.376692	-0.568272000
H	-0.952714	-6.045170	0.501967000
C	-0.217896	-5.271568	-1.401509000
F	1.578432	-3.955269	-0.488643000
H	-0.356546	-6.217460	-1.936899000
C	1.255001	-5.183453	-1.028122000
F	1.602228	-6.122405	-0.091102000
F	2.055992	-5.365429	-2.111760000
H	-0.410669	-3.862561	1.262415000
H	-0.440343	-4.453993	-2.094325000

4b deprotonated

E= -9.41453923 a.u.

NIMAG=0

C	-0.047426	-0.181000	-0.462385000
N	-2.685929	2.914623	0.342462000
C	-3.732394	2.221834	0.943895000
C	-3.408984	0.883707	1.017510000
C	0.341866	1.106625	-0.883813000
C	-0.450961	2.232796	-0.653240000
C	-1.670293	2.024970	0.003591000
C	-2.096649	0.732996	0.429553000
C	-1.253980	-0.378645	0.207808000
H	-4.623318	2.741245	1.272110000
H	-0.136521	3.221929	-0.976981000
H	-2.662924	3.912355	0.185765000
C	-4.261266	-0.255880	1.506566000
C	-4.634211	-1.111097	0.288172000
N	-5.216608	-2.505269	0.603429000
C	-4.481219	-3.288976	1.675926000
H	-4.529248	-2.731736	2.610654000
H	-4.997182	-4.245890	1.785155000
H	-3.435850	-3.450121	1.367065000
C	-5.292624	-3.294128	-0.687679000
H	-4.277689	-3.349180	-1.096808000

H	-5.693555	-4.286623	-0.472454000
H	-5.945298	-2.757962	-1.379905000
H	-6.173644	-2.363520	0.943760000
H	-5.395313	-0.615172	-0.323107000
H	-3.759428	-1.308156	-0.334964000
H	-5.156133	0.114602	2.024674000
H	-3.672986	-0.853749	2.208585000
H	1.293579	1.224451	-1.396585000
H	0.596746	-1.036582	-0.643386000
O	-1.621751	-1.601124	0.727404000
C	-1.773817	-2.740458	-0.205885000
O	-2.187801	-2.463177	-1.360143000
N	-1.565829	-3.860101	0.433116000
C	-1.748249	-5.054084	-0.386576000
H	-2.702510	-5.045727	-0.948636000
H	-1.769504	-5.925811	0.278870000
C	-0.651222	-5.270790	-1.458251000
F	1.313578	-4.478725	-0.293565000
H	-0.911210	-6.133963	-2.083765000
C	0.736982	-5.547644	-0.912858000
F	0.749468	-6.589064	-0.011063000
F	1.596698	-5.916169	-1.934012000
H	-0.588579	-4.388654	-2.103414000

4c

E= -8.78717917 a.u.

NIMAG=0

C	0.334882	0.294397	0.035873000
N	-2.879293	2.894190	0.066830000
C	-3.877767	2.102254	0.602010000
C	-3.377112	0.842299	0.870530000
C	0.563234	1.583087	-0.491024000
C	-0.448174	2.538841	-0.518228000
C	-1.704206	2.163684	-0.029408000
C	-1.984985	0.854955	0.482768000
C	-0.915928	-0.056693	0.518986000
H	-4.874032	2.496756	0.754382000
H	-0.265965	3.539497	-0.900900000
H	-2.980549	3.869056	-0.184526000
C	-4.183947	-0.351766	1.305409000
C	-4.619163	-1.103011	0.036627000
N	-5.408428	-2.398395	0.307859000
C	-4.586160	-3.447761	1.022750000
H	-4.258370	-3.052525	1.983369000
H	-5.205879	-4.333919	1.171001000
H	-3.725535	-3.671434	0.391831000
C	-5.976634	-2.951064	-0.980816000
H	-5.138950	-3.198210	-1.635593000
H	-6.562682	-3.844656	-0.757371000
H	-6.605407	-2.188656	-1.443420000
H	-6.197670	-2.151367	0.918245000
H	-5.280878	-0.476094	-0.566515000
H	-3.751271	-1.403670	-0.555578000
H	-5.063602	-0.032670	1.880993000
H	-3.574651	-0.993374	1.948730000
H	1.552878	1.835019	-0.860718000
H	1.147205	-0.424803	0.092492000
O	-1.052532	-1.306574	1.184482000
C	-1.267766	-2.442007	0.422207000
O	-1.856987	-2.462674	-0.653772000
N	-0.808810	-3.535038	1.092473000

C	-0.849797	-4.879713	0.513178000
H	-1.790779	-5.003606	-0.031049000
H	-0.819551	-5.610128	1.327259000
C	0.314853	-5.121024	-0.458100000
H	-0.234832	-3.363802	1.911144000
H	0.289751	-4.400817	-1.283587000
F	0.174817	-6.415670	-0.998899000
H	1.283072	-5.071079	0.055156000

4c deprotonated

E= -8.80199052 a.u.

NIMAG=0

C	0.089274	0.191744	-0.135157000
N	-2.867682	3.071422	0.241291000
C	-3.918189	2.309844	0.743646000
C	-3.498833	1.006559	0.906176000
C	0.419939	1.495061	-0.558047000
C	-0.483625	2.555110	-0.462369000
C	-1.750694	2.261802	0.056885000
C	-2.118065	0.950332	0.479496000
C	-1.166783	-0.091313	0.399687000
H	-4.880771	2.761085	0.946533000
H	-0.215471	3.558083	-0.785174000
H	-2.907899	4.062052	0.047424000
C	-4.313255	-0.184644	1.332868000
C	-4.487337	-1.090297	0.106538000
N	-5.031743	-2.505592	0.393737000
C	-4.421223	-3.203590	1.596197000
H	-4.651401	-2.623023	2.488958000
H	-4.883566	-4.191277	1.663086000
H	-3.330567	-3.296521	1.448154000
C	-4.874430	-3.342993	-0.859208000
H	-3.811527	-3.351421	-1.119096000
H	-5.244880	-4.350309	-0.660296000
H	-5.448745	-2.872871	-1.660432000
H	-6.034816	-2.410247	0.584434000
H	-5.191160	-0.652367	-0.608899000
H	-3.533175	-1.250507	-0.399883000
H	-5.284286	0.128299	1.740187000
H	-3.763635	-0.722874	2.110221000
H	1.411868	1.679078	-0.964169000
H	0.816667	-0.611070	-0.212956000
O	-1.487123	-1.325360	0.924046000
C	-1.508757	-2.494899	0.009790000
O	-1.736218	-2.255127	-1.198081000
N	-1.419606	-3.590481	0.722621000
C	-1.481969	-4.829247	-0.055384000
H	-2.065584	-4.738373	-0.987779000
H	-1.935016	-5.618564	0.562921000
C	-0.067868	-5.268069	-0.434061000
H	0.388913	-4.551953	-1.125906000
F	-0.101816	-6.536943	-1.099212000
H	0.558283	-5.384904	0.457147000

4d

E= -8.80228442 a.u.

NIMAG=0

C	0.306537	0.258526	-0.038964000
N	-2.871466	2.897475	0.144342000
C	-3.865503	2.105452	0.687352000

C	-3.373796	0.833737	0.914017000
C	0.535756	1.555543	-0.544273000
C	-0.462654	2.524974	-0.518336000
C	-1.708613	2.155019	-0.000842000
C	-1.991614	0.838849	0.491049000
C	-0.932679	-0.084788	0.477492000
H	-4.852871	2.507814	0.873464000
H	-0.279339	3.531267	-0.885415000
H	-2.967421	3.878626	-0.083300000
C	-4.187788	-0.362737	1.328281000
C	-4.646411	-1.070499	0.041881000
N	-5.448823	-2.363886	0.278808000
C	-4.634627	-3.443944	0.957693000
H	-4.298297	-3.080310	1.927627000
H	-5.264733	-4.326248	1.082531000
H	-3.778811	-3.656649	0.316226000
C	-6.028832	-2.870686	-1.023752000
H	-5.196913	-3.096319	-1.693466000
H	-6.615028	-3.770379	-0.826896000
H	-6.659785	-2.091577	-1.454417000
H	-6.232297	-2.126596	0.900332000
H	-5.306528	-0.416496	-0.533592000
H	-3.788848	-1.362734	-0.569146000
H	-5.056620	-0.048774	1.922487000
H	-3.579454	-1.030159	1.945975000
H	1.515985	1.801389	-0.941987000
H	1.108217	-0.474404	-0.028110000
O	-1.061779	-1.344059	1.129136000
C	-1.308672	-2.466876	0.363588000
O	-1.919090	-2.473610	-0.697413000
N	-0.853499	-3.571521	1.030999000
C	-0.828243	-4.893798	0.417863000
H	-1.565349	-4.917290	-0.389225000
H	-1.077037	-5.659221	1.160877000
C	0.538716	-5.235036	-0.192212000
H	-0.274297	-3.395322	1.845824000
F	0.853696	-4.322028	-1.181244000
F	0.452161	-6.487459	-0.771312000
H	1.368144	-5.246842	0.527482000

4d deprotonated

E= -8.82122564 a.u.

NIMAG=0

C	0.063277	0.312755	-0.269595000
N	-2.940241	3.096831	0.368757000
C	-3.945748	2.289451	0.890468000
C	-3.488282	0.991834	0.979417000
C	0.339815	1.639835	-0.656416000
C	-0.583535	2.670587	-0.472514000
C	-1.815080	2.324078	0.096902000
C	-2.129311	0.987748	0.483696000
C	-1.156355	-0.022420	0.315189000
H	-4.907709	2.707337	1.157610000
H	-0.356577	3.692420	-0.766546000
H	-3.012806	4.093356	0.219537000
C	-4.256866	-0.235848	1.388049000
C	-4.476592	-1.080736	0.125733000
N	-4.970604	-2.525388	0.361787000
C	-4.296445	-3.260887	1.505016000
H	-4.512970	-2.734100	2.433781000

H	-4.723448	-4.265943	1.538158000
H	-3.209492	-3.307418	1.319934000
C	-4.837561	-3.296327	-0.937169000
H	-3.785622	-3.252356	-1.238512000
H	-5.169120	-4.323448	-0.773481000
H	-5.459630	-2.807184	-1.689945000
H	-5.967901	-2.469783	0.593518000
H	-5.228093	-0.624928	-0.527240000
H	-3.546920	-1.190415	-0.437715000
H	-5.210770	0.036014	1.860012000
H	-3.657318	-0.799992	2.107939000
H	1.304925	1.865663	-1.103758000
H	0.805047	-0.467439	-0.413894000
O	-1.412100	-1.285272	0.812101000
C	-1.482505	-2.411447	-0.137995000
O	-1.814892	-2.135072	-1.314509000
N	-1.307202	-3.531968	0.517400000
C	-1.403266	-4.740203	-0.286727000
H	-1.447173	-4.556467	-1.372115000
H	-2.292793	-5.336129	-0.006202000
C	-0.204069	-5.636786	-0.009764000
F	0.936132	-5.151006	-0.637000000
F	-0.430970	-6.919306	-0.534344000
H	0.026562	-5.745895	1.056038000

4e

E= -8.82385239 a.u.

NIMAG=0

C	0.268541	0.212241	-0.129931000
N	-2.850604	2.874617	0.405688000
C	-3.827164	2.059717	0.946736000
C	-3.344493	0.769543	1.059410000
C	0.492089	1.538050	-0.557511000
C	-0.486787	2.516765	-0.411756000
C	-1.709518	2.129449	0.147084000
C	-1.987768	0.786579	0.562867000
C	-0.948051	-0.148204	0.426866000
H	-4.796270	2.460881	1.213558000
H	-0.304948	3.543411	-0.718163000
H	-2.942255	3.870524	0.252075000
C	-4.147390	-0.443774	1.444174000
C	-4.669502	-1.080968	0.145984000
N	-5.465097	-2.384163	0.356962000
C	-4.615438	-3.505318	0.913415000
H	-4.198442	-3.195882	1.870834000
H	-5.244862	-4.387534	1.042729000
H	-3.815948	-3.694265	0.196270000
C	-6.136979	-2.809254	-0.930451000
H	-5.354960	-2.997436	-1.668351000
H	-6.715887	-3.716595	-0.747484000
H	-6.789419	-2.002933	-1.269108000
H	-6.202730	-2.181811	1.043574000
H	-5.353803	-0.396951	-0.362312000
H	-3.845270	-1.342765	-0.522327000
H	-4.985900	-0.159414	2.094256000
H	-3.514791	-1.145345	1.996658000
H	1.454053	1.797791	-0.989488000
H	1.058061	-0.529457	-0.211652000
O	-1.069696	-1.451277	0.991681000
C	-1.329333	-2.511776	0.148562000

O -1.988471 -2.448574 -0.880960000
N -0.826823 -3.660026 0.702903000
C -0.776546 -4.907320 -0.042251000
H -1.524488 -4.873779 -0.837522000
H -0.974353 -5.759613 0.615619000
C 0.596802 -5.132740 -0.695510000
H -0.179394 -3.541138 1.476537000
F 1.585742 -5.167702 0.252052000
F 0.610850 -6.316860 -1.363615000
F 0.909937 -4.140252 -1.573432000

4e deprotonated

E= -8.85305709 a.u.

NIMAG=0

C -0.041187 0.701574 -0.699322000
N -3.213440 3.063706 0.563918000
C -4.034805 2.117149 1.164077000
C -3.438370 0.876149 1.077230000
C 0.028762 2.082295 -0.984988000
C -0.967854 2.980996 -0.609339000
C -2.065998 2.445605 0.077392000
C -2.178845 1.059386 0.385529000
C -1.141448 0.174114 -0.015814000
H -4.974311 2.405019 1.617618000
H -0.896617 4.041330 -0.838640000
H -3.404347 4.054508 0.515271000
C -3.988145 -0.420044 1.609263000
C -4.285996 -1.373639 0.447052000
N -4.699767 -2.785680 0.880866000
C -3.683333 -3.455531 1.788644000
H -3.615679 -2.890832 2.717473000
H -4.032415 -4.471263 1.983844000
H -2.732230 -3.463919 1.231878000
C -4.931702 -3.631463 -0.350926000
H -3.977566 -3.683843 -0.878184000
H -5.250215 -4.628478 -0.042011000
H -5.697663 -3.151945 -0.963706000
H -5.585326 -2.709688 1.393538000
H -5.105825 -0.996660 -0.172207000
H -3.388049 -1.520964 -0.153429000
H -4.895475 -0.229356 2.200458000
H -3.241757 -0.875202 2.268054000
H 0.901757 2.452678 -1.517961000
H 0.756410 0.034814 -0.998122000
O -1.351861 -1.143416 0.312848000
C -0.609677 -2.269547 -0.318003000
O 0.582688 -2.134153 -0.597346000
N -1.451836 -3.287747 -0.415262000
C -0.781650 -4.473756 -0.928322000
H 0.006981 -4.852545 -0.255780000
H -0.299918 -4.313183 -1.906855000
C -1.764114 -5.614737 -1.118209000
F -2.745005 -5.337070 -2.048687000
F -1.136310 -6.753857 -1.546746000
F -2.435772 -5.953226 0.043039

Histological evaluation of organ toxicity following administration of compound 4e

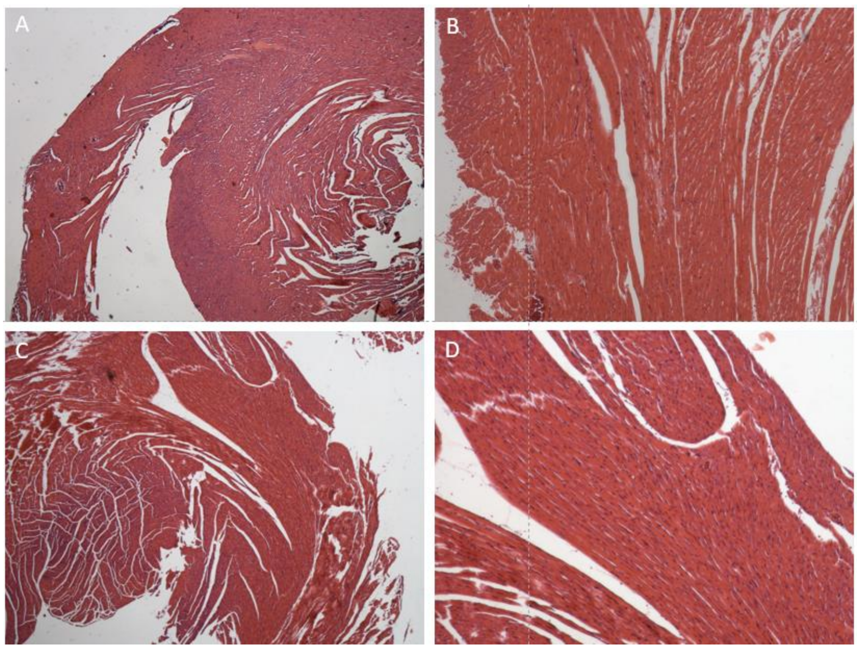

Figure S14. Representative heart histology (hematoxylin and eosin staining, 10X magnification (A, C) and, 20X magnification (B, D)) from rats treated with vehicle (A, B) or **4e** (C, D).

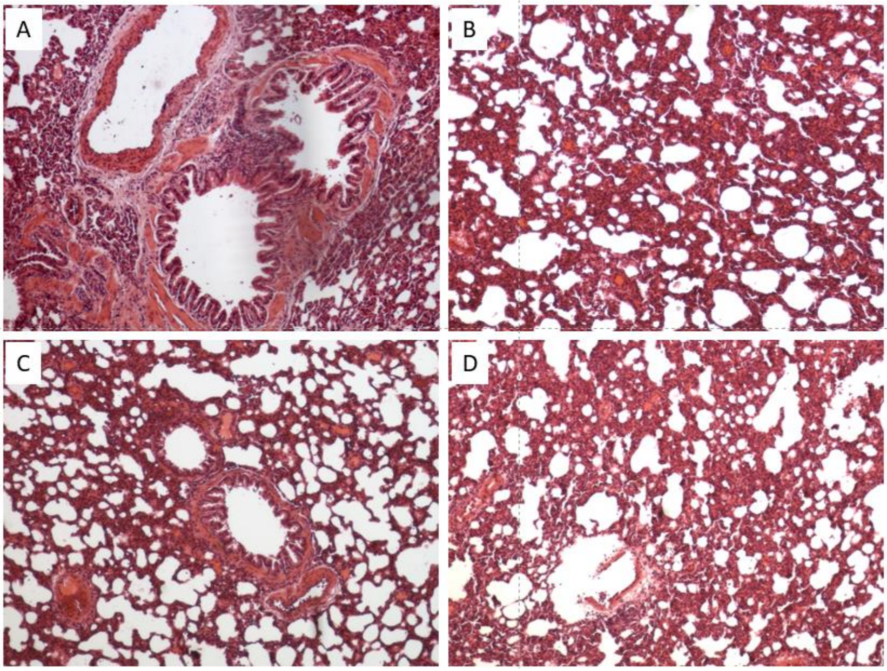

Figure S15. Representative lung histology (hematoxylin and eosin staining, 10X magnification) showing bronchi (A, C) and alveoli (B, D) from rats treated with vehicle (A, B) or **4e** (C, D). .
